# Electrostatically Interacting Wannier Qubits in Curved Space

**DOI:** 10.3390/ma17194846

**Published:** 2024-09-30

**Authors:** Krzysztof Pomorski

**Affiliations:** 1 Faculty of Technical Physics, Information Technology and Applied Mathematics, Institute of Physics, Lodz University of Technology, 93-005 Lodz, Poland; kdvpomorski@gmail.com; 2 Quantum Hardware Systems, 94-056 Lodz, Poland

**Keywords:** tight-binding model, Wannier qubit, position-based qubit, q-electrostatic gates, geometric dissipation, Aharonov–Bohm effect in curved space, geometrically induced position-based qubits

## Abstract

A derivation of a tight-binding model from Schrödinger formalism for various topologies of position-based semiconductor qubits is presented in the case of static and time-dependent electric fields. The simplistic tight-binding model enables the description of single-electron devices at a large integration scale. The case of two electrostatically Wannier qubits (also known as position-based qubits) in a Schrödinger model is presented with omission of spin degrees of freedom. The concept of programmable quantum matter can be implemented in the chain of coupled semiconductor quantum dots. Highly integrated and developed cryogenic CMOS nanostructures can be mapped to coupled quantum dots, the connectivity of which can be controlled by a voltage applied across the transistor gates as well as using an external magnetic field. Using the anti-correlation principle arising from the Coulomb repulsion interaction between electrons, one can implement classical and quantum inverters (Classical/Quantum Swap Gate) and many other logical gates. The anti-correlation will be weakened due to the fact that the quantumness of the physical process brings about the coexistence of correlation and anti-correlation at the same time. One of the central results presented in this work relies on the appearance of dissipation-like processes and effective potential renormalization building effective barriers in both semiconductors and in superconductors between not bended nanowire regions both in classical and in quantum regimes. The presence of non-straight wire regions is also expressed by the geometrical dissipative quantum Aharonov–Bohm effect in superconductors/semiconductors when one obtains a complex value vector potential-like field. The existence of a Coulomb interaction provides a base for the physical description of an electrostatic Q-Swap gate with any topology using open-loop nanowires, with programmable functionality. We observe strong localization of the wavepacket due to nanowire bending. Therefore, it is not always necessary to build a barrier between two nanowires to obtain two quantum dot systems. On the other hand, the results can be mapped to the problem of an electron in curved space, so they can be expressed with a programmable position-dependent metric embedded in Schrödinger’s equation. The semiconductor quantum dot system is capable of mimicking curved space, providing a bridge between fundamental and applied science in the implementation of single-electron devices.

## 1. Classical vs. Quantum Picture in Programmable Matter

Most methodologies used in descriptions of materials and material processing use paradigms from classical physics, whereby a given particle position is localized in one geometrical point in space and has a well-defined trajectory. Currently, we can engineer materials on a large industrial scale, producing meta-lattices of certain periodicity or with certain non-periodic features and the sharp trajectory of a given particle is not always the case as many perturbing factors might contribute to system noise and thus the particle no longer has a sharp trajectory and is not always localized, but is partly delocalized as can be given by classical statistical physics and quantum physics pictures.

In general, we can build in or induce certain symmetries or break certain symmetries in certain materials. We can confine particles within certain tunnels of a given curvature or into certain geometrical areas, so we can control the dynamics of particle movement. We can use the given particles and control their dynamics for information processing. However, using the framework of classical statistical physics instead of sharp trajectories already introduces stochastic determinism in a certain class of trajectories dealing with probabilities. Once we have achieved a process resulting in a highly integrated meta-material with a certain periodicity or aperiodicity, it is inevitable that we will encounter certain parameter deviations that can be well described by classical statistical physics. Further qualitative features are introduced by moving from the macroscopic to the mesoscopic and nanoworld scale, which takes place when tiny material structures are incorporated by lithographic processes. In such cases, it is necessary to use a quantum mechanics methodology that is deeply embedded in classical statistical physics methodology, with new added features such as entanglement or phase coherence. On the other hand, due to the current global competition to implement quantum computation, quantum sensing, and quantum communication achievable with certain material architectures and expressed by qubits, one needs a relatively simple but still valid mathematical platform for the effective description of system properties. This will be provided by the Schrödinger equation in curved space described in this work, which can give valid descriptions of certain integrated structures. Due to the scalability of single-electron devices in semiconductors and their possible prominent role in future quantum computers [1,2,3,4], we focus on position-based qubits in curved space. The simplest mathematical picture of system dynamics occurs in quasi-one-dimensional systems, which are our main interest. Before entering into detailed cases, we will first discuss the philosophy behind using classical and quantum logic.

## 2. Philosophy Behind Charged-Based Classical and Quantum Logic

**Figure 1:**
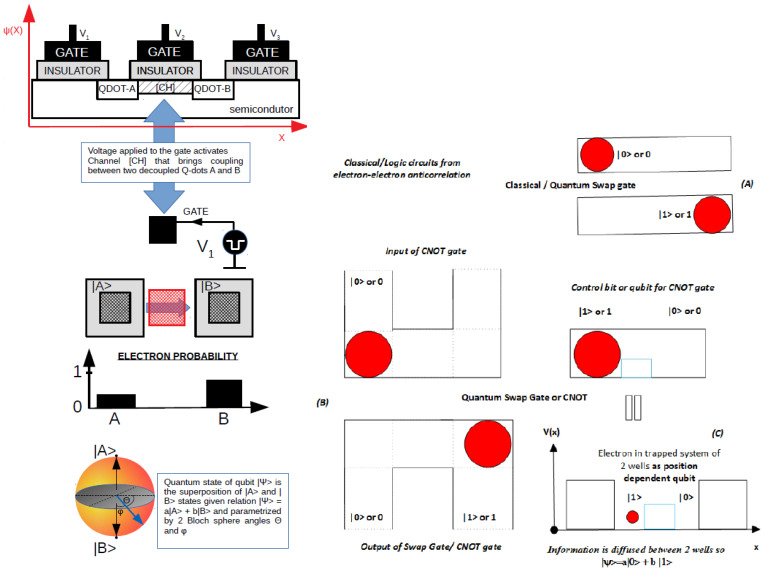
**Left** (**A**): Position-based qubit, also known as a Wannier qubit, in a CMOS circuit as implementated in [14,15,16,23]. **Right**: (**B**) Electrostatic inverter (Quantum Swap Gate) made from position-based qubits, also known as Wannier qubits; (**C**): Controllable NOT gate. The generalized version of an electrostatic double quantum dot system (qubit) is given in Figure 3 and a generalized Quantum Swap Gate is depicted in Figure 4.

**Figure 2:**
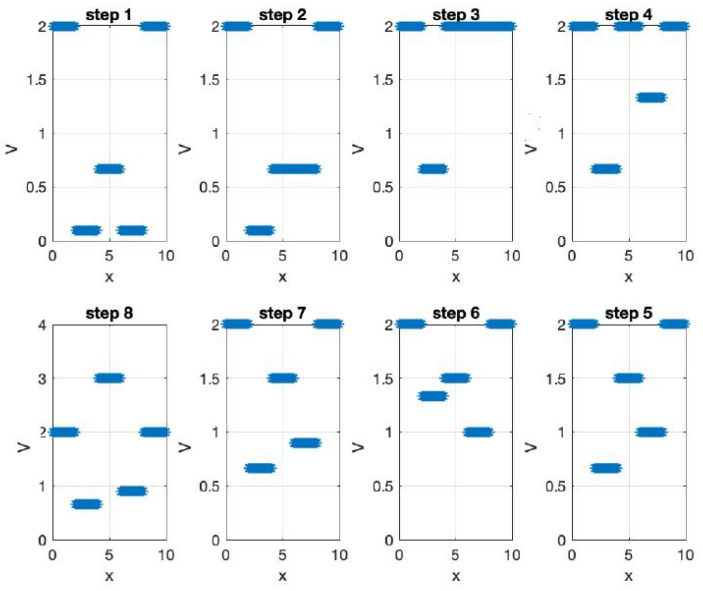

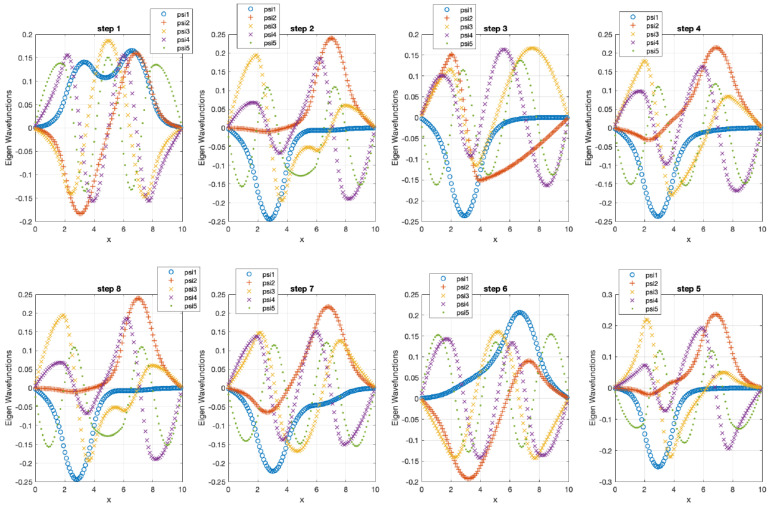
**Upper page**: Effective potentials V(x) for a single electron under different voltage biasing circumstances [24]. **Current page**: Electron wave−functions for aforementioned effective potentials with subsequent different effective eigenergy [24] obtained by Wannier qubit in different electrostatic polarization. Maximum localized functions can be constructed for various qubit electrostatic biasing potentials expressed by effective potential.

Single electron devices in semiconductor quantum dots [5], as depicted in Table 1, are a quite promising way of implementing qubit and quantum computation as well as quantum communication, as confirmed experimentally by [6,7]. This is a particularly attractive perspective in the framework of CMOS technology [1,2,8,9,10,11,12,13,14,15,16]. In the case of small field effect transistors, the source and drain play the role of quantum dots, the connectivity of which is regulated by a voltage applied on the top gate that stands in between, as depicted in Figure 1 and Figure 2. We consider only single-electron devices, in which one electron occupies a single channel, which is assumed to be a quasi-one-dimensional nanowire [17]. It is natural to expect the case of two coupled quantum dot system oscillations of occupancy in left and right quantum dots when at least two eigenergy levels are occupied. In the case of a time-independent Hamiltonian, corresponding to a time-independent magnetic and electric field as well as to time independent boundary conditions (guaranteed by stiffness of nanostructure), we have a system wave-function that can be written as a linear combination of the maximum localized left and right wave-functions (written as the linear combination of two eigenergy wavefunctions that are orthonormal). In condensed matter physics, Wannier functions are known in the case of elementary cells, accounting for crystal lattices. Such Wannier functions are maximum localized on particular atoms and orthonormal to each other, as described in [18,19,20]. Moreover, the derivation of the Aharonov–Bohm [21] effect in curved space implies the existence of a complex-value vector potential field and is conducted in this work.

**Table 1:** Comparison of various technologies implementing qubits and corresponding decoherence times [22].

Modality	Superconducting	Trapped Ion	Photonic	Neutral Atom	Silicon Spin
# Qubits	127Q	32Q	20 Photons: 216 Qumode	100Q	2Q
T2 Lifetime	Short: 15 μs–256 μs	Long: 0.2 s–50 s	Short: 150 μs	Long: 0.2 s–10 s	Mixed: 1 μs–0.5 s
2Q Gate Fidelity	High: 99–99.7%	High: 98.5–99.92%	Promising: 98%	Promising: 97.4%	Promising: 90–98%
Gate Speed	Fast: 10 ns–196 ns	Mixed: 1 μs–3 ms	Very Fast: 1 ns	Medium: 1 μs	Fast: 0.8–80 ns

The simplest model describing a semiconductor Wannier position-based qubit is given by a simplistic tight-binding model. The dynamics of the quantum state with time are given by

(1) $$\begin{array}{c} {\hat{H}}_{t}|\psi (t)\rangle =\left(\begin{array}{cc} E_{p1}\left(t\right) & t_{s12}\left(t\right)\\ t_{s12}^{*}\left(t\right) & E_{p2}\left(t\right) \end{array}\right)\left(\begin{array}{c} \alpha_{q}\left(t\right)\\ \beta_{q}\left(t\right) \end{array}\right)=\\ \left(\frac{1}{2}\left(E_{p1}\left(t\right)+E_{p2}\left(t\right)\right){\hat{\sigma }}_{0}+\frac{1}{2}\left(E_{p1}\left(t\right)-E_{p2}\left(t\right)\right){\hat{\sigma }}_{3}+\frac{1}{2}\left(t_{s12}\left(t\right)+\left(t_{s12}{\left(t\right)}^{*}\right){\hat{\sigma }}_{1}+\frac{1}{2}\left(t_{s12}\left(t\right)-t_{s12}^{*}\left(t\right)\right){\hat{\sigma }}_{2}\right)\right)\left(\begin{array}{c} \alpha_{q}\left(t\right)\\ \beta_{q}\left(t\right) \end{array}\right)=\\ =\left[E_{pot}{\hat{\sigma }}_{0}+B_{eff,x}{\hat{\sigma }}_{1}+B_{eff,y}{\hat{\sigma }}_{2}+B_{eff,z}{\hat{\sigma }}_{3}\right]\left(\begin{array}{c} \alpha_{q}\left(t\right)\\ \beta_{q}\left(t\right) \end{array}\right)=i\hbar \frac{d}{dt}\left(\begin{array}{c} \alpha_{q}\left(t\right)\\ \beta_{q}\left(t\right) \end{array}\right)=i\hbar \frac{d}{dt}|\psi (t)\rangle =i\hbar \frac{d}{dt}\left(\alpha_{q}\left(t\right)|w_{L}\left(x\right)\rangle +\beta_{q}\left(t\right)|w_{R}\left(x\right)\rangle \right),\\ |\alpha_{q}{\left(t\right)|}^{2}+{\left|\beta_{q}\left(t\right)\right|}^{2}=1, \end{array}$$

where $E_{p1}$ denotes the maximum localized energy due to the presence of an electron on the left quantum dot, $E_{p2}$ denotes the maximum localized energy due to the presence of the electron on the right quantum dot, and $|t_{s12}|$ is hopping energy due to the movement of the electron from the left to right quantum dot. We can refer to spin in an external magnetic field since $H=\vec{\sigma }\cdot \vec{B}$, so we have an effective but not fully real magnetic field component equivalent to $B_{eff,x}=\frac{1}{2}(t_{s12}\left(t\right)+\left(t_{s12}{\left(t\right)}^{*}\right)$, $B_{y,eff}=\frac{1}{2}\left(t_{s12}\left(t\right)-t_{s12}^{*}\left(t\right)\right)$, $B_{eff,z}=\frac{1}{2}\left(E_{p1}\left(t\right)-E_{p2}\left(t\right)\right)$, where $\sigma_{k}$ are Pauli matrices. We can notice that potential energy $E_{p}$ can always be electrostatically regulated to be zero by setting the proper voltage of the polarizing gates. Therefore, the presented position-based qubit can be mathematically mapped to the spin-like qubit commonly used in most common quantum technologies. Furthermore, using a tight-binding model describing two electrostatically coupled position-based qubits (two double quantum dot systems) and using the same correlation function applicable in the test of Bell theory of entangled spins, one can obtain the correlation functions as given by [14].

**Figure 3:**
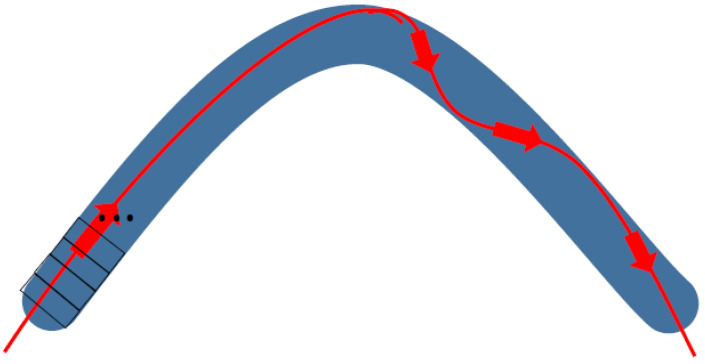
Schematic movement of wave-packet across a curved nanowire that can be simplified as a quasi-one-dimensional object after proper transformation from 3D or 2D to 1D (dimension), visualized by Marcin Piontek.

**Figure 4:**
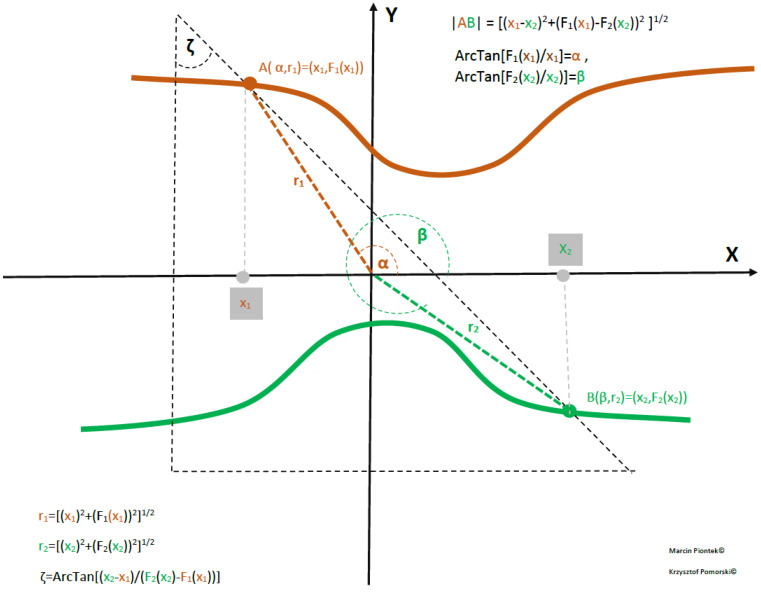
Generalized Q−Swap gate (Q−Inverter) for the case of two bent semiconductor or superconducting nanowires.

We can derive the tight-binding formalism from the Schrödinger formalism with the assumption that every Schrödinger wavefunction is a linear combination of two maxima localized on the left and right quantum dot/quantum area wavefunctions, which is given by equations

(2) $$\begin{array}{c} \psi \left(x,t\right)=\alpha_{q}\left(t\right)w_{L}\left(x\right)+\beta_{q}\left(t\right)w_{R}\left(x\right),\int_{-\infty }^{+\infty }dx{\left|w_{L(R)}\left(x\right)\right|}^{2}=1,\int_{-\infty }^{+\infty }dxw_{R}^{*}\left(x\right)w_{L}\left(x\right)=\langle w_{R}||w_{L}\rangle =0,\\ \alpha_{q}\left(t\right)=\int_{-\infty }^{+\infty }dxw_{L}^{*}\left(x\right)\psi \left(x,t\right)=\langle w_{L}||\psi \rangle ,\beta_{q}\left(t\right)=\int_{-\infty }^{+\infty }dxw_{R}^{*}\left(x\right)\psi \left(x,t\right)=\langle w_{L}||\psi \rangle , \end{array}$$

where $w_{L}\left(x\right)$ and $w_{R}\left(x\right)$ are maximum localized orthonormal wavefunctions (Wannier functions) of a single electron on the left or right quantum dot. Wannier functions are defined by Schrödinger wavefunction distribution implementing maximum occupancy of the electron on the left or right side and are a linear combinations of two or more eigenergy wavefunctions. A description of the tight-binding model in terms of Wannier functions is given by

(3) $$\begin{array}{c} E_{p1(p2)}=\int_{-\infty }^{+\infty }dxw_{L(R)}{\left(x\right)}^{*}\left(\frac{-\hbar^{2}}{2m}\frac{d^{2}}{dx^{2}}+V\left(x\right)\right)w_{L(R)}\left(x\right)=\int_{-\infty }^{+\infty }dxw_{L(R)}{\left(x\right)}^{*}\hat{H}\left(x\right)w_{L(R)}\left(x\right),\\ t_{s12(s21)}=\int_{-\infty }^{+\infty }dxw_{L(R)}{\left(x\right)}^{*}\left(\frac{-\hbar^{2}}{2m}\frac{d^{2}}{dx^{2}}+V\left(x\right)\right)w_{R(L)}\left(x\right)=\int_{-\infty }^{+\infty }dxw_{L(R)}{\left(x\right)}^{*}\hat{H}\left(x\right)w_{R(L)}\left(x\right), \end{array}$$

where $\hat{H}$ is a Hamiltonian of double quantum dot system. In this work, we justify the formulas for $E_{p1}$, $E_{p2}$, $t_{s12}$, and $t_{s21}$ from the most general case [25]. Physics relies on the principles of the conservation of mass and electric or topological charge. Both quantities are quasi-continuous on the macroscale and on the nanoscale become integer multiplicity of elementary values. The charge flows in such a way that minimizes the energy of the electric and magnetic fields. One result is the repulsion of two charges of the same sign and attraction of two charges of opposite signs, which is commonly known as Coulomb’s law. The electric current flow also tends to minimize the energy of magnetic field, so the state of most perfect equilibrium in an isolated capacitor is in a discharged device. Due to electron and hole mobility, the charge can be used for information or energy transfer across metallic or semiconductor nanowires. One can use the electric and magnetic fields as parameters controlling the evolution of the given physical system over time, so a desired final state can be achieved by setting the system in an initial configuration formally expressed by circuit theory in both the classical and quantum regimes. The simple rules of dynamics of charged billiard balls confined in boxes can lead to a simple scheme for the implementation of logical operations as a logical inverter (Quantum Swap Gate) or controllable inverter (CNOT gate or Controllable Quantum Swap Gate), as depicted in Figure 1 and described by [1,2,5,8,14,15]. However, the electric charge has been confirmed experimentally to be quantized (except for the fractional quantum Hall effect, where fractionation of the electric charge is observed), and expressed by electron, proton, or hole charge in condensed matter systems. The quantization and control of single electron flow by distinct integer values can be achieved in nanotechnological experiments, as in the chain of coupled quantum dots which can have particularly small diameters in semiconductors and in most recent CMOS technology. Sizes of 3 nm can be achieved for very highly integrated circuits. In such structures, the use of magnetic fields is less practical since waveguides and solenoids are very difficult to scale, since they occupy relatively big space. Therefore, it is preferable to use only electric fields as a controlling factor as with use of metallic wires, which favors Wannier qubits, also known as position-based qubits. Wannier qubits use maximum localized wavefunctions, such as occur in two coupled quantum dots, in order to encode quantum information in the qubit. This differentiates such qubits from eigenergy-based qubits, which use two eigenergies to span the qubit state. However, it should be emphasized that even under cryogenic conditions semiconductors have intrinsic noise, which is significantly higher than in the case of superconductors. Our considerations with cable curvature will concentrate on semiconducting cables with various shapes with examples specified by (Figure 3 and Figure 4).

## 3. From Schrödinger to Wannier Functions

Let us consider a system with two coupled quantum dots. Let us assign the occupancy of the left quantum dot by an electron as wave-packet presence in x∈(−∞,0) and wavepacket occupancy of the right quantum dot as wave-packet presence in x∈(0,+∞). We can assume that the Wannier wave-functions are linear transformations of system eigenenergy wave-functions.

We propose maximum localized orthonormal Wannier functions of the form

(4) $$\begin{array}{c} w_{L}\left(x\right)=\left(+\alpha \psi_{E1}\left(x\right)+\beta \psi_{E2}\left(x\right)\right)=w_{1,1}\psi_{E1}\left(x\right)+w_{1,2}\psi_{E2}\left(x\right),\\ w_{R}\left(x\right)=\left(-\beta \psi_{E1}\left(x\right)+\alpha \psi_{E2}\left(x\right)\right)=w_{2,1}\psi_{E1}\left(x\right)+w_{2,2}\psi_{E2}\left(x\right), \end{array}$$

and formally we have transformation between the eigenenergy base and Wannier function base in the form

(5) $$\begin{array}{c} \left(\begin{array}{c} w_{L}\left(x\right)\\ w_{R}\left(x\right) \end{array}\right)=\hat{W}\left(\begin{array}{c} \psi_{E1}\left(x\right)\\ \psi_{E2}\left(x\right) \end{array}\right)=\left(\begin{array}{cc} w_{1,1} & w_{1,2}\\ w_{2,1} & w_{2,2} \end{array}\right)\left(\begin{array}{c} \psi_{E1}\left(x\right)\\ \psi_{E2}\left(x\right) \end{array}\right). \end{array}$$

We have four conditions expressing the orthonormality of Wannier functions:

(6) $$\begin{array}{c} 1=\int_{-\infty }^{+\infty }dxw_{L}^{*}\left(x\right)w_{L}\left(x\right)=\int dx\left(+\alpha^{†}\psi_{E1}{\left(x\right)}^{†}+\beta^{†}\psi_{E2}{\left(x\right)}^{†}\right)\left(\alpha \psi_{E1}\left(x\right)+\beta \psi_{E2}\left(x\right)\right),\\ 1=\int_{-\infty }^{+\infty }dxw_{R}^{*}\left(x\right)w_{R}\left(x\right)=\int dx\left(-\beta^{†}\psi_{E1}\left(x\right)+\alpha^{†}\psi_{E2}{\left(x\right)}^{†}\right)\left(-\beta \psi_{E1}\left(x\right)+\alpha \psi_{E2}\left(x\right)\right),\\ 0=\int_{-\infty }^{+\infty }dxw_{R}^{*}\left(x\right)w_{L}\left(x\right)=\int dx\left(-\beta^{†}\psi_{E1}{\left(x\right)}^{†}+\alpha^{†}\psi_{E2}{\left(x\right)}^{†}\right)\left(\alpha \psi_{E1}\left(x\right)+\beta \psi_{E2}\left(x\right)\right),\\ 0=\int_{-\infty }^{+\infty }dxw_{L}^{*}\left(x\right)w_{R}\left(x\right)=\int dx\left(\alpha^{†}\psi_{E1}{\left(x\right)}^{†}+\beta^{†}\psi_{E2}{\left(x\right)}^{†}\right)\left(-\beta \psi_{E1}\left(x\right)+\alpha \psi_{E2}\left(x\right)\right). \end{array}$$

Due to the orthogonality of the wavefunctions $\psi_{E1}$ and $\psi_{E2}$, we have from the first two equations ${\left|\alpha \right|}^{2}+{\left|\beta \right|}^{2}=1$, so ${\left|\beta \right|}^{2}=1-{\left|\alpha \right|}^{2}$ and hence |α|=cos(γ) and |β|=sin(γ). From the third and fourth equation, we have $\alpha^{†}\beta =\alpha \beta^{†}$, which is fulfilled when $\alpha =\left|\alpha \right|e^{i\delta },$ $\beta =\left|\beta \right|e^{+i\delta }=\sqrt{1-{\left|\alpha \right|}^{2}}e^{+i\delta }$.

We propose maximum localized orthonormal Wannier functions of the form

(7) $$\begin{array}{c} w_{L}\left(x\right)=\left(+|\alpha \right|e^{i\delta }\psi_{E1}\left(x\right)+\sqrt{1-{\left|\alpha \right|}^{2}}e^{+i\delta }\psi_{E2}\left(x\right)),\\ w_{R}\left(x\right)=(-\sqrt{1-{\left|\alpha \right|}^{2}}e^{+i\delta }\psi_{E1}\left(x\right)+\left|\alpha \right|e^{i\delta }\psi_{E2}\left(x\right)), \end{array}$$

The last criteria to be matched is that $w_{L}\left(x\right)$ is maximum localized on the left quantum dot, so its geometric position is given by x∈(−∞,0), and $w_{R}\left(x\right)$ is maximum localized on the right quantum dot, so x∈(0,+∞). Formally, we can define

(8) $$\begin{array}{c} S_{L}\left(\alpha \right)\left[\psi_{E1}\left(x\right),\psi_{E2}\left(x\right)\right]=S_{L}\left(\gamma \right)\left[\psi_{E1}\left(x\right),\psi_{E2}\left(x\right)\right]=\int_{-\infty }^{0}w_{L}{\left(x\right)}^{*}w_{L}\left(x\right)dx=\\ =\int_{-\infty }^{0}dx[+|\alpha |\psi_{E1}^{†}\left(x\right)+\sqrt{1-{\left|\alpha \right|}^{2}}\psi_{E2}^{†}\left(x\right))][+\left|\alpha \right|\psi_{E1}\left(x\right)+\sqrt{1-{\left|\alpha \right|}^{2}}\psi_{E2}\left(x\right))]=\\ =\int_{-\infty }^{0}dx[(1-{\left|\alpha \right|}^{2})|\psi_{E2}{|}^{2}+{\left|\alpha \right|}^{2}|\psi_{E1}{\left(x\right)|}^{2}+\left|\alpha \right|\sqrt{1-{\left|\alpha \right|}^{2}}\left(\psi_{E1}\psi_{E2}^{†}\left(x\right)+\psi_{E1}^{†}\psi_{E2}\left(x\right)\right)]=\\ =\int_{-\infty }^{0}dx[\left(1-cos{\left(\gamma \right)}^{2}\right)|\psi_{E2}{\left(x\right)|}^{2}+cos{\left(\gamma \right)}^{2}|\psi_{E1}\left(x\right){|}^{2}+sin\left(\gamma \right)cos\left(\gamma \right)\left(\psi_{E1}\left(x\right)\psi_{E2}^{†}\left(x\right)+\psi_{E1}{\left(x\right)}^{†}\psi_{E2}\left(x\right)\right)]. \end{array}$$

Since $S_{L}\left(\gamma \right)\left[\psi_{E1}\left(x\right),\psi_{E2}\left(x\right)\right]$ reaches a maximum with respect to γ, this implies $\frac{d}{d\gamma }S_{L}\left(\gamma \right)$$[\psi_{E1}\left(x\right),\psi_{E2}\left(x\right)]=0$.

(9) $$\begin{array}{c} 0=\int_{-\infty }^{0}dx[-2sin\left(\gamma \right)cos\left(\gamma \right)(|\psi_{E1}{\left(x\right)|}^{2}-|\psi_{E2}{\left(x\right)|}^{2})+\left(cos{\left(\gamma \right)}^{2}-sin{\left(\gamma \right)}^{2}\right)\left(\psi_{E1}\left(x\right)\psi_{E2}^{†}\left(x\right)+\psi_{E1}{\left(x\right)}^{†}\psi_{E2}\left(x\right)\right)]. \end{array}$$

which can be summarized as

(10) $$\begin{array}{c} 0=\int_{-\infty }^{0}dx[-sin\left(2\gamma \right)(|\psi_{E1}{\left(x\right)|}^{2}-|\psi_{E2}{\left(x\right)|}^{2})+\left(cos\left(2\gamma \right)\right)\left(\psi_{E1}\left(x\right)\psi_{E2}^{†}\left(x\right)+\psi_{E1}{\left(x\right)}^{†}\psi_{E2}\left(x\right)\right)]. \end{array}$$

and finally we have

(11) $$\begin{array}{c} \gamma =\frac{1}{2}ArcTan\left[\frac{\int_{-\infty }^{0}dx\left(\psi_{E1}\left(x\right)\psi_{E2}^{†}\left(x\right)+\psi_{E1}{\left(x\right)}^{†}\psi_{E2}\left(x\right)\right)}{\int_{-\infty }^{0}dx(|\psi_{E1}{\left(x\right)|}^{2}-|\psi_{E2}\left(x\right){|}^{2})}\right]=\frac{1}{2}ArcTan\left[r\right], \end{array}$$

where

(12) $$\begin{array}{c} r=\frac{\int_{-\infty }^{0}dx\left(\psi_{E1}\left(x\right)\psi_{E2}^{†}\left(x\right)+\psi_{E1}{\left(x\right)}^{†}\psi_{E2}\left(x\right)\right)}{\int_{-\infty }^{0}dx(|\psi_{E1}{\left(x\right)|}^{2}-|\psi_{E2}\left(x\right){|}^{2})}. \end{array}$$

Consequently, we have

$$\begin{array}{c} |\alpha |=cos(\frac{1}{2}ArcTan\left[\frac{\int_{-\infty }^{0}dx\left(\psi_{E1}\left(x\right)\psi_{E2}^{†}\left(x\right)+\psi_{E1}{\left(x\right)}^{†}\psi_{E2}\left(x\right)\right)}{\int_{-\infty }^{0}dx(|\psi_{E1}{\left(x\right)|}^{2}-|\psi_{E2}\left(x\right){|}^{2})}\right]),\\ |\beta |=sin(\frac{1}{2}ArcTan\left[\frac{\int_{-\infty }^{0}dx\left(\psi_{E1}\left(x\right)\psi_{E2}^{†}\left(x\right)+\psi_{E1}{\left(x\right)}^{†}\psi_{E2}\left(x\right)\right)}{\int_{-\infty }^{0}dx(|\psi_{E1}{\left(x\right)|}^{2}-|\psi_{E2}\left(x\right){|}^{2})}\right]), \end{array}$$

Finally, one can write

(13) $$\begin{array}{c} \left(\begin{array}{c} w_{L}\left(x\right)\\ w_{R}\left(x\right) \end{array}\right)=\\ \left(\begin{array}{cc} +cos(\frac{1}{2}ArcTan\left[\frac{\int_{-\infty }^{0}dx\left(\psi_{E1}\left(x\right)\psi_{E2}^{†}\left(x\right)+\psi_{E1}{\left(x\right)}^{†}\psi_{E2}\left(x\right)\right)}{\int_{-\infty }^{0}dx(|\psi_{E1}{\left(x\right)|}^{2}-|\psi_{E2}\left(x\right){|}^{2})}\right]) & sin(\frac{1}{2}ArcTan\left[\frac{\int_{-\infty }^{0}dx\left(\psi_{E1}\left(x\right)\psi_{E2}^{†}\left(x\right)+\psi_{E1}{\left(x\right)}^{†}\psi_{E2}\left(x\right)\right)}{\int_{-\infty }^{0}dx(|\psi_{E1}{\left(x\right)|}^{2}-|\psi_{E2}\left(x\right){|}^{2})}\right])\\ -sin(\frac{1}{2}ArcTan\left[\frac{\int_{-\infty }^{0}dx\left(\psi_{E1}\left(x\right)\psi_{E2}^{†}\left(x\right)+\psi_{E1}{\left(x\right)}^{†}\psi_{E2}\left(x\right)\right)}{\int_{-\infty }^{0}dx(|\psi_{E1}{\left(x\right)|}^{2}-|\psi_{E2}\left(x\right){|}^{2})}\right]) & cos(\frac{1}{2}ArcTan\left[\frac{\int_{-\infty }^{0}dx\left(\psi_{E1}\left(x\right)\psi_{E2}^{†}\left(x\right)+\psi_{E1}{\left(x\right)}^{†}\psi_{E2}\left(x\right)\right)}{\int_{-\infty }^{0}dx(|\psi_{E1}{\left(x\right)|}^{2}-|\psi_{E2}\left(x\right){|}^{2})}\right]) \end{array}\right)\left(\begin{array}{c} \psi_{E1}\left(x\right)\\ \psi_{E2}\left(x\right) \end{array}\right). \end{array}$$

Such reasoning can be conducted for any two different energy levels, as well as for N different energetic levels. If the quantum state is given as

(14) $$\begin{array}{c} |\psi \rangle =e^{\frac{E_{1}\left(t-t_{0}\right)}{i\hbar }}e^{i\gamma_{E1}}\sqrt{p_{E1}}|E_{1}\rangle +e^{\frac{E_{2}\left(t-t_{0}\right)}{i\hbar }}e^{i\gamma_{E2}}\sqrt{p_{E2}}|E_{2}\rangle \end{array}$$

then

(15) $$\begin{array}{c} \left(\begin{array}{c} \alpha \left(t\right)w_{L}\left(x\right)\\ \beta \left(t\right)w_{R}\left(x\right) \end{array}\right)=\left(\begin{array}{cc} +cos(\frac{1}{2}ArcTan\left(r\right)) & +sin(\frac{1}{2}ArcTan\left(r\right))\\ -sin(\frac{1}{2}ArcTan\left(r\right)) & +cos(\frac{1}{2}ArcTan\left(r\right)) \end{array}\right)\left(\begin{array}{c} e^{\frac{E_{1}\left(t-t_{0}\right)}{i\hbar }}e^{i\gamma_{E1}}\sqrt{p_{E1}}\psi_{E1}\left(x\right)\\ e^{\frac{E_{2}\left(t-t_{0}\right)}{i\hbar }}e^{i\gamma_{E2}}\sqrt{p_{E2}}\psi_{E2}\left(x\right) \end{array}\right). \end{array}$$

The last implies that

(16) $$\begin{array}{c} \alpha_{c}\left(t\right)=\int_{-\infty }^{+\infty }dx\left(\begin{array}{cc} {}[cos\left(\frac{1}{2}ArcTan\left(r\right)\right)\psi_{E1}^{†}\left(x\right), & sin\left(\frac{1}{2}ArcTan\left(r\right)\right)\psi_{E2}^{†}\left(x\right) \end{array}\right)\times \\ \left(\begin{array}{cc} +cos(\frac{1}{2}ArcTan\left(r\right)), & +sin(\frac{1}{2}ArcTan\left(r\right))\\ -sin(\frac{1}{2}ArcTan\left(r\right)), & +cos(\frac{1}{2}ArcTan\left(r\right)) \end{array}\right)\left(\begin{array}{c} e^{\frac{E_{1}\left(t-t_{0}\right)}{i\hbar }}e^{i\gamma_{E1}}\sqrt{p_{E1}}\psi_{E1}\left(x\right)\\ e^{\frac{E_{2}\left(t-t_{0}\right)}{i\hbar }}e^{i\gamma_{E2}}\sqrt{p_{E2}}\psi_{E2}\left(x\right) \end{array}\right)=\\ =\int_{-\infty }^{+\infty }dx\left(\begin{array}{cc} w_{1,1}^{*}\psi_{E1}^{†}\left(x\right), & w_{1,2}^{*}\psi_{E2}^{†}\left(x\right) \end{array}\right)\times \left(\begin{array}{cc} w_{1,1} & w_{1,2}\\ w_{2,1} & w_{2,2} \end{array}\right)\left(\begin{array}{c} e^{\frac{E_{1}\left(t-t_{0}\right)}{i\hbar }}e^{i\gamma_{E1}}\sqrt{p_{E1}}\psi_{E1}\left(x\right)\\ e^{\frac{E_{2}\left(t-t_{0}\right)}{i\hbar }}e^{i\gamma_{E2}}\sqrt{p_{E2}}\psi_{E2}\left(x\right) \end{array}\right)=\\ =\int_{-\infty }^{+\infty }dx\left(\begin{array}{cc} w_{1,1}^{*}\psi_{E1}^{†}\left(x\right), & w_{1,2}^{*}\psi_{E2}^{†}\left(x\right) \end{array}\right)\left(\begin{array}{c} w_{1,1}e^{\frac{E_{1}\left(t-t_{0}\right)}{i\hbar }}e^{i\gamma_{E1}}\sqrt{p_{E1}}\psi_{E1}\left(x\right)+w_{1,2}e^{\frac{E_{2}\left(t-t_{0}\right)}{i\hbar }}e^{i\gamma_{E2}}\sqrt{p_{E2}}\psi_{E2}\left(x\right)\\ w_{2,1}e^{\frac{E_{1}\left(t-t_{0}\right)}{i\hbar }}e^{i\gamma_{E1}}\sqrt{p_{E1}}\psi_{E1}\left(x\right)+w_{2,2}e^{\frac{E_{2}\left(t-t_{0}\right)}{i\hbar }}e^{i\gamma_{E2}}\sqrt{p_{E2}}\psi_{E2}\left(x\right) \end{array}\right)=\\ =w_{1,1}^{*}w_{1,1}e^{\frac{E_{1}\left(t-t_{0}\right)}{i\hbar }}e^{i\gamma_{E1}}\sqrt{p_{E1}}+w_{1,2}^{*}w_{2,2}e^{\frac{E_{2}\left(t-t_{0}\right)}{i\hbar }}e^{i\gamma_{E2}}\sqrt{p_{E2}}=\\ =cos{\left(\frac{1}{2}ArcTan\left(r\right)\right)}^{2}e^{\frac{E_{1}\left(t-t_{0}\right)}{i\hbar }}e^{i\gamma_{E1}}\sqrt{p_{E1}}+sin\left(\frac{1}{2}ArcTan\left(r\right)\right)cos\left(\frac{1}{2}ArcTan\left(r\right)\right)e^{\frac{E_{2}\left(t-t_{0}\right)}{i\hbar }}e^{i\gamma_{E2}}\sqrt{p_{E2}}=\\ =cos\left(\frac{1}{2}ArcTan\left(r\right)\right)\left[cos\left(2ArcTan\left(r\right)\right)e^{\frac{E_{1}\left(t-t_{0}\right)}{i\hbar }}e^{i\gamma_{E1}}\sqrt{p_{E1}}+sin\left(\frac{1}{2}ArcTan\left(r\right)\right)e^{\frac{E_{2}\left(t-t_{0}\right)}{i\hbar }}e^{i\gamma_{E2}}\sqrt{p_{E2}}\right]=\alpha \left(t\right), \end{array}$$

and

(17) $$\begin{array}{c} \beta_{c}\left(t\right)=\int_{-\infty }^{+\infty }dx\left(\begin{array}{cc} -sin\left(\frac{1}{2}ArcTan\left(r\right)\right)\psi_{E1}^{†}\left(x\right), & cos\left(\frac{1}{2}ArcTan\left(r\right)\right)\psi_{E2}^{†}\left(x\right) \end{array}\right)\times \\ \left(\begin{array}{cc} +cos(\frac{1}{2}ArcTan\left(r\right)), & +sin(\frac{1}{2}ArcTan\left(r\right))\\ -sin(\frac{1}{2}ArcTan\left(r\right)), & +cos(\frac{1}{2}ArcTan\left(r\right)) \end{array}\right)\left(\begin{array}{c} e^{\frac{E_{1}\left(t-t_{0}\right)}{i\hbar }}e^{i\gamma_{E1}}\sqrt{p_{E1}}\psi_{E1}\left(x\right)\\ e^{\frac{E_{2}\left(t-t_{0}\right)}{i\hbar }}e^{i\gamma_{E2}}\sqrt{p_{E2}}\psi_{E2}\left(x\right) \end{array}\right)=\\ =\int_{-\infty }^{+\infty }dx\left(\begin{array}{cc} w_{2,1}^{*}\psi_{E1}^{†}\left(x\right), & w_{2,2}^{*}\psi_{E2}^{†}\left(x\right) \end{array}\right)\times \left(\begin{array}{cc} w_{1,1} & w_{1,2}\\ w_{2,1} & w_{2,2} \end{array}\right)\left(\begin{array}{c} e^{\frac{E_{1}\left(t-t_{0}\right)}{i\hbar }}e^{i\gamma_{E1}}\sqrt{p_{E1}}\psi_{E1}\left(x\right)\\ e^{\frac{E_{2}\left(t-t_{0}\right)}{i\hbar }}e^{i\gamma_{E2}}\sqrt{p_{E2}}\psi_{E2}\left(x\right) \end{array}\right)=\\ =\int_{-\infty }^{+\infty }dx\left(\begin{array}{cc} w_{2,1}^{*}\psi_{E1}^{†}\left(x\right), & w_{2,2}^{*}\psi_{E2}^{†}\left(x\right) \end{array}\right)\left(\begin{array}{c} w_{1,1}e^{\frac{E_{1}\left(t-t_{0}\right)}{i\hbar }}e^{i\gamma_{E1}}\sqrt{p_{E1}}\psi_{E1}\left(x\right)+w_{1,2}e^{\frac{E_{2}\left(t-t_{0}\right)}{i\hbar }}e^{i\gamma_{E2}}\sqrt{p_{E2}}\psi_{E2}\left(x\right)\\ w_{2,1}e^{\frac{E_{1}\left(t-t_{0}\right)}{i\hbar }}e^{i\gamma_{E1}}\sqrt{p_{E1}}\psi_{E1}\left(x\right)+w_{2,2}e^{\frac{E_{2}\left(t-t_{0}\right)}{i\hbar }}e^{i\gamma_{E2}}\sqrt{p_{E2}}\psi_{E2}\left(x\right) \end{array}\right)=\\ =w_{2,1}^{*}w_{1,1}e^{\frac{E_{1}\left(t-t_{0}\right)}{i\hbar }}e^{i\gamma_{E1}}\sqrt{p_{E1}}+w_{2,2}^{*}w_{2,2}e^{\frac{E_{2}\left(t-t_{0}\right)}{i\hbar }}e^{i\gamma_{E2}}\sqrt{p_{E2}}=\\ =-sin\left(\frac{1}{2}ArcTan\left(r\right)\right)cos\left(\frac{1}{2}ArcTan\left(r\right)\right)e^{\frac{E_{1}\left(t-t_{0}\right)}{i\hbar }}e^{i\gamma_{E1}}\sqrt{p_{E1}}+cos{\left(\frac{1}{2}ArcTan\left(r\right)\right)}^{2}e^{\frac{E_{2}\left(t-t_{0}\right)}{i\hbar }}e^{i\gamma_{E2}}\sqrt{p_{E2}}=\\ =cos\left(\frac{1}{2}ArcTan\left(r\right)\right)\left[-sin\left(\frac{1}{2}ArcTan\left(r\right)\right)e^{\frac{E_{1}\left(t-t_{0}\right)}{i\hbar }}e^{i\gamma_{E1}}\sqrt{p_{E1}}+cos\left(\frac{1}{2}ArcTan\left(r\right)\right)e^{\frac{E_{2}\left(t-t_{0}\right)}{i\hbar }}e^{i\gamma_{E2}}\sqrt{p_{E2}}\right]=\beta \left(t\right). \end{array}$$

In this way, we can convert the quantum information represented by eigenergy qubits (as mostly used with formula $|\psi \rangle =\sqrt{p_{E1}}e^{i\gamma_{E1}}{|\psi \rangle }_{E1}+\sqrt{p_{E2}}e^{i\gamma_{E2}}{|\psi \rangle }_{E2}$) in position-based format $|\psi \rangle =\alpha \left(t\right){|w\rangle }_{1}+\beta \left(t\right){|w\rangle }_{2}$ (Wannier qubit format). We notice that

(18) $$\begin{array}{c} \frac{\alpha_{c}\left(t\right)}{\beta_{c}\left(t\right)}=\frac{\left[cos\left(\frac{1}{2}ArcTan\left(r\right)\right)e^{\frac{E_{1}\left(t-t_{0}\right)}{i\hbar }}e^{i\gamma_{E1}}\sqrt{p_{E1}}+sin\left(\frac{1}{2}ArcTan\left(r\right)\right)e^{\frac{E_{2}\left(t-t_{0}\right)}{i\hbar }}e^{i\gamma_{E2}}\sqrt{p_{E2}}\right]}{\left[-sin\left(\frac{1}{2}ArcTan\left(r\right)\right)e^{\frac{E_{1}\left(t-t_{0}\right)}{i\hbar }}e^{i\gamma_{E1}}\sqrt{p_{E1}}+cos\left(\frac{1}{2}ArcTan\left(r\right)\right)e^{\frac{E_{2}\left(t-t_{0}\right)}{i\hbar }}e^{i\gamma_{E2}}\sqrt{p_{E2}}\right]}=\\ =\frac{\left[cos\left(\frac{1}{2}ArcTan\left(r\right)\right)\sqrt{p_{E1}}+sin\left(\frac{1}{2}ArcTan\left(r\right)\right)e^{\frac{\left(E_{2}-E_{1}\right)\left(t-t_{0}\right)}{i\hbar }}e^{i(\gamma_{E2}-\gamma_{E1})}\sqrt{p_{E2}}\right]}{\left[-sin\left(\frac{1}{2}ArcTan\left(r\right)\right)\sqrt{p_{E1}}+cos\left(\frac{1}{2}ArcTan\left(r\right)\right)e^{\frac{\left(E_{2}-E_{1}\right)\left(t-t_{0}\right)}{i\hbar }}e^{i(\gamma_{E2}-\gamma_{E1})}\sqrt{p_{E2}}\right]}=\\ =\frac{\left[\frac{\sqrt{p_{E1}}}{\sqrt{p_{E2}}}+Tan\left(\frac{1}{2}ArcTan\left(r\right)\right)e^{-i\frac{\left(E_{2}-E_{1}\right)\left(t-t_{0}\right)}{\hbar }}e^{i(\gamma_{E2}-\gamma_{E1})}\right]}{\left[-Tan\left(\frac{1}{2}ArcTan\left(r\right)\right)\frac{\sqrt{p_{E1}}}{\sqrt{p_{E2}}}\right]+e^{-i\frac{\left(E_{2}-E_{1}\right)\left(t-t_{0}\right)}{\hbar }}e^{i(\gamma_{E2}-\gamma_{E1})}}, \end{array}$$

which implies that occupancy of the full Bloch sphere is not achievable by a single Wannier qubit in the static electric and magnetic fields. Still, we can approach arbitrarily close to the south and north poles of the Bloch sphere by regulating *r* (achieved from the different effective potential generated by biasing electrodes) and by setting the arbitrary ratio $\frac{\sqrt{p_{E1}}}{\sqrt{p_{E2}}}$. Furthermore, we immediately obtain

(19) $$\begin{array}{c} E_{p1}=\int_{-\infty }^{+\infty }dx\left[w_{L}^{*}\left(x\right)\hat{H}w_{L}\left(x\right)\right]=\int_{-\infty }^{+\infty }dx\left[\left(\alpha^{†}\psi_{E1}^{†}\left(x\right)+\beta^{†}\psi_{E2}^{†}\left(x\right)\right)\hat{H}\left(\alpha \psi_{E1}\left(x\right)+\beta \psi_{E2}\left(x\right)\right)\right]=\\ ={\left|\alpha \right|}^{2}E_{1}+{\left|\beta \right|}^{2}E_{2}={\left(1-|\beta \right|}^{2})E_{1}+{\left|\beta \right|}^{2}E_{2}=E_{1}+{\left|\beta \right|}^{2}\left(E_{2}-E_{1}\right)=\\ =E_{1}+\left(E_{2}-E_{1}\right){\left|sin\left(\frac{1}{2}ArcTan\left[\frac{\int_{-\infty }^{0}dx\left(\psi_{E1}\left(x\right)\psi_{E2}^{†}\left(x\right)+\psi_{E1}{\left(x\right)}^{†}\psi_{E2}\left(x\right)\right)}{\int_{-\infty }^{0}dx(|\psi_{E1}{\left(x\right)|}^{2}-|\psi_{E2}\left(x\right){|}^{2})}\right]\right)\right|}^{2}, \end{array}$$

(20) $$\begin{array}{c} E_{p2}=\int_{-\infty }^{+\infty }dx\left[w_{R}^{*}\left(x\right)\hat{H}w_{R}\left(x\right)\right]=\int_{-\infty }^{+\infty }dx\left(-\beta^{†}\psi_{E1}^{†}\left(x\right)+\alpha^{†}\psi_{E2}^{†}\left(x\right)\right)\times \\ \times \hat{H}\left(-\beta \psi_{E1}\left(x\right)+\alpha \psi_{E2}\left(x\right)\right)={\left|\beta \right|}^{2}E_{1}+{\left|\alpha \right|}^{2}E_{2}=E_{1}+\left(E_{2}-E_{1}\right){\left|\alpha \right|}^{2}=\\ =E_{1}+\left(E_{2}-E_{1}\right){\left|cos\left(\frac{1}{2}ArcTan\left[\frac{\int_{-\infty }^{0}dx\left(\psi_{E1}\left(x\right)\psi_{E2}^{†}\left(x\right)+\psi_{E1}{\left(x\right)}^{†}\psi_{E2}\left(x\right)\right)}{\int_{-\infty }^{0}dx(|\psi_{E1}{\left(x\right)|}^{2}-|\psi_{E2}\left(x\right){|}^{2})}\right]\right)\right|}^{2}, \end{array}$$

(21) $$\begin{array}{c} t_{s,2\to 1}=\int_{-\infty }^{+\infty }dx\left[w_{R}^{*}\left(x\right)\hat{H}w_{L}\left(x\right)\right]=\int_{-\infty }^{+\infty }dx\left(-\beta^{†}\psi_{E1}^{†}\left(x\right)+\alpha^{†}\psi_{E2}^{†}\left(x\right)\right)\times \\ \times \hat{H}\left(\alpha \psi_{E1}\left(x\right)+\beta \psi_{E2}\left(x\right)\right)=-\alpha \beta^{*}E_{1}+\alpha^{*}\beta E_{2}=\left(E_{2}-E_{1}\right)\alpha \beta =\\ =\frac{1}{2}sin\left(2\frac{\int_{-\infty }^{0}dx\left(\psi_{E1}\left(x\right)\psi_{E2}^{†}\left(x\right)+\psi_{E1}{\left(x\right)}^{†}\psi_{E2}\left(x\right)\right)}{\int_{-\infty }^{0}dx(|\psi_{E1}{\left(x\right)|}^{2}-|\psi_{E2}\left(x\right){|}^{2})}\right)\left(E_{2}-E_{1}\right)=\\ =\frac{\left(E_{2}-E_{1}\right)}{2}\frac{\frac{\int_{-\infty }^{0}dx\left(\psi_{E1}\left(x\right)\psi_{E2}^{†}\left(x\right)+\psi_{E1}{\left(x\right)}^{†}\psi_{E2}\left(x\right)\right)}{\int_{-\infty }^{0}dx(|\psi_{E1}{\left(x\right)|}^{2}-|\psi_{E2}\left(x\right){|}^{2})}}{\sqrt{1+{\left[\frac{\int_{-\infty }^{0}dx\left(\psi_{E1}\left(x\right)\psi_{E2}^{†}\left(x\right)+\psi_{E1}{\left(x\right)}^{†}\psi_{E2}\left(x\right)\right)}{\int_{-\infty }^{0}dx(|\psi_{E1}{\left(x\right)|}^{2}-|\psi_{E2}\left(x\right){|}^{2})}\right]}^{2}}}, \end{array}$$

(22) $$\begin{array}{c} t_{s,1\to 2}=\int_{-\infty }^{+\infty }dx\left[w_{L}^{*}\left(x\right)\hat{H}w_{R}\left(x\right)\right]=\int_{-\infty }^{+\infty }dx\left(\alpha^{†}\psi_{E1}^{†}\left(x\right)+\beta^{†}\psi_{E2}^{†}\left(x\right)\right)\times \\ \times \hat{H}\left(-\beta \psi_{E1}\left(x\right)+\alpha \psi_{E2}\left(x\right)\right)=-\alpha^{*}\beta E_{1}+\alpha \beta^{*}E_{2}=\left(E_{2}-E_{1}\right)\alpha \beta =\\ =\left(E_{2}-E_{1}\right)\frac{1}{2}2cos\left(\frac{1}{2}ArcTan\left(r\right)\right)sin\left(\frac{1}{2}ArcTan\left(r\right)\right)=\left(E_{2}-E_{1}\right)\frac{1}{2}sin\left(ArcTan\left(r\right)\right)=\\ =\left(E_{2}-E_{1}\right)\frac{1}{2}sin\left(ArcTan\left(r\right)\right)=\frac{\left(E_{2}-E_{1}\right)}{2}\frac{r}{\sqrt{1+r^{2}}}=\\ =\frac{\left(E_{2}-E_{1}\right)}{2}\frac{\frac{\int_{-\infty }^{0}dx\left(\psi_{E1}\left(x\right)\psi_{E2}^{†}\left(x\right)+\psi_{E1}{\left(x\right)}^{†}\psi_{E2}\left(x\right)\right)}{\int_{-\infty }^{0}dx(|\psi_{E1}{\left(x\right)|}^{2}-|\psi_{E2}\left(x\right){|}^{2})}}{\sqrt{1+{\left[\frac{\int_{-\infty }^{0}dx\left(\psi_{E1}\left(x\right)\psi_{E2}^{†}\left(x\right)+\psi_{E1}{\left(x\right)}^{†}\psi_{E2}\left(x\right)\right)}{\int_{-\infty }^{0}dx(|\psi_{E1}{\left(x\right)|}^{2}-|\psi_{E2}\left(x\right){|}^{2})}\right]}^{2}}} \end{array}$$

It was shown in this work that a tight-binding model can be fundamentally derived from Schrödinger formalism, and thus it is useful for the description of Wannier qubits (position-based qubits). The results can be summarized using the tight-binding model, as a function of eigenenergies of the Schrödinger Hamiltonian in the form of

(23) $$\begin{array}{c} \left(\begin{array}{cc} E_{p1} & t_{s21}\\ t_{s12} & E_{p2} \end{array}\right)=\left(\begin{array}{cc} E_{1}+{\left|sin\left(\frac{1}{2}ArcTan\left(r\right)\right)\right|}^{2}\left(E_{2}-E_{1}\right) & \left(E_{2}-E_{1}\right)\frac{1}{2}sin\left(ArcTan\left(r\right)\right)\\ \left(E_{2}-E_{1}\right)\frac{1}{2}sin\left(ArcTan\left(r\right)\right) & E_{1}+\left(E_{2}-E_{1}\right){\left|cos\left(\frac{1}{2}ArcTan\left(r\right)\right)\right|}^{2} \end{array}\right), \end{array}$$

with *r* given by (12) and using Formulas (19)–(21) based on Formula (2). We will prove Equation (2) using the Schrödinger equation, as given in the next section. If we assume the possible escape of an electron from a coupled quantum dot system, the wavefunction is no longer normalized to one and we can replace the real value eigenenergies with complex value energies, so $E_{1}\to E_{1r}+iE_{1i}$ and $E_{2}\to E_{2r}+iE_{2i}$. In such a case, the effective tight-binding model corresponding to complex value eigenenergies can be expressed as

(24) $$\begin{array}{c} \left(\begin{array}{cc} E_{p1D} & t_{s21D}\\ t_{s12D} & E_{p2D} \end{array}\right)=\left(\begin{array}{cc} E_{1r}+{\left|sin\left(\frac{1}{2}ArcTan\left(r\right)\right)\right|}^{2}\left(E_{2r}-E_{1r}\right) & \left(E_{2r}-E_{1r}\right)\frac{1}{2}sin\left(ArcTan\left(r\right)\right)\\ \left(E_{2r}-E_{1r}\right)\frac{1}{2}sin\left(ArcTan\left(r\right)\right) & E_{1r}+\left(E_{2r}-E_{1r}\right){\left|cos\left(\frac{1}{2}ArcTan\left(r\right)\right)\right|}^{2} \end{array}\right)+\\ +\sqrt{-1}\left(\begin{array}{cc} E_{1i}+{\left|sin\left(\frac{1}{2}ArcTan\left(r\right)\right)\right|}^{2}\left(E_{2i}-E_{1i}\right) & \left(E_{2i}-E_{1i}\right)\frac{1}{2}sin\left(ArcTan\left(r\right)\right)\\ \left(E_{2i}-E_{1i}\right)\frac{1}{2}sin\left(ArcTan\left(r\right)\right) & E_{1i}+\left(E_{2i}-E_{1i}\right){\left|cos\left(\frac{1}{2}ArcTan\left(r\right)\right)\right|}^{2} \end{array}\right). \end{array}$$

The dissipative version of the tight-binding model accounting for electron escape from a two-quantum dot system due to tunneling is non-Hermitian, while the non-dissipative version of the tight-binding model is Hermitian.

### 3.1. Equivalence of Wannier and Schrödinger Formalism

Let us start from the static case of electric and magnetic time-independent fields, so

(25) $$\begin{array}{c} \hat{H}|\psi (x,t)\rangle =\left(-\frac{\hbar^{2}}{2m}\frac{d^{2}}{dx^{2}}+V\left(x\right)\right)\left(\begin{array}{c} e^{i\gamma_{E1}\left(t\right)}\sqrt{p_{E1}}\psi_{E1}\left(x\right)\\ e^{i\gamma_{E2}\left(t\right)}\sqrt{p_{E2}}\psi_{E2}\left(x\right) \end{array}\right)=\left(\begin{array}{cc} E_{1} & 0\\ 0 & E_{2} \end{array}\right)\left(\begin{array}{c} e^{i\gamma_{E1}\left(t\right)}\sqrt{p_{E1}}\psi_{E1}\left(x\right)\\ e^{i\gamma_{E2}\left(t\right)}\sqrt{p_{E2}}\psi_{E2}\left(x\right) \end{array}\right)=\\ =i\hbar \frac{d}{dt}\left(\begin{array}{c} e^{i\gamma_{E1}\left(t\right)}\sqrt{p_{E1}}\psi_{E1}\left(x\right)\\ e^{i\gamma_{E2}\left(t\right)}\sqrt{p_{E2}}\psi_{E2}\left(x\right) \end{array}\right) \end{array}$$

which is equivalent to

(26) $$\begin{array}{c} \left(\begin{array}{cc} E_{1} & 0\\ 0 & E_{2} \end{array}\right)\left(\begin{array}{c} e^{i\gamma_{E1}\left(t\right)}\sqrt{p_{E1}}\\ e^{i\gamma_{E2}\left(t\right)}\sqrt{p_{E2}} \end{array}\right)=i\hbar \frac{d}{dt}\left(\begin{array}{c} e^{i\gamma_{E1}\left(t\right)}\sqrt{p_{E1}}\\ e^{i\gamma_{E2}\left(t\right)}\sqrt{p_{E2}} \end{array}\right), \end{array}$$

and we have

(27) $$\begin{array}{c} \left(\begin{array}{cc} w_{1,1} & w_{1,2}\\ w_{2,1} & w_{2,2} \end{array}\right)\left(\begin{array}{cc} E_{1} & 0\\ 0 & E_{2} \end{array}\right)\left(\begin{array}{c} e^{i\gamma_{E1}\left(t\right)}\sqrt{p_{E1}}\psi_{E1}\left(x\right)\\ e^{i\gamma_{E2}\left(t\right)}\sqrt{p_{E2}}\psi_{E2}\left(x\right) \end{array}\right)=i\hbar \frac{d}{dt}\left(\begin{array}{cc} w_{1,1} & w_{1,2}\\ w_{2,1} & w_{2,2} \end{array}\right)\left(\begin{array}{c} e^{i\gamma_{E1}\left(t\right)}\sqrt{p_{E1}}\psi_{E1}\left(x\right)\\ e^{i\gamma_{E2}\left(t\right)}\sqrt{p_{E2}}\psi_{E2}\left(x\right) \end{array}\right), \end{array}$$

which yields

(28) $$\begin{array}{c} \left(\begin{array}{cc} w_{1,1} & w_{1,2}\\ w_{2,1} & w_{2,2} \end{array}\right)\left(\begin{array}{cc} E_{1} & 0\\ 0 & E_{2} \end{array}\right)\left(\begin{array}{c} e^{i\gamma_{E1}\left(t\right)}\sqrt{p_{E1}}\psi_{E1}\left(x\right)\\ e^{i\gamma_{E2}\left(t\right)}\sqrt{p_{E2}}\psi_{E2}\left(x\right) \end{array}\right)=\\ =i\hbar \frac{d}{dt}\left(\begin{array}{cc} w_{1,1} & w_{1,2}\\ w_{2,1} & w_{2,2} \end{array}\right)\left(\begin{array}{c} e^{i\gamma_{E1}\left(t\right)}\sqrt{p_{E1}}w_{E1}\left(x\right)\\ e^{i\gamma_{E2}\left(t\right)}\sqrt{p_{E2}}w_{E2}\left(x\right) \end{array}\right) \end{array}$$

which implies

(29) $$\begin{array}{c} \left(\begin{array}{cc} w_{1,1} & w_{1,2}\\ w_{2,1} & w_{2,2} \end{array}\right)\left(\begin{array}{cc} E_{1} & 0\\ 0 & E_{2} \end{array}\right){\left(\begin{array}{cc} w_{1,1} & w_{1,2}\\ w_{2,1} & w_{2,2} \end{array}\right)}^{-1}\left(\begin{array}{cc} w_{1,1} & w_{1,2}\\ w_{2,1} & w_{2,2} \end{array}\right)\left(\begin{array}{c} e^{i\gamma_{E1}\left(t\right)}\sqrt{p_{E1}}\psi_{E1}\left(x\right)\\ e^{i\gamma_{E2}\left(t\right)}\sqrt{p_{E2}}\psi_{E2}\left(x\right) \end{array}\right)=i\hbar \frac{d}{dt}\left(\begin{array}{cc} w_{1,1} & w_{1,2}\\ w_{2,1} & w_{2,2} \end{array}\right)\left(\begin{array}{c} e^{i\gamma_{E1}\left(t\right)}\sqrt{p_{E1}}w_{E1}\left(x\right)\\ e^{i\gamma_{E2}\left(t\right)}\sqrt{p_{E2}}w_{E2}\left(x\right) \end{array}\right). \end{array}$$

From

(30) $$\begin{array}{c} \alpha_{c}\left(t\right)\left|w_{L}\left(x\right)>+\beta_{c}\left(t\right)\right|w_{R}\left(x\right)>=\left(\begin{array}{c} \alpha_{c}\left(t\right)w_{L}\left(x\right)\\ \beta_{c}\left(t\right)w_{R}\left(x\right) \end{array}\right)=\left(\begin{array}{cc} w_{1,1} & w_{1,2}\\ w_{2,1} & w_{2,2} \end{array}\right)\left(\begin{array}{c} e^{i\gamma_{E1}\left(t\right)}\sqrt{p_{E1}}w_{E1}\left(x\right)\\ e^{i\gamma_{E2}\left(t\right)}\sqrt{p_{E2}}w_{E2}\left(x\right) \end{array}\right) \end{array}$$

we obtain

(31) $$\begin{array}{c} \left[\left(\begin{array}{cc} w_{1,1} & w_{1,2}\\ w_{2,1} & w_{2,2} \end{array}\right)\left(\begin{array}{cc} E_{1} & 0\\ 0 & E_{2} \end{array}\right)\left(\begin{array}{cc} w_{2,2} & -w_{1,2}\\ -w_{2,1} & w_{1,1} \end{array}\right)\right]\left(\begin{array}{c} \alpha_{c}\left(t\right)w_{L}\left(x\right)\\ \beta_{c}\left(t\right)w_{R}\left(x\right) \end{array}\right)=i\hbar \frac{d}{dt}\left(\begin{array}{c} \alpha_{c}\left(t\right)w_{L}\left(x\right)\\ \beta_{c}\left(t\right)w_{R}\left(x\right) \end{array}\right) \end{array}$$

which gives

(32) $$\begin{array}{c} \left[\left(\begin{array}{cc} w_{1,1}w_{2,2}E_{1}-w_{1,2}w_{2,1}E_{2} & -w_{1,1}w_{1,2}E_{1}+w_{1,2}w_{1,1}E_{2}\\ w_{2,1}w_{2,2}E_{1}-w_{2,1}w_{2,2}E_{2} & -w_{1,2}w_{2,1}E_{1}+w_{1,1}w_{2,2}E_{2} \end{array}\right)\right]\left(\begin{array}{c} \alpha_{c}\left(t\right)w_{L}\left(x\right)\\ \beta_{c}\left(t\right)w_{R}\left(x\right) \end{array}\right)=i\hbar \frac{d}{dt}\left(\begin{array}{c} \alpha_{c}\left(t\right)w_{L}\left(x\right)\\ \beta_{c}\left(t\right)w_{R}\left(x\right) \end{array}\right) \end{array}$$

The previous formula can be rewritten as

(33) $$\begin{array}{c} \left(\begin{array}{cc} \int_{-\infty }^{+\infty }dxw_{L}^{*}\left(x\right)\left(-\frac{\hbar^{2}}{2m}+V\left(x\right)\right)w_{L}\left(x\right) & \int_{-\infty }^{+\infty }dxw_{L}^{*}\left(x\right)\left(-\frac{\hbar^{2}}{2m}+V\left(x\right)\right)w_{R}\left(x\right)\\ \int_{-\infty }^{+\infty }dxw_{R}^{*}\left(x\right)\left(-\frac{\hbar^{2}}{2m}+V\left(x\right)\right)w_{L}\left(x\right) & \int_{-\infty }^{+\infty }dxw_{R}^{*}\left(x\right)\left(-\frac{\hbar^{2}}{2m}+V\left(x\right)\right)w_{R}\left(x\right) \end{array}\right)\left(\begin{array}{c} \alpha_{c}\left(t\right)\\ \beta_{c}\left(t\right) \end{array}\right)=i\hbar \frac{d}{dt}\left(\begin{array}{c} \alpha_{c}\left(t\right)\\ \beta_{c}\left(t\right) \end{array}\right) \end{array}$$

or equivalently

(34) $$\begin{array}{c} \left(\begin{array}{cc} E_{p1} & t_{s}\\ t_{s}^{*} & E_{p2} \end{array}\right)\left(\begin{array}{c} \alpha_{c}\left(t\right)w_{L}\left(x\right)\\ \beta_{c}\left(t\right)w_{R}\left(x\right) \end{array}\right)=\\ \left(\begin{array}{cc} \int_{-\infty }^{+\infty }dxw_{L}^{*}\left(x\right)\left(-\frac{\hbar^{2}}{2m}+V\left(x\right)\right)w_{L}\left(x\right) & \int_{-\infty }^{+\infty }dxw_{L}^{*}\left(x\right)\left(-\frac{\hbar^{2}}{2m}+V\left(x\right)\right)w_{R}\left(x\right)\\ \int_{-\infty }^{+\infty }dxw_{R}^{*}\left(x\right)\left(-\frac{\hbar^{2}}{2m}+V\left(x\right)\right)w_{L}\left(x\right) & \int_{-\infty }^{+\infty }dxw_{R}^{*}\left(x\right)\left(-\frac{\hbar^{2}}{2m}+V\left(x\right)\right)w_{R}\left(x\right) \end{array}\right)\left(\begin{array}{c} \alpha_{c}\left(t\right)w_{L}\left(x\right)\\ \beta_{c}\left(t\right)w_{R}\left(x\right) \end{array}\right)=i\hbar \frac{d}{dt}\left(\begin{array}{c} \alpha_{c}\left(t\right)w_{L}\left(x\right)\\ \beta_{c}\left(t\right)w_{R}\left(x\right) \end{array}\right). \end{array}$$

Therefore, we can always obtain a tight-binding model from a Schrödinger model with two eigenergies.

### 3.2. Rabi Oscillations in the Tight-Binding Model

In the time-dependent case, we can consider the existence of Rabi oscillations by assuming the effective Hamiltonian to be of the form $H=E_{1}|E_{1}\rangle \langle E_{1}|+E_{2}|E_{2}\rangle \langle E_{2}|+f_{1}\left(t\right)e^{i\xi (t)}|E_{2}\rangle \langle E_{1}|+f_{1}\left(t\right)e^{-i\xi (t)}|E_{1}\rangle \langle E_{2}|$ with $f\left(t\right)=f_{1}\left(t\right)e^{i\xi (t)}$ acting on the q-state given by $|\psi \rangle =\sqrt{p_{E1}}e^{i\gamma_{1}}|E_{1}\rangle +\sqrt{p_{E2}}e^{i\gamma_{2}}|E_{2}\rangle$. Immediately, we obtain

(35) $$\begin{array}{ccc} \sqrt{p_{E1}}E_{1}+f_{1}\sqrt{p_{E2}}e^{i(\gamma_{2}-\gamma_{1})}e^{i\xi } & = & \hbar [i\frac{1}{2\sqrt{p_{E1}}}\frac{d}{dt}p_{E1}-\sqrt{p_{E1}}\frac{d}{dt}\gamma_{1}], \end{array}$$

(36) $$\begin{array}{ccc} f_{1}\sqrt{p_{E1}}e^{-i(\gamma_{2}-\gamma_{1})}e^{-i\xi }+\sqrt{p_{E2}}E_{2} & = & \hbar [i\frac{1}{2\sqrt{p_{E2}}}\frac{d}{dt}p_{E2}-\sqrt{p_{E2}}\frac{d}{dt}\gamma_{2}]. \end{array}$$

Consequently, we have $2f_{1}\sqrt{p_{E1}}\sqrt{p_{E2}}sin\left[\left(\gamma_{2}-\gamma_{1}\right)+\xi \right]=\hbar \frac{d}{dt}p_{E1}=-\hbar \frac{d}{dt}p_{E2}$ and by setting parametrization ${\left[sin\left(\Theta \left(t\right)\right)\right]}^{2}=p_{E1}\left(t\right),{\left[cos\left(\Theta \left(t\right)\right)\right]}^{2}=p_{E2}\left(t\right)$ we obtain

(37) $$\begin{array}{ccc} f_{1}\left(t\right)sin\left[\left(\gamma_{2}\left(t\right)-\gamma_{1}\left(t\right)\right)+\xi \left(t\right)\right] & = & \hbar \frac{d}{dt}\Theta \left(t\right). \end{array}$$

Furthermore, using $\sqrt{p_{E1}}E_{1}+f_{1}\sqrt{p_{E2}}cos\left[\left(\gamma_{2}-\gamma_{1}\right)+\xi \right]=-\hbar \sqrt{p_{E1}}\frac{d}{dt}\gamma_{1}$ we obtain

(38) $$\begin{array}{c} E_{1}+f_{1}\left(t\right)ctan\left(\Theta \left(t\right)\right)cos\left[\left(\gamma_{2}\left(t\right)-\gamma_{1}\left(t\right)\right)+\xi \left(t\right)\right]=-\hbar \frac{d}{dt}\gamma_{1}\left(t\right),\\ E_{2}+f_{1}\left(t\right)tan\left(\Theta \left(t\right)\right)cos\left[\left(\gamma_{2}\left(t\right)-\gamma_{1}\left(t\right)\right)+\xi \left(t\right)\right]=-\hbar \frac{d}{dt}\gamma_{2}\left(t\right),\\ -\frac{\left(E_{2}-E_{1}\right)\left(t-t_{0}\right)}{\hbar }+\frac{1}{\hbar }\int_{t_{0}}^{t}dt^{\prime }f_{1}\left(t^{\prime }\right)cos\left[\left(\gamma_{2}\left(t^{\prime }\right)-\gamma_{1}\left(t^{\prime }\right)\right)+\xi \left(t^{\prime }\right)\right]\left(ctan\left(\Theta \left(t^{\prime }\right)\right)-tan\left(\Theta \left(t^{\prime }\right)\right)\right)+\left(\gamma_{2}\left(t_{0}\right)-\gamma_{1}\left(t_{0}\right)\right)\\ =\gamma_{2}\left(t\right)-\gamma_{1}\left(t\right). \end{array}$$

By setting $\left(\gamma_{2}\left(t\right)-\gamma_{1}\left(t\right)\right)+\xi \left(t\right)=-\frac{\left(E_{2}-E_{1}\right)\left(t-t_{0}\right)}{\hbar }+\left(\gamma_{2}\left(t_{0}\right)-\gamma_{1}\left(t_{0}\right)\right)+\xi \left(t\right)=\frac{\pi }{2}$ we obtain a constant speed of change $\gamma_{2}\left(t\right)-\gamma_{1}\left(t\right)$ with time as well as a constant value of $\hbar \frac{d}{dt}\Theta \left(t\right)$ under the condition that $f_{1}\left(t\right)$ is constant with time. Otherwise, the situation is not analytical and we need to deal with a system of three coupled ordinary differential equations given as

(39) $$\begin{array}{ccc} E_{1}+f_{1}\left(t\right)ctan\left(\Theta \left(t\right)\right)cos\left[\left(\gamma_{2}\left(t\right)-\gamma_{1}\left(t\right)\right)+\xi \left(t\right)\right] & = & -\hbar \frac{d}{dt}\gamma_{1}\left(t\right), \end{array}$$

(40) $$\begin{array}{ccc} E_{2}+f_{1}\left(t\right)tan\left(\Theta \left(t\right)\right)cos\left[\left(\gamma_{2}\left(t\right)-\gamma_{1}\left(t\right)\right)+\xi \left(t\right)\right] & = & -\hbar \frac{d}{dt}\gamma_{2}\left(t\right), \end{array}$$

(41) $$\begin{array}{ccc} f_{1}\left(t\right)sin\left[\left(\gamma_{2}\left(t\right)-\gamma_{1}\left(t\right)\right)+\xi \left(t\right)\right] & = & +\hbar \frac{d}{dt}\Theta \left(t\right), \end{array}$$

where the given parametric real value functions are ξ(t) and $f_{1}\left(t\right)$. In the time-dependent case, we can consider the existence of Rabi oscillations by assuming the effective Hamiltonian to be of the form $H=E_{1}|E_{1}\rangle \langle E_{1}|+E_{2}|E_{2}\rangle \langle E_{2}|+f_{1}\left(t\right)e^{i\xi (t)}|E_{2}\rangle \langle E_{1}|+f_{1}\left(t\right)e^{-i\xi (t)}|E_{1}\rangle$$\langle E_{2}|$ with $f\left(t\right)=f_{1}\left(t\right)e^{i\xi (t)}$, where $f_{1}\left(t\right)\in R$ and $\xi \left(t\right)=\frac{(E_{2}-E_{1})t}{\hbar }+\rho =\frac{\left(E_{2}-E_{1}\right)\left(t-t_{0}\right)}{\hbar }-\left(\gamma_{2}\left(t_{0}\right)-\gamma_{1}\left(t_{0}\right)\right)+\frac{\pi }{2}$ and with $\rho =-\left(\gamma_{2}\left(t_{0}\right)-\gamma_{1}\left(t_{0}\right)\right)+\frac{\pi }{2}$, so for certain classes of V(x,t) potential we can write

(42) $$\begin{array}{c} \hat{H}|\psi \rangle =\left(-\frac{\hbar^{2}}{2m}\frac{d^{2}}{dx^{2}}+V\left(x,t\right)\right)\left(\begin{array}{c} e^{i\gamma_{E1}\left(t\right)}\sqrt{p_{E1}\left(t\right)}\psi_{E1}\left(x\right)\\ e^{i\gamma_{E2}\left(t\right)}\sqrt{p_{E2}\left(t\right)}\psi_{E2}\left(x\right) \end{array}\right)=\left(\begin{array}{cc} E_{1} & f(t)\\ f{\left(t\right)}^{*} & E_{2} \end{array}\right)\left(\begin{array}{c} e^{i\gamma_{E1}\left(t\right)}\sqrt{p_{E1}}\psi_{E1}\left(x\right)\\ e^{i\gamma_{E2}\left(t\right)}\sqrt{p_{E2}}\psi_{E2}\left(x\right) \end{array}\right)=\\ =i\hbar \frac{d}{dt}\left(\begin{array}{c} e^{i\gamma_{E1}\left(t\right)}\sqrt{p_{E1}\left(t\right)}\psi_{E1}\left(x\right)\\ e^{i\gamma_{E2}\left(t\right)}\sqrt{p_{E2}\left(t\right)}\psi_{E2}\left(x\right) \end{array}\right), \end{array}$$

which is equivalent to

(43) $$\begin{array}{c} \left(\begin{array}{cc} E_{1} & f(t)\\ f{\left(t\right)}^{*} & E_{2} \end{array}\right)\left(\begin{array}{c} e^{i\gamma_{E1}\left(t\right)}\sqrt{p_{E1}\left(t\right)}\\ e^{i\gamma_{E2}\left(t\right)}\sqrt{p_{E2}\left(t\right)} \end{array}\right)=i\hbar \frac{d}{dt}\left(\begin{array}{c} e^{i\gamma_{E1}\left(t\right)}\sqrt{p_{E1}\left(t\right)}\\ e^{i\gamma_{E2}\left(t\right)}\sqrt{p_{E2}\left(t\right)} \end{array}\right) \end{array}$$

with

(44) $$\begin{array}{c} \left[\left(\begin{array}{cc} w_{1,1} & w_{1,2}\\ w_{2,1} & w_{2,2} \end{array}\right)\left(\begin{array}{cc} E_{1} & f(t)\\ f{\left(t\right)}^{*} & E_{2} \end{array}\right)\left(\begin{array}{cc} w_{2,2} & -w_{1,2}\\ -w_{2,1} & w_{1,1} \end{array}\right)\right]\left[\left(\begin{array}{cc} w_{1,1} & w_{1,2}\\ w_{2,1} & w_{2,2} \end{array}\right)\left(\begin{array}{c} e^{i\gamma_{E1}\left(t\right)}\sqrt{p_{E1}\left(t\right)}\\ e^{i\gamma_{E2}\left(t\right)}\sqrt{p_{E2}\left(t\right)} \end{array}\right)\right]=\\ =i\hbar \frac{d}{dt}\left[\left(\begin{array}{cc} w_{1,1} & w_{1,2}\\ w_{2,1} & w_{2,2} \end{array}\right)\left(\begin{array}{c} e^{i\gamma_{E1}\left(t\right)}\sqrt{p_{E1}\left(t\right)}\\ e^{i\gamma_{E2}\left(t\right)}\sqrt{p_{E2}\left(t\right)} \end{array}\right)\right], \end{array}$$

which implies

(45) $$\begin{array}{c} \left(\begin{array}{cc} w_{1,1}w_{2,2}E_{1}-f\left(t\right)w_{2,1}w_{1,1}+w_{1,2}w_{2,2}f{\left(t\right)}^{*}-w_{1,2}w_{2,1}E_{2} & -w_{1,2}w_{1,1}E_{1}+f\left(t\right)w_{1,1}w_{1,1}-w_{1,2}w_{1,2}f^{*}\left(t\right)+w_{1,1}w_{1,2}E_{2}\\ w_{2,2}w_{2,2}f{\left(t\right)}^{*}-w_{2,2}w_{2,1}E_{2}+w_{2,1}w_{2,2}E_{1}-f\left(t\right)w_{2,1}w_{2,1} & -w_{2,1}w_{1,2}E_{1}+w_{2,1}f\left(t\right)w_{1,1}-w_{2,2}w_{1,2}f{\left(t\right)}^{*}+w_{2,2}w_{1,1}E_{2} \end{array}\right)\left(\begin{array}{c} \alpha_{q}\left(t\right)\\ \beta_{q}\left(t\right) \end{array}\right)\\ =i\hbar \frac{d}{dt}\left(\begin{array}{c} \alpha_{q}\left(t\right)\\ \beta_{q}\left(t\right) \end{array}\right), \end{array}$$

which can be written as

(46) $$\begin{array}{c} \left(\begin{array}{cc} E_{p1}\left(t\right) & t_{s(1,2)}\left(t\right)\\ t_{s(2,1)}\left(t\right) & E_{p2}\left(t\right) \end{array}\right)\left(\begin{array}{c} \alpha_{q}\left(t\right)\\ \beta_{q}\left(t\right) \end{array}\right)=\left(\begin{array}{cc} a_{1,1}\left(t\right) & a_{1,2}\left(t\right)\\ a_{2,1}\left(t\right) & a_{2,2}\left(t\right) \end{array}\right)\left(\begin{array}{c} \alpha_{q}\left(t\right)\\ \beta_{q}\left(t\right) \end{array}\right)=i\hbar \frac{d}{dt}\left(\begin{array}{c} \alpha_{q}\left(t\right)\\ \beta_{q}\left(t\right) \end{array}\right), \end{array}$$

with four time-dependent coefficients:

(47) $$\begin{array}{c} a_{1,1}=cos{\left(\frac{1}{2}ArcTan\left(r\right)\right)}^{2}E_{1}+\left(f\left(t\right)+f{\left(t\right)}^{*}\right)\left(sin\left(\frac{1}{2}ArcTan\left(r\right)\right)cos\left(\frac{1}{2}ArcTan\left(r\right)\right)\right)+E_{2}sin{\left(\frac{1}{2}ArcTan\left(r\right)\right)}^{2}=\\ =cos{\left(\frac{1}{2}ArcTan\left(r\right)\right)}^{2}E_{1}+cos\left(\frac{(E_{2}-E_{1})t}{\hbar }+\rho \right)f_{1}\left(t\right)sin(ArcTan\left(r\right)+E_{2}sin{\left(\frac{1}{2}ArcTan\left(r\right)\right)}^{2}, \end{array}$$

(48) $$\begin{array}{c} a_{2,1}=cos{\left(\frac{1}{2}ArcTan\left(r\right)\right)}^{2}f{\left(t\right)}^{*}+\left(E_{2}-E_{1}\right)\left(sin\left(\frac{1}{2}ArcTan\left(r\right)\right)cos\left(\frac{1}{2}ArcTan\left(r\right)\right)\right)-f\left(t\right)sin{\left(\frac{1}{2}ArcTan\left(r\right)\right)}^{2}=\\ =cos{\left(\frac{1}{2}ArcTan\left(r\right)\right)}^{2}2cos\left(\frac{(E_{2}-E_{1})t}{\hbar }+\rho \right)f_{1}\left(t\right)+\frac{1}{2}\left(E_{2}-E_{1}\right)sin\left(\frac{1}{2}ArcTan\left(r\right)\right)\\ -f_{1}\left(t\right)\left[cos\left(\frac{(E_{2}-E_{1})t}{\hbar }+\rho \right)\right]+if_{1}\left(t\right)\left[sin\left(\frac{(E_{2}-E_{1})t}{\hbar }+\rho \right)\right], \end{array}$$

(49) $$\begin{array}{c} a_{1,2}=sin\left(\frac{1}{2}ArcTan\left(r\right)\right)cos\left(\frac{1}{2}ArcTan\left(r\right)\right)\left(E_{2}-E_{1}\right)+f\left(t\right){\left(cos\left(\frac{1}{2}ArcTan\left(r\right)\right)\right)}^{2}-{\left(sin\left(\frac{1}{2}ArcTan\left(r\right)\right)\right)}^{2}f^{*}\left(t\right)=\\ =\frac{1}{2}\left(E_{2}-E_{1}\right)sin\left(\frac{1}{2}ArcTan\left(r\right)\right)+cos\left(\frac{(E_{2}-E_{1})t}{\hbar }+\rho \right)f_{1}\left(t\right)\left[{\left(cos\left(\frac{1}{2}ArcTan\left(r\right)\right)\right)}^{2}-1\right]-if_{1}\left(t\right)sin\left(\frac{(E_{2}-E_{1})t}{\hbar }+\rho \right), \end{array}$$

(50) $$\begin{array}{c} a_{2,2}=sin{\left(\frac{1}{2}ArcTan\left(r\right)\right)}^{2}E_{1}-\left[f\left(t\right)+f{\left(t\right)}^{*}\right]cos\left(\frac{1}{2}ArcTan\left(r\right)\right)sin\left(\frac{1}{2}ArcTan\left(r\right)\right)+E_{2}cos{\left(\frac{1}{2}ArcTan\left(r\right)\right)}^{2}=\\ =sin{\left(\frac{1}{2}ArcTan\left(r\right)\right)}^{2}E_{1}-cos\left(\frac{(E_{2}-E_{1})t}{\hbar }+\rho \right)f_{1}\left(t\right)sin\left(ArcTan\left(r\right)\right)+E_{2}cos{\left(\frac{1}{2}ArcTan\left(r\right)\right)}^{2} \end{array}$$

which gives $f\left(t\right)+f^{*}\left(t\right)=2cos\left(\frac{(E_{2}-E_{1})t}{\hbar }+\rho \right)f_{1}\left(t\right)$. We can always use

(51) $$\begin{array}{c} a\left(t\right)f\left(t\right)-b\left(t\right)f{\left(t\right)}^{*}=a\left(t\right)f\left(t\right)-\left[-a+\left(b+a\right)\right]f{\left(t\right)}^{*}=a\left(t\right)\left[f\left(t\right)+f{\left(t\right)}^{*}\right]-\left(b+a\right)f{\left(t\right)}^{*}. \end{array}$$

The obtained Equation (47) for tight-binding model coefficients can be generalized by using $(\gamma_{2}\left(t\right)-\gamma_{1}\left(t\right))+\xi \left(t\right)$ obtained from (39)–(41) instead of expression $\frac{\left(E_{2}-E_{1}\right)\left(t-t_{0}\right)}{\hbar }-\left(\gamma_{2}\left(t_{0}\right)-\gamma_{1}\left(t_{0}\right)\right)+\frac{\pi }{2}$. We can incorporate the dissipation in the tight-binding model accompanied with Rabi oscillation and we obtain the coeffcients

(52) $$\begin{array}{c} a_{(1,1)D}=cos{\left(\frac{1}{2}ArcTan\left(r\right)\right)}^{2}\left(E_{1r}+iE_{1i}\right)+\left(f\left(t\right)+f{\left(t\right)}^{*}\right)\left(sin\left(\frac{1}{2}ArcTan\left(r\right)\right)cos\left(\frac{1}{2}ArcTan\left(r\right)\right)\right)+\left(E_{2r}+iE_{2i}\right)sin{\left(\frac{1}{2}ArcTan\left(r\right)\right)}^{2}\\ =cos{\left(\frac{1}{2}ArcTan\left(r\right)\right)}^{2}\left(E_{1r}+iE_{1i}\right)+cos\left(\frac{(E_{2r}+iE_{2i}-E_{1r}-iE_{1i})t}{\hbar }+\rho \right)f_{1}\left(t\right)sin(ArcTan\left(r\right)+\left(E_{2r}+iE_{2i}\right)sin{\left(\frac{1}{2}ArcTan\left(r\right)\right)}^{2}, \end{array}$$

(53) $$\begin{array}{c} a_{(2,1)D}=cos{\left(\frac{1}{2}ArcTan\left(r\right)\right)}^{2}f{\left(t\right)}^{*}+\left(E_{2r}-E_{1r}+i\left(E_{2i}-E_{1i}\right)\right)\left(sin\left(\frac{1}{2}ArcTan\left(r\right)\right)cos\left(\frac{1}{2}ArcTan\left(r\right)\right)\right)-f\left(t\right)sin{\left(\frac{1}{2}ArcTan\left(r\right)\right)}^{2}=\\ =cos{\left(\frac{1}{2}ArcTan\left(r\right)\right)}^{2}2cos\left(\frac{(E_{2r}-E_{1r})t}{\hbar }+i\frac{(E_{2i}-E_{1i})t}{\hbar }+\rho \right)f_{1}\left(t\right)+\frac{1}{2}\left(E_{2r}-E_{1r}\right)sin\left(\frac{1}{2}ArcTan\left(r\right)\right)+\\ +i\frac{1}{2}\left(E_{2i}-E_{1i}\right)sin\left(\frac{1}{2}ArcTan\left(r\right)\right)\\ -f_{1}\left(t\right)\left[cos\left(\frac{(E_{2r}-E_{1r})t}{\hbar }+i\frac{(E_{2i}-E_{1i})t}{\hbar }+\rho \right)\right]+if_{1}\left(t\right)\left[sin\left(\frac{(E_{2r}-E_{1r})t}{\hbar }+i\frac{(E_{2i}-E_{1i})t}{\hbar }+\rho \right)\right], \end{array}$$

(54) $$\begin{array}{c} a_{(1,2)D}=sin\left(\frac{1}{2}ArcTan\left(r\right)\right)cos\left(\frac{1}{2}ArcTan\left(r\right)\right)\left(E_{2r}-E_{1r}\right)+isin\left(\frac{1}{2}ArcTan\left(r\right)\right)cos\left(\frac{1}{2}ArcTan\left(r\right)\right)\left(E_{2i}-E_{1i}\right)+\\ +f\left(t\right){\left(cos\left(\frac{1}{2}ArcTan\left(r\right)\right)\right)}^{2}-{\left(sin\left(\frac{1}{2}ArcTan\left(r\right)\right)\right)}^{2}f^{*}\left(t\right)=\\ =\frac{1}{2}\left(\left(E_{2r}-E_{1r}\right)+i\left(E_{2i}-E_{1i}\right)\right)sin\left(\frac{1}{2}ArcTan\left(r\right)\right)+cos\left(\frac{(E_{2r}-E_{1r})t}{\hbar }+i\frac{(E_{2i}-E_{1i})t}{\hbar }+\rho \right)f_{1}\left(t\right)\left[{\left(cos\left(\frac{1}{2}ArcTan\left(r\right)\right)\right)}^{2}-1\right]\\ -if_{1}\left(t\right)sin\left(\frac{(E_{2r}-E_{1r})t}{\hbar }+i\frac{(E_{2i}-E_{1i})t}{\hbar }+\rho \right), \end{array}$$

(55) $$\begin{array}{c} a_{(2,2)D}=sin{\left(\frac{1}{2}ArcTan\left(r\right)\right)}^{2}\left(E_{1r}+iE_{1i}\right)-\left[f\left(t\right)+f{\left(t\right)}^{*}\right]cos\left(\frac{1}{2}ArcTan\left(r\right)\right)sin\left(\frac{1}{2}ArcTan\left(r\right)\right)+\left(E_{2r}+iE_{2i}\right)cos{\left(\frac{1}{2}ArcTan\left(r\right)\right)}^{2}=\\ sin{\left(\frac{1}{2}ArcTan\left(r\right)\right)}^{2}\left(E_{1r}+iE_{1i}\right)-cos\left(\frac{(E_{2r}-E_{1r})t}{\hbar }+i\frac{(E_{2i}-E_{1i})t}{\hbar }+\rho \right)f_{1}\left(t\right)sin\left(ArcTan\left(r\right)\right)+\left(E_{2r}+iE_{2i}\right)cos{\left(\frac{1}{2}ArcTan\left(r\right)\right)}^{2}, \end{array}$$

which imply the non-Hermicity of the dissipative tight-binding model with Rabi oscillations.

## 4. Open Curvy Loops Confining a Single Electron in Cartesian Coordinates in Schrödinger Formalism

### 4.1. Case of Deformed Curvy Wannier Qubit

Let us now go beyond the approach describing two straight interacting single-electron lines [23,26,27]. Let us consider a set of open curvy quasi-one-dimensional loops (that can be straight or curved smooth semiconductor nanowires with single electron), as described by x(s),y(s) and z(s), where *s* is the distance from the beginning to the end of the loop. The Schrödinger equation describing wave-packet movement in curvy nanowire is

(56) $$\begin{array}{c} \left[-\frac{\hbar^{2}}{2m}\left(\frac{d^{2}}{dx^{2}}+\frac{d^{2}}{dy^{2}}+\frac{d^{2}}{dz^{2}}\right)+V\left(x,y,z\right)\right]\psi \left(x,y,z\right)=E\psi \left(x,y,z\right). \end{array}$$

In such a case, the wave-packet moves in the way depicted in Figure 3. Moving along the curvy cable trajectory brings the relation $\frac{d}{dx}=\frac{ds}{dx}\frac{d}{ds}$ and similarly with *y* and *z*. We thus have $\frac{d^{2}}{dx^{2}}=\frac{ds}{dx}\frac{d}{ds}\left(\frac{ds}{dx}\frac{d}{ds}\right)={\left(\frac{1}{x^{\prime }\left(s\right)}\right)}^{2}\frac{d^{2}}{ds^{2}}-\left[\frac{x^{\prime \prime }\left(s\right)}{{\left(x^{\prime }\left(s\right)\right)}^{3}}\right]\frac{d}{ds}$. Thus, we obtain an equation in the form of

(57) $$\begin{array}{c} \left[-\frac{\hbar^{2}}{2m}\left[(\left[{\left(\frac{1}{x^{\prime }\left(s\right)}\right)}^{2}+{\left(\frac{1}{y^{\prime }\left(s\right)}\right)}^{2}+{\left(\frac{1}{z^{\prime }\left(s\right)}\right)}^{2}\right]\frac{d^{2}}{ds^{2}}-\left[\frac{x^{\prime \prime }\left(s\right)}{{\left(x^{\prime }\left(s\right)\right)}^{3}}+\frac{y^{\prime \prime }\left(s\right)}{{\left(y^{\prime }\left(s\right)\right)}^{3}}+\frac{z^{\prime \prime }\left(s\right)}{{\left(z^{\prime }\left(s\right)\right)}^{3}}\right]\frac{d}{ds})\right]+V\left(s\right)\right]\psi \left(s\right)=E\psi \left(s\right) \end{array}$$

which, with parametrization s=x,x(x)=x, can be summarized as

(58) $$\begin{array}{c} \left[-\frac{\hbar^{2}}{2m}\left[(\left[\left(1+{\left(\frac{1}{y^{\prime }\left(x\right)}\right)}^{2}+{\left(\frac{1}{z^{\prime }\left(x\right)}\right)}^{2}\right]\frac{d^{2}}{dx^{2}}-\left[+\frac{y^{\prime \prime }\left(x\right)}{{\left(y^{\prime }\left(x\right)\right)}^{3}}+\frac{z^{\prime \prime }\left(x\right)}{{\left(z^{\prime }\left(x\right)\right)}^{3}}\right]\frac{d}{dx}\right)\right]+V\left(x\right)\right]\psi \left(x\right)=E\psi \left(x\right), \end{array}$$

So we have

(59) $$\begin{array}{c} \left[-\frac{\hbar^{2}}{2m}\left[(f\left(s\right)\frac{d^{2}}{ds^{2}}-g\left(s\right)\frac{d}{ds})\right]+V\left(s\right)\right]\psi \left(s\right)=E\psi \left(s\right), \end{array}$$

where $f\left(s\right)=[{\left(\frac{1}{x^{\prime }\left(s\right)}\right)}^{2}+{\left(\frac{1}{y^{\prime }\left(s\right)}\right)}^{2}+{\left(\frac{1}{z^{\prime }\left(s\right)}\right)}^{2}]$, $g\left(s\right)=\left[\frac{x^{\prime \prime }\left(s\right)}{{\left(x^{\prime }\left(s\right)\right)}^{3}}+\frac{y^{\prime \prime }\left(s\right)}{{\left(y^{\prime }\left(s\right)\right)}^{3}}+\frac{z^{\prime \prime }\left(s\right)}{{\left(z^{\prime }\left(s\right)\right)}^{3}}\right]$. Similar reasoning with transformation from one coordinate system into another coordinate system is used in [28]. A more detailed derivation of the equation of motion for open-loop curved nanocable in cylindrical spherical coordinates is given by [25]. The most prominent feature that can be observed from the transformation from (56)–(59) is the occurrence of a dissipation term that is proportional to the operator $\frac{d}{ds}$, which is analogical to friction force (usually proportional to particle momentum). Indeed, the wave-packet travelling in a semiconductor nanowire is bent, which corresponds to occurrence of force changing the direction of wave-packet momentum. However, from another perspective we can say that by bending the straight trajectory of the particle (flat space) we can generate dissipation. Conversely, we can say that the type of dissipation in a given system with given coordinates can be changed by moving the frame of reference to a space with another curvature, so dissipation is reduced or cancelled. The fact of having dissipation-like phenomena in a quantum system will imply the fact of non-Hermicity of the Hamiltonian matrix, which will further imply the existence of a complex value for eigenergies. Interestingly, we can observe a dissipation-like term in the classical description of an electron moving in a nanowire, which is given by Equations (120) and (121).

Using Cartesian coordinates, we can formulate the following Schrödinger equation of motion:

(60) $$\begin{array}{c} -\frac{\hbar^{2}}{2m}\left[\left(1+\frac{1}{\left(\frac{d}{dx}y{\left(x\right)}^{2}\right)}\right)\frac{d^{2}}{dx^{2}}-\frac{\frac{d^{2}}{dx^{2}}y\left(x\right)}{{\left(\frac{d}{dx}y\left(x\right)\right)}^{2}}\frac{d}{dx}\right]\psi \left(x,y\left(x\right)\right)+V\left(x,y\left(x\right)\right)\psi \left(x,y\left(x\right)\right)=E\psi \left(x,y\left(x\right)\right). \end{array}$$

Local confining potential is given by V(x,y(x)) and can simply take into account the existence of 1, 2, 3 or more quantum dots across a semiconductor nanowire or can omit the existence of quantum dots and external polarizing electric and magnetic fields by being constant. Effectively, we have obtained a modified quasi-one-dimensional Schrödinger equation of the form

(61) $$\begin{array}{c} -\frac{\hbar^{2}}{2m}\left[\left(1+\frac{1}{\left(\frac{d}{dx}y{\left(x\right)}^{2}\right)}\right)\frac{d^{2}}{dx^{2}}-\frac{\frac{d^{2}}{dx^{2}}y\left(x\right)}{{\left(\frac{d}{dx}y\left(x\right)\right)}^{2}}\frac{d}{dx}\right]\psi \left(x\right)+V\left(x\right)\psi \left(x\right)=E\psi \left(x\right). \end{array}$$

**Figure 5:**
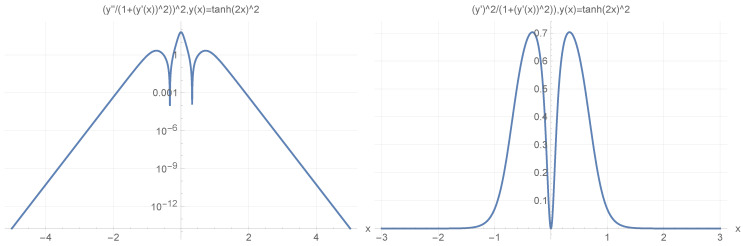

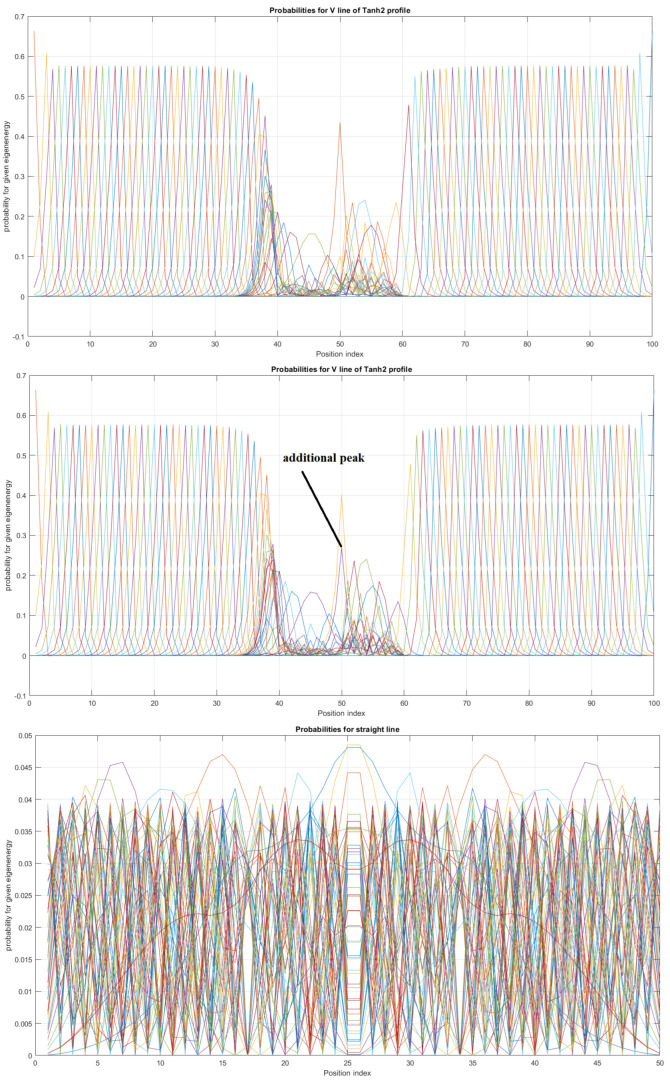
(**Upper Page**) Case of bent position-based qubit from Figure 1 with shape incorporated in functions dependence $\frac{y^{\prime \prime }\left(x\right)}{1+{\left(y^{\prime }\left(x\right)\right)}^{2}}$ (**left**) and of $\frac{y^{\prime }\left(x\right)}{1+{\left(y^{\prime }\left(x\right)\right)}^{2}}$ (**right**) for $y\left(x\right)={\left(tanh\left(2x\right)\right)}^{2}$. Visualization of cable shapes is based on examples from Figure 3 and Figure 4, and is constituting the emergence of an effective potential barrier during cable bending. This will lead to the methodology of description of position-based qubit generalization given by Figure 15. (**Current Page**) Probability distributions corresponding to eigenenergy wavefunctions for Tanh square nanowire (α=10) with three built−in q wells (**UPPER**), no q wells (**MIDDLE**) and for straight nanowire with two built−in q wells (**LOWER**).

**Figure 6:**
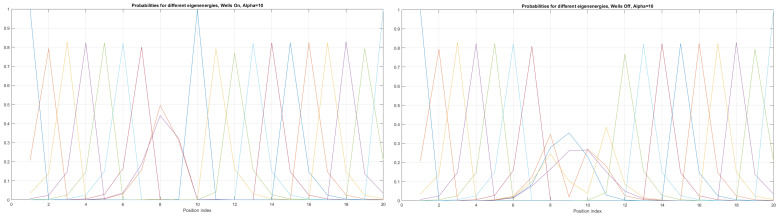
Detailed analysis of probability distributions (for first 20 eigenenergy modes) for Tanh square nanowires in the case of presence [**LEFT**]/no presence [**RIGHT**] of 3 q-wells with α=10. The lack of built-in q-wells results in a gap in q-state presence in the middle of the curved nanowire.

**Figure 7:**
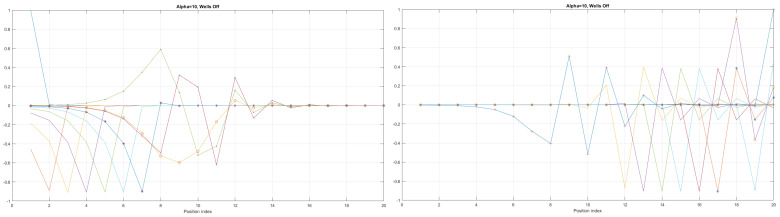
Detailed analysis of eigenenergy wavefunctions (for the first 20 eigenenergy modes) and with no built−in q−wells for Tanh square nanowires (with α=10), showing that the wave-functions are strongly localized due the non-zero curvature of the nanowire (equivalent to the condition that $\frac{\frac{d^{2}}{dx^{2}}}{y}\left(x\right){\left(\frac{d}{dx}y\left(x\right)\right)}^{2}\neq 0$).

**Figure 8:**
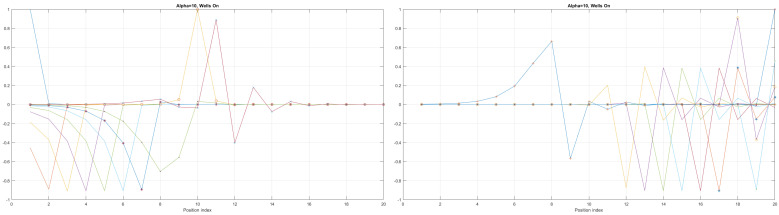
Detailed analysis of eigenenergy wavefunctions (for the first 20 eigenenergy modes) and with three built−in q−wells for Tanh square nanowires (with α=10), showing that the wave-functions are strongly localized due to the non-zero curvature of the nanowire (equivalent to the condition that $\frac{\frac{d^{2}}{dx^{2}}}{y}\left(x\right){\left(\frac{d}{dx}y\left(x\right)\right)}^{2}\neq 0$). Notice the difference in quantum wave-function behaviours compared to the case with no built−in q−wells shown in Figure 7.

**Figure 9:**
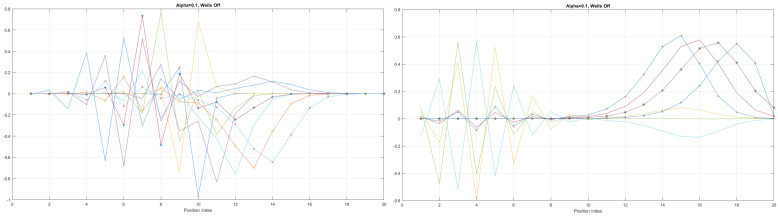
The case of coefficient α=0.1 reveals interesting eigenenergy wavefunction distributions for a cable with no built−in q−wells.

**Figure 10:**
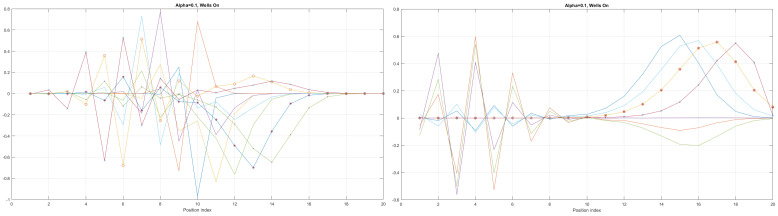
The case of coefficient α=0.1 reveals interesting eigenenergy wavefunction distitributions for a nanowire cable with three built−in q−wells.

**Figure 11:**
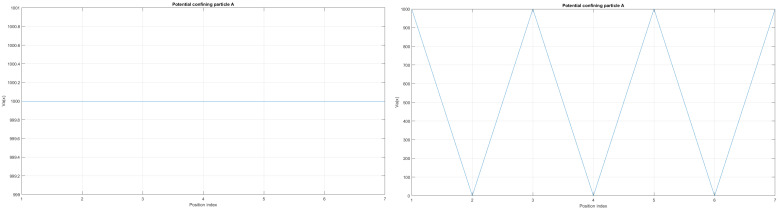
The case of local confining potentials as $V_{a}$ and $V_{b}$ for Tanh Square interacting nanowire cables without and with built-in q-wells.

**Figure 12:**
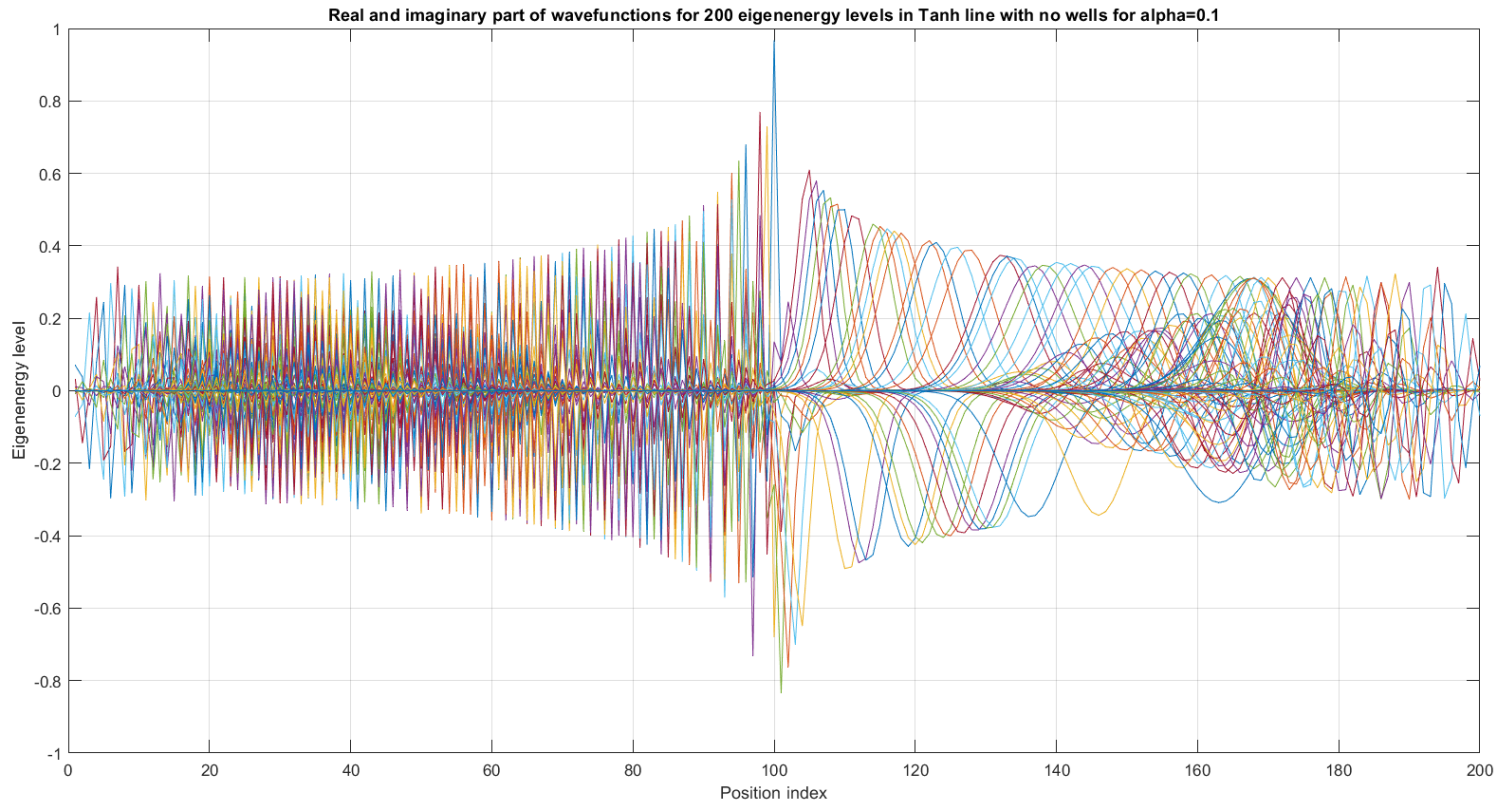
First 200 eigenenergy wavefunctions for Tanh Square V shape nanowire with no built-in quantum wells for α=0.1.

**Figure 13:**
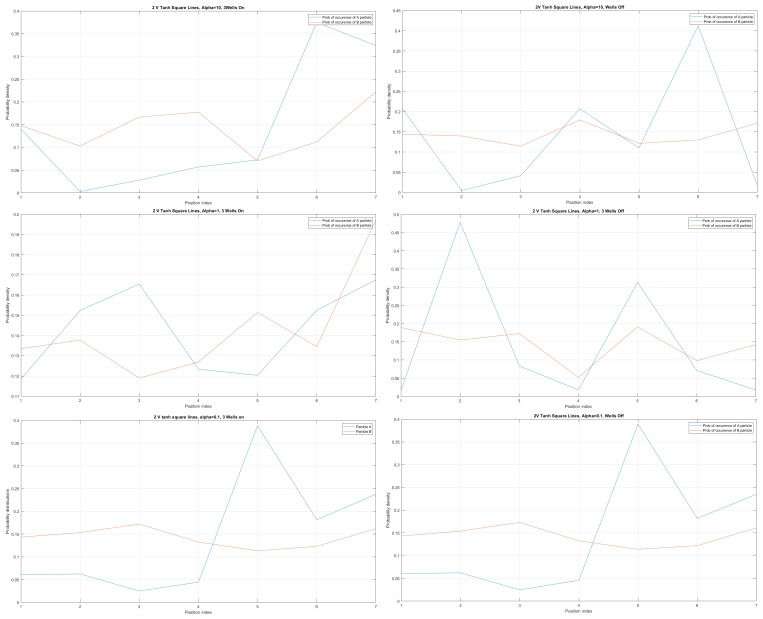
Case of two V Tanh Square Lines interacting and probability distributions around each line for electrons A and B with α= (10, 1, 0.1) for (**UPPER**, **MIDDLE**, **LOWER**) pictures with three quantum wells built-in [**LEFT**] and no quantum wells built-in [**RIGHT**].

Here, the shape of an open-loop nanowire is encoded in y(x) function dependence (reflected in the functions measuring cable curvature as by $\left(\frac{d}{dx}y\left(x\right)\right)$ and by $(\frac{d^{2}}{dx^{2}}y\left(x\right)$). The last **CM-Schrödinger** equation (**C**urvature **M**odified Schrödinger equation) can easily be generalized to an open-loop nanowire in three dimensions, in the form of a quasi-one-dimensional CM-Schrödinger equation as well. The results for a Tanh square nanocable are given in Figure 5, Figure 6, Figure 7, Figure 8, Figure 9, Figure 10, Figure 11, Figure 12 and Figure 13. Figure shows the case with a gap in the presence of quantum states due to nanowire cable bending. This effect is similar to the case of barrier existence due to nanocable bending, as expressed by a large α=10 coefficient. Furthermore, we can recognize that there is a relatively very weak (but still noticeable) effect of the existence of local confining potential. The lack of built-in q-wells may result in a gap in q-state presence in the middle of a curved nanowire, as depicted in Figure 6.

We can now present a detailed analysis of each eigenenergy wavefunction in the case of Tanh square nanowires (with α=10). We separate the first 10 of the 20 eigenenergy modes and the last 10 modes with no built-in q wells. The analysis shows that the wave-functions are strongly localized, due to the fact that the nanowire has non-zero curvature (equivalent to the condition that $\frac{\frac{d^{2}}{dx^{2}}}{y}\left(x\right){\left(\frac{d}{dx}y\left(x\right)\right)}^{2}\neq 0$), as depicted in Figure 7. Now, we conduct the same analysis but for three built-in q wells in Tanh square nanowires (with α=10). We obtain the wavefunction distribution depicted in Figure 8. Setting α=0.1 and with the same detailed analysis of eigenenergy wavefunctions (for the first 20 eigenenergy modes), we obtain two very similar probability distributions (in contrast with the case from Figure 8) for a cable with three built-in q-wells [LEFT] and no built-in q-wells [RIGHT], as depicted in Figure 9. Figure 10 shows the same detailed analysis of eigenenergy wavefunction distribution in a nanowire with coefficient α=0.1, but with three built-in q wells. The effective potential distribution for three built-in and no built-in q-wells is depicted in Figure 11.

The first 200 eigenenergy wavefunctions for Tanh Square V shape nanowire with no quantum wells for α=0.1 are depicted in Figure 12. The main conclusion is that bending nanowire leads to the separation of its two reservoirs, which means that there is no need to use a separate material between the two areas of the nanowire. This simplifies the technological process.

### 4.2. Derivation of Geometric Aharonov–Bohm Effect

#### Case of Ginzburg–Landau and Schrödinger Equations in Curved Space with Zero Vector Potential

Let us assume the existence of a curved quasi-one-dimensional semiconductor nanowire parametrized by (x(s),y(s),z(s)) and given by the Schrödinger equation

(62) $$\frac{-\hbar^{2}}{2m}\left[\frac{d^{2}}{dx^{2}}+\frac{d^{2}}{dy^{2}}+\frac{d^{2}}{dz^{2}}\right]\psi \left(x,y,z\right)+V\left(x,y,z\right)\psi \left(x,y,z\right)=E\psi \left(x,y,z\right),$$

or the existence of a quasi-one-dimensional superconducting nanowire represented by the Ginzburg–Landau (GL) equation, which is a mathematically non-linear version of Schrödinger equation obtained when we replace (V(x,y,z)−E)ψ(x,y,z) with $(\alpha \left(x,y,z\right)+\beta \left(x,y,z\right)|\psi \left(x,y,z\right){|}^{2})\psi \left(x,y,z\right)$. The GL equation is thus

(63) $$\frac{-\hbar^{2}}{2m}\left[\frac{d^{2}}{dx^{2}}+\frac{d^{2}}{dy^{2}}+\frac{d^{2}}{dz^{2}}\right]\psi \left(x,y,z\right)+\alpha \left(x,y,z\right)\psi \left(x,y,z\right)+\beta \left(x,y,z\right){\left|\psi \left(x,y,z\right)\right|}^{2}\psi \left(x,y,z\right)=0.$$

In both cases, the kinetic term can be simplified with use of the identity $\frac{d}{dx}=\frac{ds}{dx}\frac{d}{ds}$, which implies $\frac{d^{2}}{dx^{2}}={\left[\frac{1}{x^{\prime }\left(s\right)}\frac{d}{ds}\right]}^{2}=\frac{1}{{\left(x^{\prime }\left(s\right)\right)}^{2}}\frac{d^{2}}{ds^{2}}-\frac{x^{\prime \prime }\left(s\right)}{{\left(x^{\prime }\left(s\right)\right)}^{2}}\frac{d}{ds}$. This results in the equation

(64) $$\begin{array}{c} -\frac{\hbar^{2}}{2m}\left[\left(\frac{1}{{\left(x^{\prime }\left(s\right)\right)}^{2}}+\frac{1}{{\left(y^{\prime }\left(s\right)\right)}^{2}}+\frac{1}{{\left(z^{\prime }\left(s\right)\right)}^{2}}\right)\frac{d^{2}}{ds^{2}}-\left(\frac{x^{\prime \prime }\left(s\right)}{{\left(x^{\prime }\left(s\right)\right)}^{2}}+\frac{y^{\prime \prime }\left(s\right)}{{\left(y^{\prime }\left(s\right)\right)}^{2}}+\frac{z^{\prime \prime }\left(s\right)}{{\left(z^{\prime }\left(s\right)\right)}^{2}}\right)\frac{d}{ds}\right]\psi \left(s\right)+[\alpha \left(s\right)+\beta \left(s\right)|\psi \left(s\right){|}^{2}]\psi \left(s\right)=0. \end{array}$$

which can be written as

(65) $$\begin{array}{c} -\frac{\hbar^{2}}{2m}\left[f\left(s\right)\frac{d^{2}}{ds^{2}}-g\left(s\right)\frac{d}{ds}\right]{\psi \left(s\right)+[\alpha \left(s\right)+\beta \left(s\right)|\psi \left(s\right)|}^{2}]\psi \left(s\right)=0. \end{array}$$

with the two functions already introduced expressing nanowire curvature in three dimensions given in the form of

(66) $$\begin{array}{c} f\left(s\right)=\left(\frac{1}{{\left(x^{\prime }\left(s\right)\right)}^{2}}+\frac{1}{{\left(y^{\prime }\left(s\right)\right)}^{2}}+\frac{1}{{\left(z^{\prime }\left(s\right)\right)}^{2}}\right),g\left(s\right)=\left(\frac{x^{\prime \prime }\left(s\right)}{{\left(x^{\prime }\left(s\right)\right)}^{2}}+\frac{y^{\prime \prime }\left(s\right)}{{\left(y^{\prime }\left(s\right)\right)}^{2}}+\frac{z^{\prime \prime }\left(s\right)}{{\left(z^{\prime }\left(s\right)\right)}^{2}}\right). \end{array}$$

We obtain the following simplified version of the GL equation:

(67) $$\begin{array}{c} -\frac{\hbar^{2}}{2m}\left[\frac{d^{2}}{ds^{2}}-\frac{g(s)}{f(s)}\frac{d}{ds}\right]\psi \left(s\right)+[\frac{\alpha (s)}{f(s)}+\frac{\beta (s)}{f(s)}|\psi \left(s\right){|}^{2}]\psi \left(s\right)=0. \end{array}$$

which can be treated as a renormalization of the GL equation, so one has

(68) $$\begin{array}{c} -\frac{\hbar^{2}}{2m}\left[\frac{d^{2}}{ds^{2}}-g_{1}\left(s\right)\frac{d}{ds}\right]\psi \left(s\right)+[\alpha_{1}\left(s\right)+\beta {\left(s\right)}_{1}{\left|\psi \left(s\right)\right|}^{2}]\psi \left(s\right)=0. \end{array}$$

The previous expression clearly expresses similarity with the Schrödinger equation in one dimension with non-zero vector potential, so we can inspect this possibility by introducing the operator ${\left[\frac{d}{ds}-a_{1}\left(s\right)\right]}^{2}$ analogically to the canonical momentum square and introduce the u(s) function. In such a way, we represent the previous equation, but with new functions $a_{1}\left(s\right)$ and u(s), so

(69) $$\begin{array}{c} {\left(i\hbar \right)}^{2}\frac{1}{2m}{\left[\frac{d}{ds}-a_{1}\left(s\right)\right]}^{2}\psi \left(s\right)+u\left(s\right)\psi \left(s\right)+[\alpha_{1}\left(s\right)+\beta {\left(s\right)}_{1}{\left|\psi \left(s\right)\right|}^{2}]\psi \left(s\right)=0. \end{array}$$

Equivalently, we can obtain a different form

(70) $$\begin{array}{c} \frac{1}{2m}{\left[i\hbar \frac{d}{ds}-ia_{1}\left(s\right)\hbar \right]}^{2}\psi \left(s\right)+u\left(s\right)\psi \left(s\right)+[\alpha_{1}\left(s\right)+\beta {\left(s\right)}_{1}{\left|\psi \left(s\right)\right|}^{2}]\psi \left(s\right)=0. \end{array}$$

which finally reduces to the one-dimensional case of a Schrödinger equation with non-zero imaginary potential proportional to $ia_{1}\left(s\right)$ and with renormalized potential.

(71) $$\begin{array}{c} \frac{1}{2m}{\left[\frac{\hbar }{i}\frac{d}{ds}+ia_{1}\left(s\right)\hbar \right]}^{2}\psi \left(s\right)+u\left(s\right)\psi \left(s\right)+[\alpha_{1}\left(s\right)+\beta {\left(s\right)}_{1}{\left|\psi \left(s\right)\right|}^{2}]\psi \left(s\right)=0. \end{array}$$

which can be rewritten in the form

(72) $$\begin{array}{c} \frac{1}{2m}{\left[\frac{\hbar }{i}\frac{d}{ds}+ia_{1}\left(s\right)\hbar \right]}^{2}=\frac{1}{2m}\left[-\hbar^{2}\frac{d^{2}}{ds^{2}}-a_{1}{\left(s\right)}^{2}\hbar^{2}+\hbar^{2}2a_{1}\left(s\right)\frac{d}{ds}+\hbar^{2}\left(\frac{d}{ds}a_{1}\left(s\right)\right)\right]=\frac{1}{2m}\left[-\hbar^{2}\frac{d^{2}}{ds^{2}}+\hbar^{2}2a_{1}\left(s\right)\frac{d}{ds}\right]-u\left(s\right). \end{array}$$

which implies $u\left(s\right)=\frac{1}{2m}\left[-\left(\frac{d}{ds}a_{1}\left(s\right)\right)+a_{1}{\left(s\right)}^{2}\right]\hbar^{2}$. After comparison with Equation (68), we obtain $a_{1}\left(s\right)=\frac{1}{2}g_{1}\left(s\right)$ and thus we have GL cable curvature directly incorporated into the imaginary vector potential and renormalized α and β fields obtaining a curved GL equation in the form

(73) $$\begin{array}{c} \frac{1}{2m}{\left[\frac{\hbar }{i}\frac{d}{ds}+\frac{1}{2}ig_{1}\left(s\right)\hbar \right]}^{2}\psi \left(s\right)+\left[\frac{1}{4m}\left[-\hbar^{2}\left(\frac{d}{ds}g_{1}\left(s\right)\right)+\hbar^{2}{\left(\frac{1}{2}g_{1}\left(s\right)\right)}^{2}\right]+\alpha_{1}\left(s\right)\right]{\psi \left(s\right)+[\beta \left(s\right)}_{1}{\left|\psi \left(s\right)\right|}^{2}]\psi \left(s\right)=0. \end{array}$$

The **Curved GL equation** can be expressed by curvature functions f(s) and g(s) in the form

(74) $$\begin{array}{c} \frac{1}{2m}{\left[\frac{\hbar }{i}\frac{d}{ds}+\frac{1}{2}i\frac{g(s)}{f(s)}\hbar \right]}^{2}\psi \left(s\right)+\left[\frac{1}{4m}\left[-\hbar^{2}\left(\frac{d}{ds}\frac{g(s)}{f(s)}\right)+\hbar^{2}{\left(\frac{1}{2}\frac{g(s)}{f(s)}\right)}^{2}\right]+\frac{\alpha_{(}s)}{f(s)}\right]\psi \left(s\right)+[\frac{\beta (s)}{f(s)}|\psi \left(s\right){|}^{2}]\psi \left(s\right)=0. \end{array}$$

and the **Curved Schrödinger** equation can be expressed as

(75) $$\begin{array}{c} \frac{1}{2m}{\left[\frac{\hbar }{i}\frac{d}{ds}+\frac{1}{2}i\frac{g(s)}{f(s)}\hbar \right]}^{2}\psi \left(s\right)+\left[\frac{1}{4m}\left[-\hbar^{2}\left(\frac{d}{ds}\frac{g(s)}{f(s)}\right)+\hbar^{2}{\left(\frac{1}{2}\frac{g(s)}{f(s)}\right)}^{2}\right]+\frac{V(s)-E}{f(s)}+E_{b}\right]\psi \left(s\right)=E_{b}\psi \left(s\right), \end{array}$$

where the value of the renormalized Schrödinger potential from V(s) to $V{\left(s\right)}_{eff}$ in the quasi-one-dimensional description is

(76) $$\begin{array}{c} V\left(s\right)\to V_{eff}\left(s\right)=\left[\frac{1}{4m}\left[-\hbar^{2}\left(\frac{d}{ds}\frac{g(s)}{f(s)}\right)+\hbar^{2}{\left(\frac{1}{2}\frac{g(s)}{f(s)}\right)}^{2}\right]+\frac{V(s)-E}{f(s)}+E_{b}\right],\\ \frac{1}{2m}{\left[\frac{\hbar }{i}\frac{d}{ds}+\frac{1}{2}i\frac{g(s)}{f(s)}\hbar \right]}^{2}\psi \left(s\right)+V_{eff}\left(s\right)\psi \left(s\right)=E_{b}\psi \left(s\right). \end{array}$$

Here, we encounter a complex value of the momentum square ${\left[\frac{\hbar }{i}\frac{d}{ds}+\frac{1}{2}i\frac{g(s)}{f(s)}\hbar \right]}^{2}$, which indicates ongoing dissipation. We also notice the correspondence of vector potential with the field $\frac{g(s)}{f(s)}$, which can be denoted as $\frac{2e}{c}A\to i\frac{-1}{2}\frac{g(s)}{f(s)}\hbar$. We also obtained new values for the α and β fields in Ginzburg–Landau formalism given as

(77) $$\alpha \left(s\right)\to \left[\frac{1}{4m}\left[-\hbar^{2}\left(\frac{d}{ds}\frac{g(s)}{f(s)}\right)+\hbar^{2}{\left(\frac{1}{2}\frac{g(s)}{f(s)}\right)}^{2}\right]+\frac{\alpha_{(}s)}{f(s)}\right],\beta \left(s\right)\to \frac{\beta (s)}{f(s)}.$$

In the same fashion, we obtained new effective potential in Schrödinger formalism. The **Curved GL or Curved Schrödinger** equation leads to the new wave-function $\psi_{1}\left(s\right)$ given by a formula similar to the Aharonov–Bohm effect, but this time with imaginary phase imprint and expressed in three dimensions as

(78) $$\begin{array}{c} \psi_{1}\left(s\right)=\psi \left(s\right)exp\left[\frac{i}{\hbar }\int_{s_{1}}^{s}ds_{2}i\frac{1}{2}\frac{g(s_{2})}{f(s_{2})}\hbar \right]=\psi \left(s\right)exp\left[-\int_{s_{1}}^{s}ds_{2}\frac{g(s_{2})}{f(s_{2})}\right]=\psi \left(s\right)exp\left[-\int_{s_{1}}^{s}ds_{2}\frac{\frac{x^{\prime \prime }\left(s_{2}\right)}{{\left(x^{\prime }\left(s_{2}\right)\right)}^{2}}+\frac{y^{\prime \prime }\left(s_{2}\right)}{{\left(y^{\prime }\left(s_{2}\right)\right)}^{2}}+\frac{z^{\prime \prime }\left(s_{2}\right)}{{\left(z^{\prime }\left(s_{2}\right)\right)}^{2}}}{\left(\frac{1}{{\left(x^{\prime }\left(s_{2}\right)\right)}^{2}}+\frac{1}{{\left(y^{\prime }\left(s_{2}\right)\right)}^{2}}+\frac{1}{{\left(z^{\prime }\left(s_{2}\right)\right)}^{2}}\right)}\right]=\\ =\psi \left(s\right)exp[-\int_{s_{1}}^{s}ds_{2}\left(\frac{\left[x^{\prime \prime }\left(s_{2}\right){\left(y^{\prime }\left(s_{2}\right)z^{\prime }\left(s_{2}\right)\right)}^{2}+y^{\prime \prime }\left(s_{2}\right){\left(x^{\prime }\left(s_{2}\right)z^{\prime }\left(s_{2}\right)\right)}^{2}+z^{\prime \prime }\left(s_{2}\right){\left(x^{\prime }\left(s_{2}\right)y^{\prime }\left(s_{2}\right)\right)}^{2}\right]}{\left[{\left(y^{\prime }\left(s_{2}\right)z^{\prime }\left(s_{2}\right)\right)}^{2}+{\left(x^{\prime }\left(s_{2}\right)y^{\prime }\left(s_{2}\right)\right)}^{2}+{\left(x^{\prime }\left(s_{2}\right)z^{\prime }\left(s_{2}\right)\right)}^{2}\right]}\right)],\\ \psi \left(s\right)=exp\left[+\int_{s_{1}}^{s}ds_{2}\frac{g(s_{2})}{f(s_{2})}\right]\psi_{1}\left(s\right)=exp\left[+\int_{s_{1}}^{s}ds_{2}\left(\frac{\left[x^{\prime \prime }\left(s_{2}\right){\left(y^{\prime }\left(s_{2}\right)z^{\prime }\left(s_{2}\right)\right)}^{2}+y^{\prime \prime }\left(s_{2}\right){\left(x^{\prime }\left(s_{2}\right)z^{\prime }\left(s_{2}\right)\right)}^{2}+z^{\prime \prime }\left(s_{2}\right){\left(x^{\prime }\left(s_{2}\right)y^{\prime }\left(s_{2}\right)\right)}^{2}\right]}{\left[{\left(y^{\prime }\left(s_{2}\right)z^{\prime }\left(s_{2}\right)\right)}^{2}+{\left(x^{\prime }\left(s_{2}\right)y^{\prime }\left(s_{2}\right)\right)}^{2}+{\left(x^{\prime }\left(s_{2}\right)z^{\prime }\left(s_{2}\right)\right)}^{2}\right]}\right)\right]\psi_{1}\left(s\right). \end{array}$$

In the very same way, in two dimensions we have

(79) $$\begin{array}{c} \psi_{1}\left(s\right)=\psi \left(s\right)exp\left[\frac{i}{\hbar }\int_{s_{1}}^{s}ds_{2}i\frac{1}{2}\frac{g(s_{2})}{f(s_{2})}\hbar \right]=\psi \left(s\right)exp\left[-\int_{s_{1}}^{s}ds_{2}\frac{g(s_{2})}{f(s_{2})}\right]=\psi \left(s\right)exp\left[-\int_{s_{1}}^{s}ds_{2}\frac{\frac{x^{\prime \prime }\left(s_{2}\right)}{{\left(x^{\prime }\left(s_{2}\right)\right)}^{2}}+\frac{y^{\prime \prime }\left(s_{2}\right)}{{\left(y^{\prime }\left(s_{2}\right)\right)}^{2}}}{\left(\frac{1}{{\left(x^{\prime }\left(s_{2}\right)\right)}^{2}}+\frac{1}{{\left(y^{\prime }\left(s_{2}\right)\right)}^{2}}\right)}\right]=\\ =\psi \left(s\right)exp\left[-\int_{s_{1}}^{s}ds_{2}\left(\frac{\left[x^{\prime \prime }\left(s_{2}\right){\left(y^{\prime }\left(s_{2}\right)\right)}^{2}+y^{\prime \prime }\left(s_{2}\right){\left(x^{\prime }\left(s_{2}\right)\right)}^{2}\right]}{\left[{\left(x^{\prime }\left(s_{2}\right)\right)}^{2}+{\left(y^{\prime }\left(s_{2}\right)\right)}^{2}\right]}\right)\right]=\psi_{1}\left(s\right),s=x,\psi \left(x\right)exp\left[-\int_{x_{1}}^{x}dx_{2}\left(\frac{\left[y^{\prime \prime }\left(x\right)\right]}{\left[1+{\left(y^{\prime }\left(x\right)\right)}^{2}\right]}\right)\right]=\\ =\psi \left(x\right)exp\left[-\int_{x_{1}}^{x}dx_{2}\left(\frac{\left[\frac{1}{cos{\left(\alpha \left(x_{2}\right)\right)}^{2}}\left(\frac{d}{dx_{2}}\alpha \left(x_{2}\right)\right)\right]}{\left[1+{\left(tan\left(\alpha \left(x\right)\right)\right)}^{2}\right]}\right)\right]=\psi \left(x\right)exp[-\int_{x_{1}}^{x}dx_{2}\frac{d}{dx_{2}}\alpha \left(x_{2}\right)={\alpha \left(x_{2}\right)|}_{x1}^{x}=\\ =\psi \left(x\right)Exp\left[-ArcTan\left[\frac{d}{dx}y\left(x\right)\right]{|}_{x_{2}=x1}^{x_{2}=x}\right]=\psi_{1}\left(x\right)=\\ =\psi \left(x\right)Exp[-\left(ArcTan\left[\frac{d}{dx}y\left(x\right)\right]-ArcTan\left[\frac{d}{dx_{1}}y\left(x_{1}\right)\right]\right)],\\ \psi \left(s\right)=exp\left[+\int_{s_{1}}^{s}ds_{2}\frac{g(s_{2})}{f(s_{2})}\right]\psi_{1}\left(s\right)=exp\left[+\int_{s_{1}}^{s}ds_{2}\left(\frac{\left[x^{\prime \prime }\left(s_{2}\right){\left(y^{\prime }\left(s_{2}\right)\right)}^{2}+y^{\prime \prime }\left(s_{2}\right){\left(x^{\prime }\left(s_{2}\right)\right)}^{2}\right]}{\left[{\left(x^{\prime }\left(s_{2}\right)\right)}^{2}+{\left(y^{\prime }\left(s_{2}\right)\right)}^{2}\right]}\right)\right]\psi_{1}\left(s\right),\\ s=x,\psi_{1}\left(x\right)exp\left[\int_{x_{1}}^{x}dx_{2}\left(\frac{\left[y^{\prime \prime }\left(x_{2}\right)\right]}{\left[1+{\left(y^{\prime }\left(x_{2}\right)\right)}^{2}\right]}\right)\right]=\psi_{1}\left(x\right)Exp\left[\left(ArcTan\left[\frac{d}{dx}y\left(x\right)\right]-ArcTan\left[\frac{d}{dx_{1}}y\left(x_{1}\right)\right]\right)\right]=\psi \left(x\right). \end{array}$$

We need to comment on term $\frac{d}{dx_{1}}y\left(x_{1}\right)$ expressing the semiconductor or superconducting cable curvature. We can orient our coordinate system in such a way that $x_{1}=0$ and $\frac{d}{dx_{1}}y\left(x_{1}\right)=0$. We observe that the newly introduced wave-function $\psi_{1}\left(s\right)$ fulfills the simplified GL curved equation given as

(80) $$\begin{array}{c} exp\left[+\int_{s_{1}}^{s}ds_{2}\frac{g(s_{2})}{f(s_{2})}\right]\left(\frac{1}{2m}{\left[\frac{\hbar }{i}\frac{d}{ds}\right]}^{2}\left(\psi \left(s\right)exp\left[-\int_{s_{1}}^{s}ds_{2}\frac{g(s_{2})}{f(s_{2})}\right]\right)\right)+\\ +\frac{1}{4m}\left[\hbar^{2}\frac{1}{2}{\left(\frac{g(s)}{f(s)}\right)}^{2}\right]\psi \left(s\right)+[\frac{\alpha (s)}{f(s)}+\frac{\beta (s)}{f(s)}{\left|\psi \left(s\right)\right|}^{2}\psi \left(s\right)=0. \end{array}$$

and fulfills the **Simplified Curved GL** equation in the form

(81) $$\begin{array}{c} exp\left[+\int_{s_{1}}^{s}ds_{2}\frac{g(s_{2})}{f(s_{2})}\right]\left(\frac{1}{2m}{\left[\frac{\hbar }{i}\frac{d}{ds}\right]}^{2}\left(\psi \left(s\right)exp\left[-\int_{s_{1}}^{s}ds_{2}\frac{g(s_{2})}{f(s_{2})}\right]\right)\right)+\\ +\frac{1}{4m}\left[\hbar^{2}\frac{1}{2}{\left(\frac{g(s)}{f(s)}\right)}^{2}\right]\psi \left(s\right)+[\frac{\alpha (s)}{f(s)}+\frac{\beta (s)}{f(s)}{\left|\psi \left(s\right)\right|}^{2}\psi \left(s\right)=0. \end{array}$$

This is due to the fact that

(82) $$\begin{array}{c} (\frac{1}{2m}{\left[\frac{\hbar }{i}\frac{d}{ds}\right]}^{2}\left(\psi \left(s\right)exp\left[-\int_{s_{1}}^{s}ds_{2}\frac{g(s_{2})}{f(s_{2})}\right]\right))+\\ +(\frac{1}{4m}\left[\hbar^{2}\frac{1}{2}{\left(\frac{g(s)}{f(s)}\right)}^{2}\right]+[\frac{\alpha (s)}{f(s)}+\frac{\beta (s)}{f(s)}|\psi \left(s\right){|}^{2})exp\left[-\int_{s_{1}}^{s}ds_{2}\frac{g(s_{2})}{f(s_{2})}\right]\psi \left(s\right)=0. \end{array}$$

which results in the equation

(83) $$\begin{array}{c} (\frac{1}{2m}{\left[\frac{\hbar }{i}\frac{d}{ds}\right]}^{2}\left(\psi_{1}\left(s\right)\right)+(\frac{1}{4m}\left[\hbar^{2}\frac{1}{2}{\left(\frac{g(s)}{f(s)}\right)}^{2}\right]+[\frac{\alpha (s)}{f(s)}+\frac{\beta (s)}{f(s)}|\psi \left(s\right){|}^{2})\psi_{1}\left(s\right)=0. \end{array}$$

and also in the equation

(84) $$\begin{array}{c} (\frac{1}{2m}{\left[\frac{\hbar }{i}\frac{d}{ds}\right]}^{2}\left(\psi_{1}\left(s\right)\right)+(\frac{1}{4m}\left[\hbar^{2}\frac{1}{2}{\left(\frac{g(s)}{f(s)}\right)}^{2}\right]+[\frac{\alpha (s)}{f(s)}+\frac{\beta (s)}{f(s)}exp\left[-\int_{s_{1}}^{s}ds_{2}\frac{g(s_{2})}{f(s_{2})}\right]|\psi_{1}\left(s\right){|}^{2})\psi_{1}\left(s\right)=0. \end{array}$$

In the case of a Schrödinger bended equation, we obtain

(85) $$\begin{array}{c} \frac{1}{2m}{\left[\frac{\hbar }{i}\frac{d}{ds}\right]}^{2}\psi_{1}\left(s\right)+\frac{1}{4m}\left[\hbar^{2}\frac{1}{2}{\left(\frac{g(s)}{f(s)}\right)}^{2}\right]\psi {\left(s\right)}_{1}+\left[\frac{V(s)-E}{f(s)}+E_{b}\right]\psi {\left(s\right)}_{1}=E_{b}\psi {\left(s\right)}_{1}. \end{array}$$

which can be written as

(86) $$\begin{array}{c} \frac{1}{2m}{\left[\frac{\hbar }{i}\frac{d}{ds}\right]}^{2}\psi_{1}\left(s\right)+\frac{1}{4m}\left[\hbar^{2}\frac{1}{2}{\left(\frac{\frac{x^{\prime \prime }\left(s\right)}{{\left(x^{\prime }\left(s\right)\right)}^{2}}+\frac{y^{\prime \prime }\left(s\right)}{{\left(y^{\prime }\left(s\right)\right)}^{2}}+\frac{z^{\prime \prime }\left(s\right)}{{\left(z^{\prime }\left(s\right)\right)}^{2}}}{\left(\frac{1}{{\left(x^{\prime }\left(s\right)\right)}^{2}}+\frac{1}{{\left(y^{\prime }\left(s\right)\right)}^{2}}+\frac{1}{{\left(z^{\prime }\left(s\right)\right)}^{2}}\right)}\right)}^{2}\right]\psi {\left(s\right)}_{1}+\left[\frac{V\left(s\right)-E_{straight}}{\left(\frac{1}{{\left(x^{\prime }\left(s\right)\right)}^{2}}+\frac{1}{{\left(y^{\prime }\left(s\right)\right)}^{2}}+\frac{1}{{\left(z^{\prime }\left(s\right)\right)}^{2}}\right)}+E_{b}\right]\psi {\left(s\right)}_{1}=E_{b}\psi {\left(s\right)}_{1}. \end{array}$$

(87) $$\begin{array}{c} \frac{1}{2m}{\left[\frac{\hbar }{i}\frac{d}{ds}\right]}^{2}\psi_{1}\left(s\right)+[\frac{\hbar^{2}}{2m}\frac{1}{4}{\left[\frac{\left[x^{\prime \prime }\left(s\right){\left(y^{\prime }\left(s\right)z^{\prime }\left(s\right)\right)}^{2}+y^{\prime \prime }\left(s\right){\left(x^{\prime }\left(s\right)z^{\prime }\left(s\right)\right)}^{2}+z^{\prime \prime }\left(s\right){\left(x^{\prime }\left(s\right)y^{\prime }\left(s\right)\right)}^{2}\right]}{{\left(x^{\prime }\left(s\right)y^{\prime }\left(s\right)\right)}^{2}+{\left(x^{\prime }\left(s\right)z^{\prime }\left(s\right)\right)}^{2}+{\left(y^{\prime }\left(s\right)z^{\prime }\left(s\right)\right)}^{2}}\right]}^{2}+\\ +\left[\frac{\left(V\left(s\right)-E_{s}\right){\left(x^{\prime }\left(s\right)y^{\prime }\left(s\right)z^{\prime }\left(s\right)\right)}^{2}}{{\left(x^{\prime }\left(s\right)y^{\prime }\left(s\right)\right)}^{2}+{\left(x^{\prime }\left(s\right)z^{\prime }\left(s\right)\right)}^{2}+{\left(y^{\prime }\left(s\right)z^{\prime }\left(s\right)\right)}^{2}}+E_{b}\right]\psi {\left(s\right)}_{1}=E_{b}\psi {\left(s\right)}_{1}. \end{array}$$

In two dimensions, we have

(88) $$\begin{array}{c} \frac{1}{2m}{\left[\frac{\hbar }{i}\frac{d}{ds}\right]}^{2}\psi_{1}\left(s\right)+[\frac{\hbar^{2}}{2m}\frac{1}{4}{\left[\frac{\left[x^{\prime \prime }\left(s\right){\left(y^{\prime }\left(s\right)\right)}^{2}+y^{\prime \prime }\left(s\right){\left(x^{\prime }\left(s\right)\right)}^{2}\right]}{{\left(x^{\prime }\left(s\right)\right)}^{2}+{\left(y^{\prime }\left(s\right)\right)}^{2}}\right]}^{2}+\left[\frac{\left(V\left(s\right)-E_{s}\right){\left(x^{\prime }\left(s\right)y^{\prime }\left(s\right)\right)}^{2}}{{\left(x^{\prime }\left(s\right)\right)}^{2}+{\left(y^{\prime }\left(s\right)\right)}^{2}}+E_{b}\right]\psi {\left(s\right)}_{1}=E_{b}\psi {\left(s\right)}_{1}. \end{array}$$

and for s=x, x(s)=x(x)=x, y(s)=y(x) so we obtain

(89) $$\begin{array}{c} \frac{1}{2m}{\left[\frac{\hbar }{i}\frac{d}{dx}\right]}^{2}\psi_{1}\left(x\right)+[\frac{\hbar^{2}}{2m}\frac{1}{4}{\left[\frac{\left[y^{\prime \prime }\left(x\right)\right]}{1+{\left(y^{\prime }\left(x\right)\right)}^{2}}\right]}^{2}+\left[\frac{\left(V\left(x\right)-E_{s}\right){\left(y^{\prime }\left(x\right)\right)}^{2}}{1+{\left(y^{\prime }\left(x\right)\right)}^{2}}+E_{b}\right]\psi {\left(s\right)}_{1}=E_{b}\psi {\left(x\right)}_{1}. \end{array}$$

and by introducing $y^{\prime }\left(x\right)=tan\left(\alpha \left(x\right)\right)$ we have

(90) $$\begin{array}{c} \frac{1}{2m}{\left[\frac{\hbar }{i}\frac{d}{dx}\right]}^{2}\psi_{1}\left(x\right)+[\frac{\hbar^{2}}{2m}\frac{1}{4}{\left[\frac{\left[\frac{1}{cos{\left(\alpha \left(x\right)\right)}^{2}}\frac{d}{dx}\alpha \left(x\right)\right]}{1+{\left(tan\left(\alpha \left(x\right)\right)\right)}^{2}}\right]}^{2}+\left[\frac{\left(V\left(x\right)-E_{s}\right){\left(tan\left(\alpha \left(x\right)\right)\right)}^{2}}{1+{\left(tan\left(\alpha \left(x\right)\right)\right)}^{2}}+E_{b}\right]\psi {\left(s\right)}_{1}=E_{b}\psi {\left(x\right)}_{1}. \end{array}$$

and we obtain

(91) $$\begin{array}{c} \frac{1}{2m}{\left[\frac{\hbar }{i}\frac{d}{dx}\right]}^{2}\psi_{1}\left(x\right)+\left[-\frac{1}{2m}{\left(\frac{\hbar }{i}\right)}^{2}\frac{1}{4}{\left[\frac{d}{dx}\alpha \left(x\right)\right]}^{2}+\left[\frac{\left(V\left(x\right)-E_{s}\right){\left(tan\left(\alpha \left(x\right)\right)\right)}^{2}}{1+{\left(tan\left(\alpha \left(x\right)\right)\right)}^{2}}+E_{b}\right]\right]\psi {\left(s\right)}_{1}=E_{b}\psi {\left(x\right)}_{1}. \end{array}$$

We recognize that $-\frac{1}{2m}{\left(\frac{\hbar }{i}\right)}^{2}\frac{1}{4}{\left[\frac{d}{dx}\alpha \left(x\right)\right]}^{2}$ is acting against the kinetic energy term $\frac{1}{2m}{\left[\frac{\hbar }{i}\frac{d}{dx}\right]}^{2}\psi_{1}\left(x\right)$.

(92) $$\begin{array}{c} f\left(s\right)=\left(\frac{1}{{\left(x^{\prime }\left(s\right)\right)}^{2}}+\frac{1}{{\left(y^{\prime }\left(s\right)\right)}^{2}}+\frac{1}{{\left(z^{\prime }\left(s\right)\right)}^{2}}\right),g\left(s\right)=\left(\frac{x^{\prime \prime }\left(s\right)}{{\left(x^{\prime }\left(s\right)\right)}^{2}}+\frac{y^{\prime \prime }\left(s\right)}{{\left(y^{\prime }\left(s\right)\right)}^{2}}+\frac{z^{\prime \prime }\left(s\right)}{{\left(z^{\prime }\left(s\right)\right)}^{2}}\right), \end{array}$$

and $y\left(x\right)={\left(Tanh\left(2x\right)\right)}^{2},\frac{d}{dx}y\left(x\right)=4\tanh\left(2x\right){\sec h}^{2}\left(2x\right)$, and $\frac{d^{2}}{dx^{2}}{\left(Tanh\left[2x\right]\right)}^{2}=8Sech{\left[2x\right]}^{4}$$-16Sech{\left[2x\right]}^{2}Tanh{\left[2x\right]}^{2}$ and

(93) $$\begin{array}{c} {\left[\frac{\frac{d^{2}}{dx^{2}}y\left(x\right)}{\left(1+{\left(\frac{d}{dx}y\left(x\right)\right)}^{2}\right)}\right]}^{2}=\frac{64{\left(\cosh\left(4x\right)-2\right)}^{2}{\sec h}^{8}\left(2x\right)}{{\left(16\tanh^{2}\left(2x\right){\sec h}^{4}\left(2x\right)+1\right)}^{2}} \end{array}$$

as depicted in Figure 5.

### 4.3. Case of GL and Schrödinger Equations in Curved Space with Non-Zero Vector Potential

In the next step, we parametrize vector potential $\vec{A}\left(s\right)=\left(A_{x}\left(s\right),A_{y}\left(s\right),A_{z}\left(s\right)\right)$ by the s variable and we obtain a Schrödinger equation in the form

(94) $$\frac{1}{2m}\left[{\left(\frac{\hbar }{i}\frac{d}{dx}-\frac{e}{c}A_{x}\left(s\right)\right)}^{2}+{\left(\frac{\hbar }{i}\frac{d}{dy}-\frac{e}{c}A_{y}\left(s\right)\right)}^{2}+{\left(\frac{\hbar }{i}\frac{d}{dz}-\frac{e}{c}A_{z}\left(s\right)\right)}^{2}\right]\psi \left(x,y,z\right)+V\left(x,y,z\right)\psi \left(x,y,z\right)=E\psi \left(x,y,z\right)$$

and the corresponding Ginzburg–Landau equation in the form

(95) $$\frac{1}{2m}\left[{\left(\frac{\hbar }{i}\frac{d}{dx}-\frac{2e}{c}A_{x}\left(s\right)\right)}^{2}+{\left(\frac{\hbar }{i}\frac{d}{dy}-\frac{2e}{c}A_{y}\left(s\right)\right)}^{2}+{\left(\frac{\hbar }{i}\frac{d}{dz}-\frac{2e}{c}A_{z}\left(s\right)\right)}^{2}\right]{\psi \left(x,y,z\right)+(\alpha \left(x,y,z\right)+\beta \left(x,y,z\right)|\psi \left(x,y,z\right)|}^{2})\psi \left(x,y,z\right)=0.$$

We obtain the kinetic energy operator in the form

(96) $$\begin{array}{c} {\left(\frac{\hbar }{i}\frac{d}{dx}-\frac{2e}{c}A_{x}\left(s\right)\right)}^{2}={\left(\frac{\hbar }{i}\frac{ds}{dx}\frac{d}{ds}-\frac{2e}{c}A_{x}\left(s\right)\right)}^{2}={\left[\frac{\hbar }{i}\frac{ds}{dx}\frac{d}{ds}\right]}^{2}+{\left[\frac{2e}{c}A_{x}\left(s\right)\right]}^{2}+2i\hbar \frac{2e}{c}A_{x}\left(s\right)\frac{d}{ds}+i\hbar \frac{2e}{c}\left(\frac{d}{ds}A_{x}\left(s\right)\right)=\\ =-\hbar^{2}\frac{1}{{\left(x^{\prime }\left(s\right)\right)}^{2}}\frac{d^{2}}{ds^{2}}-\frac{x^{\prime \prime }\left(s\right)}{{\left(x^{\prime }\left(s\right)\right)}^{2}}\frac{d}{ds}+{\left[\frac{2e}{c}A_{x}\left(s\right)\right]}^{2}+2i\hbar \frac{2e}{c}A_{x}\left(s\right)\frac{d}{ds}+i\hbar \frac{2e}{c}\left(\frac{d}{ds}A_{x}\left(s\right)\right)=\\ =-\hbar^{2}\frac{1}{{\left(x^{\prime }\left(s\right)\right)}^{2}}\frac{d^{2}}{ds^{2}}+i\left(i\frac{x^{\prime \prime }\left(s\right)}{{\left(x^{\prime }\left(s\right)\right)}^{2}}+2\hbar \frac{2e}{c}A_{x}\left(s\right)\right)\frac{d}{ds}+\left[{\left[\frac{2e}{c}A_{x}\left(s\right)\right]}^{2}+i\hbar \frac{2e}{c}\left(\frac{d}{ds}A_{x}\left(s\right)\right)\right] \end{array}$$

and thus we have

(97) $$\begin{array}{c} {\left(\frac{\hbar }{i}\frac{d}{dx}-\frac{2e}{c}A_{x}\left(s\right)\right)}^{2}+{\left(\frac{\hbar }{i}\frac{d}{dy}-\frac{2e}{c}A_{y}\left(s\right)\right)}^{2}+{\left(\frac{\hbar }{i}\frac{d}{dz}-\frac{2e}{c}A_{z}\left(s\right)\right)}^{2}=\\ =-\hbar^{2}\left[\frac{1}{{\left(x^{\prime }\left(s\right)\right)}^{2}}+\frac{1}{{\left(y^{\prime }\left(s\right)\right)}^{2}}+\frac{1}{{\left(z^{\prime }\left(s\right)\right)}^{2}}\right]\frac{d^{2}}{ds^{2}}+\\ +i\left(i\left(\frac{x^{\prime \prime }\left(s\right)}{{\left(x^{\prime }\left(s\right)\right)}^{2}}+\frac{y^{\prime \prime }\left(s\right)}{{\left(y^{\prime }\left(s\right)\right)}^{2}}+\frac{z^{\prime \prime }\left(s\right)}{{\left(z^{\prime }\left(s\right)\right)}^{2}}\right)+2\hbar \frac{2e}{c}\left(A_{x}\left(s\right)+A_{y}\left(s\right)+A_{z}\left(s\right)\right)\right)\frac{d}{ds}+ \end{array}$$

(98) $$\begin{array}{c} +\left[{\left[\frac{2e}{c}A_{x}\left(s\right)\right]}^{2}+{\left[\frac{2e}{c}A_{y}\left(s\right)\right]}^{2}+{\left[\frac{2e}{c}A_{z}\left(s\right)\right]}^{2}+i\hbar \frac{2e}{c}\left(\frac{d}{ds}\left(A_{x}\left(s\right)+A_{y}\left(s\right)+A_{z}\left(s\right)\right)\right)\right]=-\hbar^{2}\left[f_{q}\left(s\right)\frac{d^{2}}{ds^{2}}-g_{q}\left(s\right)\frac{d}{ds}+r_{q}\left(s\right)\right], \end{array}$$

with

(99) $$\begin{array}{c} f_{q}\left(s\right)=\frac{1}{{\left(x^{\prime }\left(s\right)\right)}^{2}}+\frac{1}{{\left(y^{\prime }\left(s\right)\right)}^{2}}+\frac{1}{{\left(z^{\prime }\left(s\right)\right)}^{2}},\\ g_{q}\left(s\right)=\frac{-1}{\hbar^{2}}i\left(i\left(\frac{x^{\prime \prime }\left(s\right)}{{\left(x^{\prime }\left(s\right)\right)}^{2}}+\frac{y^{\prime \prime }\left(s\right)}{{\left(y^{\prime }\left(s\right)\right)}^{2}}+\frac{z^{\prime \prime }\left(s\right)}{{\left(z^{\prime }\left(s\right)\right)}^{2}}\right)+2\hbar \frac{2e}{c}\left(A_{x}\left(s\right)+A_{y}\left(s\right)+A_{z}\left(s\right)\right)\right),\\ r_{q}\left(s\right)=-\frac{1}{\hbar^{2}}\left[{\left[\frac{2e}{c}A_{x}\left(s\right)\right]}^{2}+{\left[\frac{2e}{c}A_{y}\left(s\right)\right]}^{2}+{\left[\frac{2e}{c}A_{z}\left(s\right)\right]}^{2}+i\hbar \frac{2e}{c}\left(\frac{d}{ds}\left(A_{x}\left(s\right)+A_{y}\left(s\right)+A_{z}\left(s\right)\right)\right)\right],\\ g_{q1}\left(s\right)=\frac{g_{q}\left(s\right)}{f_{q}\left(s\right)}=\frac{\frac{-1}{\hbar^{2}}i\left(i\left(\frac{x^{\prime \prime }\left(s\right)}{{\left(x^{\prime }\left(s\right)\right)}^{2}}+\frac{y^{\prime \prime }\left(s\right)}{{\left(y^{\prime }\left(s\right)\right)}^{2}}+\frac{z^{\prime \prime }\left(s\right)}{{\left(z^{\prime }\left(s\right)\right)}^{2}}\right)+2\hbar \frac{2e}{c}\left(A_{x}\left(s\right)+A_{y}\left(s\right)+A_{z}\left(s\right)\right)\right)}{\frac{1}{{\left(x^{\prime }\left(s\right)\right)}^{2}}+\frac{1}{{\left(y^{\prime }\left(s\right)\right)}^{2}}+\frac{1}{{\left(z^{\prime }\left(s\right)\right)}^{2}}}=\\ =\frac{\frac{1}{\hbar^{2}}\left(\left[x^{\prime \prime }\left(s\right){\left(y^{\prime }\left(s\right)z^{\prime }\left(s\right)\right)}^{2}+y^{\prime \prime }\left(s\right){\left(x^{\prime }\left(s\right)z^{\prime }\left(s\right)\right)}^{2}+z^{\prime \prime }\left(s\right){\left(x^{\prime }\left(s\right)y^{\prime }\left(s\right)\right)}^{2}\right]-2i\hbar \frac{2e}{c}\left(A_{x}\left(s\right)+A_{y}\left(s\right)+A_{z}\left(s\right)\right){\left(x^{\prime }\left(s\right)y^{\prime }\left(s\right)z^{\prime }\left(s\right)\right)}^{2}\right)}{{\left(x^{\prime }\left(s\right)y^{\prime }\left(s\right)\right)}^{2}+{\left(x^{\prime }\left(s\right)z^{\prime }\left(s\right)\right)}^{2}+{\left(y^{\prime }\left(s\right)z^{\prime }\left(s\right)\right)}^{2}}. \end{array}$$

In two dimensions, we have

(100) $$\begin{array}{c} g_{q1}\left(s=x\right)=\frac{g_{q}\left(s\right)}{f_{q}\left(s\right)}=\frac{\frac{-1}{\hbar^{2}}i\left(i\left(\frac{y^{\prime \prime }\left(s\right)}{{\left(y^{\prime }\left(s\right)\right)}^{2}}\right)+2\hbar \frac{2e}{c}\left(A_{x}\left(s\right)+A_{y}\left(s\right)\right)\right)}{\frac{1}{{\left(x^{\prime }\left(s\right)\right)}^{2}}+\frac{1}{{\left(y^{\prime }\left(s\right)\right)}^{2}}}=\\ =\frac{\frac{1}{\hbar^{2}}\left(\left[y^{\prime \prime }\left(s\right){\left(x^{\prime }\left(s\right)\right)}^{2}\right]-2i\hbar \frac{2e}{c}\left(A_{x}\left(s\right)+A_{y}\left(s\right)\right){\left(x^{\prime }\left(s\right)y^{\prime }\left(s\right)\right)}^{2}\right)}{{\left(x^{\prime }\left(s\right)\right)}^{2}+{\left(y^{\prime }\left(s\right)\right)}^{2}}=\\ =\frac{\frac{1}{\hbar^{2}}\left(\left[y^{\prime \prime }\left(x\right)\right]-2i\hbar \frac{2e}{c}\left(A_{x}\left(x\right)+A_{y}\left(x\right)\right){\left(y^{\prime }\left(x\right)\right)}^{2}\right)}{1+{\left(y^{\prime }\left(x\right)\right)}^{2}}. \end{array}$$

In the case of any form of two-dimensional vector potential field $\left(A_{x}\left(x,y\left(x\right)\right),A_{y}\left(x,y\left(x\right)\right)\right)$$=(A_{x}\left(x,y\left(x\right)\right),A_{y}\left(x,y\left(x\right)\right))$, we recognize that

(101) $$\begin{array}{c} \int_{x_{0}}^{x}dx_{1}\frac{\frac{1}{\hbar^{2}}{\left(\left[y^{\prime \prime }\left(x_{1}\right)\right]-2i\hbar \frac{2e}{c}\left(A_{x}\left(x_{1}\right)+A_{y}\left(x_{1}\right)\right)y^{\prime }\left(x_{1}\right)\right)}^{2})}{1+{\left(y^{\prime }\left(x_{1}\right)\right)}^{2}}=\\ =\int_{x_{0}}^{x}dx_{1}\frac{\frac{1}{\hbar^{2}}\left[y^{\prime \prime }\left(x_{1}\right)\right]}{1+{\left(y^{\prime }\left(x_{1}\right)\right)}^{2}}-\int_{x_{0}}^{x}dx_{1}2i\frac{\hbar \frac{2e}{c}\left(A_{x}\left(x_{1}\right)+A_{y}\left(x_{1}\right)\right){\left(y^{\prime }\left(x_{1}\right)\right)}^{2})}{1+{\left(y^{\prime }\left(x_{1}\right)\right)}^{2}}=\\ =\int_{x_{0}}^{x}dx_{1}\left(\frac{d}{dx_{1}}\alpha \left(x_{1}\right)\right)-2i\int_{x_{0}}^{x}dx_{1}\hbar \frac{2e}{c}\left(A_{x}\left(x_{1}\right)+A_{y}\left(x_{1}\right)\right){\left(sin\left(\alpha \left(x_{1}\right)\right)\right)}^{2}=\\ =\int_{x_{0}}^{x}dx_{1}\left(\frac{d}{dx_{1}}ArcTan\left(y^{\prime }\left(x_{1}\right)\right)\right)-2i\int_{x_{0}}^{x}dx_{1}\hbar \frac{2e}{c}\left(A_{x}\left(x_{1}\right)+A_{y}\left(x_{1}\right)\right){\left(sin\left(ArcTan\left(y^{\prime }\left(x_{1}\right)\right)\right)\right)}^{2}=\\ =ArcTan\left[y^{\prime }\left(x_{1}\right)\right]{|}_{x_{1}=x_{0}}^{x_{1}=x}-2i\left[\int_{x_{0}}^{x_{s}}dx_{1}\hbar \frac{2e}{c}\left(A_{x}\left(x_{1}\right)+A_{y}\left(x_{1}\right)\right)\right]{\left(sin\left(\alpha \left(x_{s}\right)\right)\right)}^{2}{|}_{x_{s}=x_{0}}^{x_{s}=x}\\ +2i\int_{x_{0}}^{x}sin\left(\alpha \left(x_{1}\right)\right)cos\left(\alpha \left(x_{1}\right)\right)\frac{d\alpha (x_{1})}{dx_{1}}\hbar \frac{2e}{c}\left[\int_{x_{0}}^{x_{1}}\left(A_{x}\left(x_{2}\right)+A_{y}\left(x_{2}\right)\right)dx_{2}\right] \end{array}$$

Under the assumption of constant vector potential components in space, we have

(102) $$\begin{array}{c} \int_{x_{0}}^{x}dx_{2}g_{q1}\left(x_{2}\right)=ArcTan\left[y^{\prime }\left(x_{1}\right)\right]{|}_{x_{1}=x_{0}}^{x_{1}=x}-2i\left[\left(x-x_{0}\right)\hbar \frac{2e}{c}\left(A_{x}\left(x\right)+A_{y}\left(x\right)\right)\right]{\left(sin\left(\alpha \left(x\right)\right)\right)}^{2}\\ +2i\int_{x_{0}}^{x}dx_{1}sin\left(\alpha \left(x_{1}\right)\right)cos\left(\alpha \left(x_{1}\right)\right)\frac{d\alpha (x_{1})}{dx_{1}}\hbar \frac{2e}{c}\left(A_{x}\left(x_{0}\right)+A_{y}\left(x_{0}\right)\right)\left(x_{1}-x_{0}\right)=\\ =ArcTan\left[y^{\prime }\left(x_{1}\right)\right]{|}_{x_{1}=x_{0}}^{x_{1}=x}-2i\left[\left(x-x_{0}\right)\hbar \frac{2e}{c}\left(A_{x}\left(x\right)+A_{y}\left(x\right)\right)\right]{\left(sin\left(\alpha \left(x\right)\right)\right)}^{2}\\ +2i\hbar \frac{2e}{c}\left(A_{x}+A_{y}\right)\int_{x_{0}}^{x}dx_{1}\frac{d}{dx_{1}}\left[sin{\left(\alpha \left(x_{1}\right)\right)}^{2}\right]=\\ =ArcTan\left[y^{\prime }\left(x_{1}\right)\right]{|}_{x_{1}=x_{0}}^{x_{1}=x}-2i\left[\left(x-x_{0}\right)\hbar \frac{2e}{c}\left(A_{x}+A_{y}\right)\right)]{\left(sin\left(\alpha \left(x\right)\right)\right)}^{2}\\ +2i\hbar \frac{2e}{c}\left(A_{x}+A_{y}\right)\left[sin{\left(\alpha \left(x\right)\right)}^{2}-sin{\left(\alpha \left(x_{0}\right)\right)}^{2}\right]=\\ =\left(ArcTan\left[y^{\prime }\left(x\right)\right]-ArcTan\left[y^{\prime }\left(x_{0}\right)\right]\right)-2i\left[\left(x-x_{0}\right)\hbar \frac{2e}{c}\left(A_{x}+A_{y}\right)\right)]\left(\frac{{\left(y^{\prime }\left[x\right]\right)}^{2}}{1+{\left(y^{\prime }\left[x\right]\right)}^{2}}\right)+\\ +2i\hbar \frac{2e}{c}\left(A_{x}+A_{y}\right)\left[\frac{y^{\prime }{\left[x\right]}^{2}}{1+{\left(y^{\prime }\left[x\right]\right)}^{2}}-\frac{y^{\prime }{\left[x_{0}\right]}^{2}}{1+{\left(y^{\prime }\left[x_{0}\right]\right)}^{2}}\right]. \end{array}$$

The previous equation implies

(103) $$\begin{array}{c} -\frac{\hbar^{2}}{2m}\left[f_{q}\left(s\right)\frac{d^{2}}{ds^{2}}-g_{q}\left(s\right)\frac{d}{ds}+r_{q}\left(s\right)\right]{\psi \left(s\right)+[\alpha \left(s\right)+\beta \left(s\right)|\psi \left(s\right)|}^{2}]\psi \left(s\right)=0. \end{array}$$

and we obtain

(104) $$\begin{array}{c} -\frac{\hbar^{2}}{2m}\left[\frac{d^{2}}{ds^{2}}-\frac{g_{q}\left(s\right)}{f_{q}\left(s\right)}\frac{d}{ds}+\frac{r_{q}\left(s\right)}{f_{q}\left(s\right)}\right]\psi \left(s\right)+[\frac{\alpha (s)}{f_{q}\left(s\right)}-\frac{\hbar^{2}}{2m}r_{q1}\left(s\right)+\frac{\beta_{q1}\left(s\right)}{f_{q}\left(s\right)}|\psi \left(s\right){|}^{2}]\psi \left(s\right)=0. \end{array}$$

which can be rewritten in the form

(105) $$\begin{array}{c} -\frac{\hbar^{2}}{2m}\left[\frac{d^{2}}{ds^{2}}-g_{q1}\left(s\right)\frac{d}{ds}\right]\psi \left(s\right)+[\alpha_{q1}\left(s\right)-\frac{\hbar^{2}}{2m}r_{q1}\left(s\right)+\beta_{q1}{\left(s\right)|\psi \left(s\right)|}^{2}]\psi \left(s\right)=0. \end{array}$$

and recognized to be of the form

(106) $$\begin{array}{c} \frac{1}{2m}{\left[\frac{\hbar }{i}\frac{d}{ds}+ia_{q1}\left(s\right)\hbar \right]}^{2}\psi \left(s\right)+u\left(s\right)\psi \left(s\right)+[\alpha_{q1}\left(s\right)-\frac{\hbar^{2}}{2m}r_{q1}\left(s\right)+\beta {\left(s\right)}_{q1}{\left|\psi \left(s\right)\right|}^{2}]\psi \left(s\right)=0. \end{array}$$

so one has

(107) $$\begin{array}{c} \frac{1}{2m}\left[-\hbar^{2}\frac{d^{2}}{ds^{2}}-\hbar^{2}{\left(a_{q1}\left(s\right)\right)}^{2}-i2\hbar a_{q1}\left(s\right)\frac{d}{ds}-i\hbar \left(\frac{d}{ds}a_{q1}\left(s\right)\right)\right]\psi \left(s\right)+\frac{1}{2m}\left(\hbar^{2}{\left(a_{q1}\left(s\right)\right)}^{2}+i\hbar \left(\frac{d}{ds}a_{q1}\left(s\right)\right)\right)\psi \left(s\right)+\\ +[\alpha_{q1}\left(s\right)-\frac{\hbar^{2}}{2m}r_{q1}\left(s\right)+\beta {\left(s\right)}_{q1}|\psi \left(s\right){|}^{2}]\psi \left(s\right)=0. \end{array}$$

Finally, one can write in a compact way that

(108) $$\begin{array}{c} \frac{1}{2m}{\left[\frac{\hbar }{i}\frac{d}{ds}+\frac{1}{2}ig_{q1}\left(s\right)\hbar \right]}^{2}\psi \left(s\right)+\frac{1}{4m}\left[-\hbar^{2}\left(\frac{d}{ds}g_{q1}\left(s\right)\right)+\frac{1}{2}g_{q1}{\left(s\right)}^{2}\right]\psi \left(s\right)+[\alpha_{q1}\left(s\right)-\frac{\hbar^{2}}{2m}r_{q1}\left(s\right)+\beta {\left(s\right)}_{q1}{\left|\psi \left(s\right)\right|}^{2}]\psi \left(s\right)=0. \end{array}$$

We can encapsulate the last relation in a more compact form by assuming

(109) $$\begin{array}{c} \psi \left(s\right)=exp\left[\int_{s_{1}}^{s}ds_{2}\frac{1}{2}ig_{q1}\left(s_{2}\right)\hbar \right]\psi_{0}\left(s\right) \end{array}$$

and we obtain the equation

(110) $$\begin{array}{c} \frac{1}{2m}{\left[\frac{\hbar }{i}\frac{d}{ds}\right]}^{2}\psi \left(s\right)+\frac{1}{4m}\left[+\frac{1}{2}g_{q1}{\left(s\right)}^{2}\right]\psi \left(s\right)+[\alpha_{q1}\left(s\right)-\frac{\hbar^{2}}{2m}r_{q1}\left(s\right)+\beta {\left(s\right)}_{q1}|\psi \left(s\right){|}^{2}]\psi \left(s\right)=0. \end{array}$$

With the solution for

(111) $$\begin{array}{c} \frac{1}{2m}{\left[\frac{\hbar }{i}\frac{d}{ds}\right]}^{2}\psi {\left(s\right)}_{0}+\frac{1}{4m}\left[+\frac{1}{2}g_{q1}{\left(s\right)}^{2}\right]\psi {\left(s\right)}_{0}+[\alpha_{q1}\left(s\right)-\frac{\hbar^{2}}{2m}r_{q1}\left(s\right)+\beta {\left(s\right)}_{q1}exp\left[2\int_{s_{1}}^{s}ds_{2}\frac{1}{2}ig_{q1}\left(s_{2}\right)\hbar \right]|\psi {\left(s\right)}_{0}{|}^{2}]\psi {\left(s\right)}_{0}=0. \end{array}$$

We can immediately obtain

(112) $$\begin{array}{c} \psi \left(s\right)=exp\left[\int_{s_{1}}^{s}ds_{2}\frac{1}{2}ig_{q1}\left(s_{2}\right)\hbar \right]\psi_{0}\left(s\right) \end{array}$$

which is the solution for

(113) $$\begin{array}{c} \frac{1}{2m}{\left[\frac{\hbar }{i}\frac{d}{ds}\right]}^{2}\psi \left(s\right)+\frac{1}{4m}\left[+\frac{1}{2}g_{q1}{\left(s\right)}^{2}\right]\psi \left(s\right)+[\alpha_{q1}\left(s\right)-\frac{\hbar^{2}}{2m}r_{q1}\left(s\right)+\beta {\left(s\right)}_{q1}|\psi \left(s\right){|}^{2}]\psi \left(s\right)=0. \end{array}$$

The previous equation has a Schrödinger counterpart, which is

(114) $$\begin{array}{c} \frac{1}{2m}{\left[\frac{\hbar }{i}\frac{d}{ds}\right]}^{2}\psi \left(s\right)+\frac{1}{4m}\left[+\frac{1}{2}g_{q1}{\left(s\right)}^{2}\right]\psi \left(s\right)+\left[\frac{V_{q1}\left(s\right)}{f_{q}\left(s\right)}-\frac{E}{f_{q}\left(s\right)}-\frac{\hbar^{2}}{2m}r_{q1}\left(s\right)+E_{b}\right]\psi \left(s\right)=E_{b}\psi \left(s\right), \end{array}$$

and results in expression

(115) $$\begin{array}{c} \frac{1}{2m}{\left[\frac{\hbar }{i}\frac{d}{ds}\right]}^{2}\psi \left(s\right)+\frac{1}{8m}{\left[\frac{\frac{-1}{\hbar^{2}}i\left(i\left(\frac{x^{\prime \prime }\left(s\right)}{{\left(x^{\prime }\left(s\right)\right)}^{2}}+\frac{y^{\prime \prime }\left(s\right)}{{\left(y^{\prime }\left(s\right)\right)}^{2}}+\frac{z^{\prime \prime }\left(s\right)}{{\left(z^{\prime }\left(s\right)\right)}^{2}}\right)+2\hbar \frac{2e}{c}\left(A_{x}\left(s\right)+A_{y}\left(s\right)+A_{z}\left(s\right)\right)\right)}{\frac{1}{{\left(x^{\prime }\left(s\right)\right)}^{2}}+\frac{1}{{\left(y^{\prime }\left(s\right)\right)}^{2}}+\frac{1}{{\left(z^{\prime }\left(s\right)\right)}^{2}}}\right]}^{2}\psi \left(s\right)+\\ +\left[\frac{V_{q1}\left(s\right)-E+\frac{1}{2m}\left(+\left[{\left[\frac{2e}{c}A_{x}\left(s\right)\right]}^{2}+{\left[\frac{2e}{c}A_{y}\left(s\right)\right]}^{2}+{\left[\frac{2e}{c}A_{z}\left(s\right)\right]}^{2}+i\hbar \frac{2e}{c}\left(\frac{d}{ds}\left(A_{x}\left(s\right)+A_{y}\left(s\right)+A_{z}\left(s\right)\right)\right)\right]\right)}{\frac{1}{{\left(x^{\prime }\left(s\right)\right)}^{2}}+\frac{1}{{\left(y^{\prime }\left(s\right)\right)}^{2}}+\frac{1}{{\left(z^{\prime }\left(s\right)\right)}^{2}}}+E_{b}\right]\psi \left(s\right)=E_{b}\psi \left(s\right). \end{array}$$

Therefore, we arrive at equation form as

(116) $$\begin{array}{c} \frac{1}{2m}{\left[\frac{\hbar }{i}\frac{d}{ds}\right]}^{2}\psi \left(s\right)+\left[\frac{1}{8m}{\left[x^{\prime }\left(s\right)y^{\prime }\left(s\right)z^{\prime }\left(s\right)\right]}^{4}{\left[\frac{\frac{1}{\hbar^{2}}\left(1\left(\frac{x^{\prime \prime }\left(s\right)}{{\left(x^{\prime }\left(s\right)\right)}^{2}}+\frac{y^{\prime \prime }\left(s\right)}{{\left(y^{\prime }\left(s\right)\right)}^{2}}+\frac{z^{\prime \prime }\left(s\right)}{{\left(z^{\prime }\left(s\right)\right)}^{2}}\right)-2\hbar \frac{2e}{c}i\left(A_{x}\left(s\right)+A_{y}\left(s\right)+A_{z}\left(s\right)\right)\right)}{{\left(x^{\prime }\left(s\right)y^{\prime }\left(s\right)\right)}^{2}+{\left(y^{\prime }\left(s\right)z^{\prime }\left(s\right)\right)}^{2}+{\left(x^{\prime }\left(s\right)z^{\prime }\left(s\right)\right)}^{2}}\right]}^{2}\right]\psi \left(s\right)+\\ +\left[\left[\frac{V_{q1}\left(s\right)-E_{s}+\frac{1}{2m}\left(+\left[{\left[\frac{2e}{c}A_{x}\left(s\right)\right]}^{2}+{\left[\frac{2e}{c}A_{y}\left(s\right)\right]}^{2}+{\left[\frac{2e}{c}A_{z}\left(s\right)\right]}^{2}+i\hbar \frac{2e}{c}\left(\frac{d}{ds}\left(A_{x}\left(s\right)+A_{y}\left(s\right)+A_{z}\left(s\right)\right)\right)\right]\right)}{{\left(x^{\prime }\left(s\right)y^{\prime }\left(s\right)\right)}^{2}+{\left(y^{\prime }\left(s\right)z^{\prime }\left(s\right)\right)}^{2}+{\left(x^{\prime }\left(s\right)z^{\prime }\left(s\right)\right)}^{2}}\right]{\left[x^{\prime }\left(s\right)y^{\prime }\left(s\right)z^{\prime }\left(s\right)\right]}^{2}\right]\psi \left(s\right)=\\ +E_{b}\psi \left(s\right). \end{array}$$

and finally we obtain real and imaginary values of effective potential coming from nanowire non-zero curvature functions that give the quasi-one-dimensional Schrödinger equation of the form

(117) $$\begin{array}{c} \frac{1}{2m}{\left[\frac{\hbar }{i}\frac{d}{ds}\right]}^{2}\psi \left(s\right)+[E_{b}+\frac{1}{8m}{\left[x^{\prime }\left(s\right)y^{\prime }\left(s\right)z^{\prime }\left(s\right)\right]}^{4}\left[\frac{\frac{1}{\hbar^{4}}\left({\left(\frac{x^{\prime \prime }\left(s\right)}{{\left(x^{\prime }\left(s\right)\right)}^{2}}+\frac{y^{\prime \prime }\left(s\right)}{{\left(y^{\prime }\left(s\right)\right)}^{2}}+\frac{z^{\prime \prime }\left(s\right)}{{\left(z^{\prime }\left(s\right)\right)}^{2}}\right)}^{2}+4\hbar^{2}{\left(\frac{2e}{c}\right)}^{2}{\left(A_{x}\left(s\right)+A_{y}\left(s\right)+A_{z}\left(s\right)\right)}^{2}\right)}{{\left({\left(x^{\prime }\left(s\right)y^{\prime }\left(s\right)\right)}^{2}+{\left(y^{\prime }\left(s\right)z^{\prime }\left(s\right)\right)}^{2}+{\left(x^{\prime }\left(s\right)z^{\prime }\left(s\right)\right)}^{2}\right)}^{2}}\right)]+\\ +\left[\left[\frac{V_{q1}\left(s\right)-E_{s}+\frac{1}{2m}\left(+\left[{\left[\frac{2e}{c}A_{x}\left(s\right)\right]}^{2}+{\left[\frac{2e}{c}A_{y}\left(s\right)\right]}^{2}+{\left[\frac{2e}{c}A_{z}\left(s\right)\right]}^{2}\right]\right)}{{\left(x^{\prime }\left(s\right)y^{\prime }\left(s\right)\right)}^{2}+{\left(y^{\prime }\left(s\right)z^{\prime }\left(s\right)\right)}^{2}+{\left(x^{\prime }\left(s\right)z^{\prime }\left(s\right)\right)}^{2}}\right]{\left[x^{\prime }\left(s\right)y^{\prime }\left(s\right)z^{\prime }\left(s\right)\right]}^{2}\right]]\psi \left(s\right)+\\ +i[\frac{1}{8m}{\left[x^{\prime }\left(s\right)y^{\prime }\left(s\right)z^{\prime }\left(s\right)\right]}^{4}\left[\frac{\frac{1}{\hbar^{2}}\left(-2\left(\frac{x^{\prime \prime }\left(s\right)}{{\left(x^{\prime }\left(s\right)\right)}^{2}}+\frac{y^{\prime \prime }\left(s\right)}{{\left(y^{\prime }\left(s\right)\right)}^{2}}+\frac{z^{\prime \prime }\left(s\right)}{{\left(z^{\prime }\left(s\right)\right)}^{2}}\right)\hbar \frac{2e}{c}\left(A_{x}\left(s\right)+A_{y}\left(s\right)+A_{z}\left(s\right)\right)\right)}{{\left(x^{\prime }\left(s\right)y^{\prime }\left(s\right)\right)}^{2}+{\left(y^{\prime }\left(s\right)z^{\prime }\left(s\right)\right)}^{2}+{\left(x^{\prime }\left(s\right)z^{\prime }\left(s\right)\right)}^{2}}\right]\psi \left(s\right)+\\ +i{\left[x^{\prime }\left(s\right)y^{\prime }\left(s\right)z^{\prime }\left(s\right)\right]}^{2}\left[\frac{\left(\hbar \frac{2e}{c}\frac{d}{ds}\left(A_{x}\left(s\right)+A_{y}\left(s\right)+A_{z}\left(s\right)\right)\right)}{{\left(x^{\prime }\left(s\right)y^{\prime }\left(s\right)\right)}^{2}+{\left(y^{\prime }\left(s\right)z^{\prime }\left(s\right)\right)}^{2}+{\left(x^{\prime }\left(s\right)z^{\prime }\left(s\right)\right)}^{2}}\right]]\psi \left(s\right)=E_{b}\psi \left(s\right). \end{array}$$

## 5. Case of Two Electrostatically Interacting Single-Electron Lines in Schrödinger Formalism

The two parallel lines in the >< configuration with a single electron distributed at each line are expressed by two body Schrödinger modified equations in the form of

(118) $$\begin{array}{c} -\frac{\hbar^{2}}{2m_{A}}\left[\left(1+\frac{1}{\left(\frac{d}{dx_{A}}y_{A}{\left(x_{A}\right)}^{2}\right)}\right)\frac{d^{2}}{dx_{A}^{2}}-\frac{\frac{d^{2}}{dx_{A}^{2}}y_{A}\left(x_{A}\right)}{{\left(\frac{d}{dx_{A}}y\left(x_{A}\right)\right)}^{2}}\frac{d}{dx_{A}}\right]\psi \left(x_{A},y_{A},x_{B},y_{B}\right)\\ -\frac{\hbar^{2}}{2m_{B}}\left[\left(1+\frac{1}{\left(\frac{d}{dx_{B}}y_{B}{\left(x_{B}\right)}^{2}\right)}\right)\frac{d^{2}}{dx_{B}^{2}}-\frac{\frac{d^{2}}{dx_{B}^{2}}y_{B}\left(x_{B}\right)}{{\left(\frac{d}{dx_{B}}y\left(x_{B}\right)\right)}^{2}}\frac{d}{dx_{B}}\right]\psi \left(x_{A},y_{A},x_{B},y_{B}\right)+\\ +\left[V_{A}\left(x_{A}\right)+V_{B}\left(x_{B}\right)+V_{A-B}\left(x_{A},y_{A},x_{B},y_{B}\right)\right]\psi \left(x_{A},y_{A}\right)=E\psi \left(x_{A},y_{A},x_{B},y_{B}\right)=E\psi \left(s_{A},s_{B}\right), \end{array}$$

where $V_{A}$ and $V_{B}$ are local confining potentials for electrons A and B, while Coulomb interaction between electrons $V_{A-B}\left(x_{A},y_{A},x_{B},y_{B}\right)=\frac{q^{2}}{d(\left(x_{A},y_{A}\right),\left(x_{B},y_{B}\right))}$ and $\left(s_{A},s_{B}\right)$ is a pair of variables parametrising the two-dimensional two-body wavefunction. The case of two-body interaction is considered by omitting spin degrees of freedom. If each nanowire (bent or straight) is divided into *m* pieces, we have a Hamiltonian matrix of size $M^{p}$ by $M^{p}$ that has $M^{p=2}$ energy eigenfunctions and eigenvalues. We set the number of particles p to 2 (for q-swap gate *p* = 2 and *p* = 3 for CNOT gate). The interacting particles are represented by the number of electrons placed at different open-loop semiconductor nanowires. We set *M* = 7 for two V lines symmetric around OX axes and assume $\alpha_{A}=\alpha_{B}=c$ with formulas for cables A and B given by $F_{A(B)}\left(x\right)=a+b*{\left(Tanh\left(c*x+d\right)\right)}^{2}$, so the nanolines are given by $\left(x,F_{1}\left(x\right)\right)$ and $\left(x,F_{2}\left(x\right)\right)$. as depicted in Figure 4.

We obtained the probability distribution for the case of three built-in q-wells (dots) depicted on the left in Figure 13 and for the case of no built-in q wells depicted on the right in Figure 13. We can trace electron anticorrelation under different nanowire cable bending. We can identify anticorrelation and correlation factors as occurring in the case of electrostatically interacting electrons placed at different nanocables, as indicated already in [14]. For the purposes of computation, in this work all eigenenergies were set with equal probability of occupancy. Quite clearly, the presented results go beyond the tight-binding model expressed by [14,16].

## 6. Classical Description of a Physical System Implementing a Wannier Qubit Swap Gate

Referring to Figure 4, we have the Hamiltonian describing the interaction of electrons confined in different nanowires of the structure

(119) $$\begin{array}{c} H=H_{1}+H_{2}+H_{1-2}=\\ =\frac{{\left(p_{1,x}\right)}^{2}}{2m_{1}}+\frac{{\left(p_{1,y}\right)}^{2}}{2m_{1}}+\frac{{\left(p_{2,x}\right)}^{2}}{2m_{2}}+\frac{{\left(p_{2,y}\right)}^{2}}{2m_{2}}+V\left(x_{1}\left(s_{1}\right),y_{1}\left(s_{1}\right)\right)+V\left(x_{2}\left(s_{2}\right),y_{2}\left(s_{2}\right)\right)+H_{C}\left(s_{1},s_{2}\right), \end{array}$$

and we have

(120) $$\begin{array}{c} \frac{d}{dx_{1}}=\frac{ds_{1}}{dx_{1}}\frac{d}{ds_{1}},\frac{d}{dy_{1}}=\frac{ds_{1}}{dy_{1}}\frac{d}{ds_{1}},\\ \frac{d}{dx_{2}}=\frac{ds_{2}}{dx_{2}}\frac{d}{ds_{2}},\frac{d}{dy_{2}}=\frac{ds_{2}}{dy_{2}}\frac{d}{ds_{2}},\\ \frac{d}{dt}x_{1}=\frac{dx_{1}}{ds_{1}}\frac{d}{dt}s_{1},\frac{d}{dt}y_{1}=\frac{dy_{1}}{ds_{1}}\frac{d}{dt}s_{1},\\ \frac{d}{dt}x_{2}=\frac{dx_{1}}{dx_{1}}\frac{d}{dt}s_{2},\frac{d}{dt}y_{2}=\frac{dy_{2}}{ds_{2}}\frac{d}{dt}s_{2},\\ \frac{1}{m_{1}}\frac{d}{dt}p_{1,x}=\frac{d^{2}}{dt^{2}}x_{1}=\frac{dx_{1}}{ds_{1}}\frac{d}{dt}\left[\frac{dx_{1}}{ds_{1}}\frac{d}{dt}s_{1}\right]=\left[{\left(\frac{dx_{1}}{ds_{1}}\right)}^{2}\frac{d^{2}}{dt^{2}}s_{1}+\frac{d^{2}x_{1}}{ds_{1}^{2}}\frac{dx_{1}}{ds_{1}}\frac{d}{dt}s_{1}\right], \end{array}$$

We set $V\left(s_{1},s_{2}\right)=q^{2}/{\left({\left(x_{1}\left(s_{1}\right)-x_{2}\left(s_{2}\right)\right)}^{2}+{\left(f_{1}\left(x_{1}\left(s_{1}\right)\right)-f_{2}\left(x_{2}\left(s_{2}\right)\right)\right)}^{2}\right)}^{1/2}$ and thus obtain $\frac{d}{dx1}H=\frac{ds_{1}}{dx_{1}}\frac{d}{ds_{1}}H$ and $\frac{d}{dx2}H=\frac{ds_{2}}{dx_{1}}\frac{d}{ds_{2}}H$.

**Figure 14:**
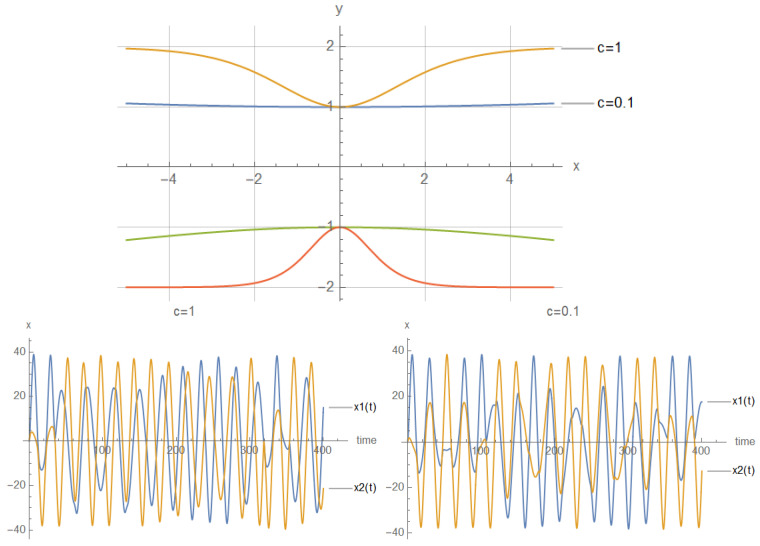
(**Upper**): Two particles (electrons) in semiconductor nanowires interacting electrostatically in classical picture and family of V-shaped lines parametrized by $F_{1(2)}\left(x_{1(2)}\right)=a+b*{\left(Tanh\left(c*x_{1(2)}+d\right)\right)}^{2}$. (**Lower**): The nanoline trajectory of the first particle is given in blue by $\left(x_{1},F_{1}\left(x_{1}\right)\right)$ and of the second particle in orange $\left(x_{2},F_{2}\left(x_{2}\right)\right)$.

Let us solve the practical set of coupled non-linear ODE equations by setting $s_{1}=x_{1}$ and $s_{2}=x_{2}$, so we have

(121) $$\begin{array}{ccc} m_{1}\frac{d^{2}}{dt^{2}}x_{1}\left(t\right) & = & -m_{1}\left(\frac{d}{dx_{1}}f_{1}\left(x_{1}\right)\right)\left(\frac{d^{2}}{dx_{1}^{2}}f_{1}\left(x_{1}\right)\right){\left(\frac{d}{dt}x_{1}\left(t\right)\right)}^{2}-\frac{d}{dx_{1}}V_{1}\left(x_{1},f_{1}\left(x_{1}\right)\right)-\frac{d}{dx_{1}}\frac{q^{2}}{{\left({\left(x_{1}-x_{2}\right)}^{2}+\left(f_{1}\left(x_{1}\right)-f_{2}\left(x_{2}\right)\right)\right)}^{\frac{1}{2}}},\\ m_{2}\frac{d^{2}}{dt^{2}}x_{2}\left(t\right) & = & -m_{2}\left(\frac{d}{dx_{2}}f_{2}\left(x_{2}\right)\right)\left(\frac{d^{2}}{dx_{2}^{2}}f_{2}\left(x_{2}\right)\right){\left(\frac{d}{dt}x_{2}\left(t\right)\right)}^{2}-\frac{d}{dx_{2}}V_{2}\left(x_{2},f_{2}\left(x_{2}\right)\right)-\frac{d}{dx_{2}}\frac{q^{2}}{{\left({\left(x_{1}-x_{2}\right)}^{2}+\left(f_{1}\left(x_{1}\right)-f_{2}\left(x_{2}\right)\right)\right)}^{\frac{1}{2}}}, \end{array}$$

where $V_{1}\left(x_{1}\left(t\right),y_{1}\left(t\right)\right)$ and $V_{2}\left(x_{2}\left(t\right),y_{2}\left(t\right)\right)$ are local confining potentials in nanowires. In particular, we have set $V_{1}\left(x_{1}\right)=e^{0.1\sqrt{x_{1}^{2}+{\left(f_{1}\left(x_{1}\right)\right)}^{2}}}$ and $V_{2}\left(x_{2}\right)=e^{0.1\sqrt{x_{2}^{2}+{\left(f_{2}\left(x_{2}\right)\right)}^{2}}}$ with $f_{1}\left(x_{1}\right)=1+Tanh{\left(c0.5x_{1}\right)}^{2}$ and $f_{2}\left(x_{2}\right)=1+Tanh{\left(cx_{2}\right)}^{2}$. Additional “dissipative” terms occur in the equations of motion, expressed by $-m_{1}\left(\frac{d}{dx_{1}}f_{1}\left(x_{1}\right)\right)\left(\frac{d^{2}}{dx_{1}^{2}}f_{1}\left(x_{1}\right)\right){\left(\frac{d}{dt}x_{1}\left(t\right)\right)}^{2}$ and $-m_{2}\left(\frac{d}{dx_{2}}f_{2}\left(x_{2}\right)\right)\left(\frac{d^{2}}{dx_{2}^{2}}f_{2}\left(x_{2}\right)\right){\left(\frac{d}{dt}x_{2}\left(t\right)\right)}^{2}$, due to the non-zero curvature of the nanocable. The emergence of deterministic chaos is depicted in Figure 14. A wider consideration of various coordinate systems and curved semiconductor nanowires is given in [25].

## 7. Conclusions and Further Perspectives

The use of hopping terms in a tight-binding model (2) was justified by Schrödinger formalism with Formulas (19)–(23) for a static electric and magnetic field, as well as for the case of Rabi oscillations and non-static electric and magnetic fields, expressed by Formula (47). The prescription for the exact computation of localized energy terms $E_{p1}$ and $E_{p2}$ is given by Formula (4). The origin of tight-binding model dissipation [29,30] was identified in the framework of Schrödinger formalism, since the real value eigenergies of a two-quantum dot system ($E_{1}$, $E_{2}$) can be replaced with $\left(E_{1r}+iE_{1i},E_{2r}+iE_{2i}\right)$, where ($E_{1i}$,$E_{2i}$) are dissipative terms and ($E_{1r}$,$E_{2r}$) are real-valued non-dissipative terms (originally given by a Schrödinger equation). The mathematical structure of the dissipative tight-binding model in the static case with a constant electric and magnetic field is given by (24) and in the case of Rabi oscillation by (52). The concept of Wannier functions in a system of two coupled semiconductor qubits (Wannier qubits) can be applied not only for single-electron occupancy, but also for many electron occupancy. In such cases, the fermion Hubbard model can be used, as given in [20,31]. Various correlation functions using Schrödinger formalism and justifying the tight-binding model have been proposed and are presented in the extended version of this work [25] using [32,33]. The case of two-Wannier qubit interaction was formulated in Schrödniger formalism using straight and curvy semiconductor nanowires and basic preliminary numerical results were presented in Figure 13. Consequently, we can derive the Hermitian and non-Hermitian Hamiltonian matrix formula. The effect or curvature of semiconductor quasi-one-dimensional nanowires was expressed by equations of motion, in both the classical and quantum regime. The main conclusion is that bending a nanowire has the effect of separating its two reservoirs, as depicted in Figure 7. This has importance for future photonic technologies [34]. This allows for modeling of quantum and classical SWAP gates using electron–electron interaction. The effects of the topology of open-loop semiconductor nanowires can be studied by using the Toeplitz matrix approach in different coordinate systems, such as Cartesian, cylindrical, and spherical coordinate systems.

The presented approach allows for the description of Wannier position-based qubits with single and many electrons injected into the source and draining of the field effect transistor. They also provide a basis for modeling quantum neural networks implemented by a chain of coupled quantum dots. The fundamental approach presented in this paper is useful for enhancing the tight-binding scheme used in the design of quantum gates [23,27,29,35].

The presented work is an extension of methodology described by [17], as well as by [1,23,26]. The results given in Figure 5, Figure 6, Figure 7, Figure 8, Figure 9, Figure 10, Figure 11 and Figure 12 for the case of Tanh square cables should be tested using Local Density of States observed in STM for different α coefficients. Various physical phenomena observed in condensed matter systems [20] can be simulated with the concept of quantum programmable matter, demonstrated by position-dependent qubits controlled by electric signals [35,36]. Furthermore, quantum machine learning can mimic any stochastic finite state machine by using the tight-binding model, as shown in [29].

**Figure 15:**
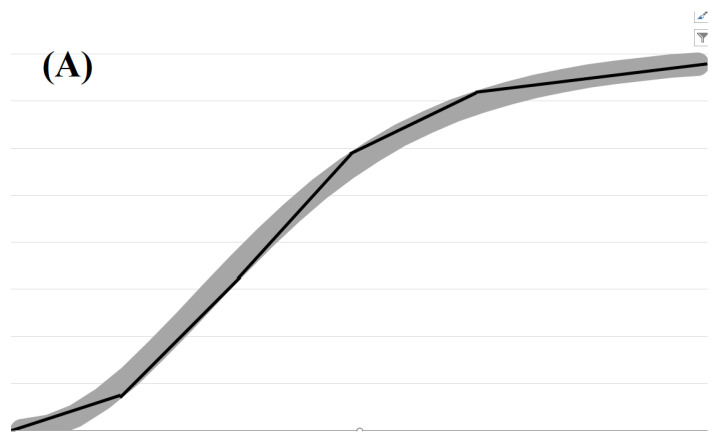

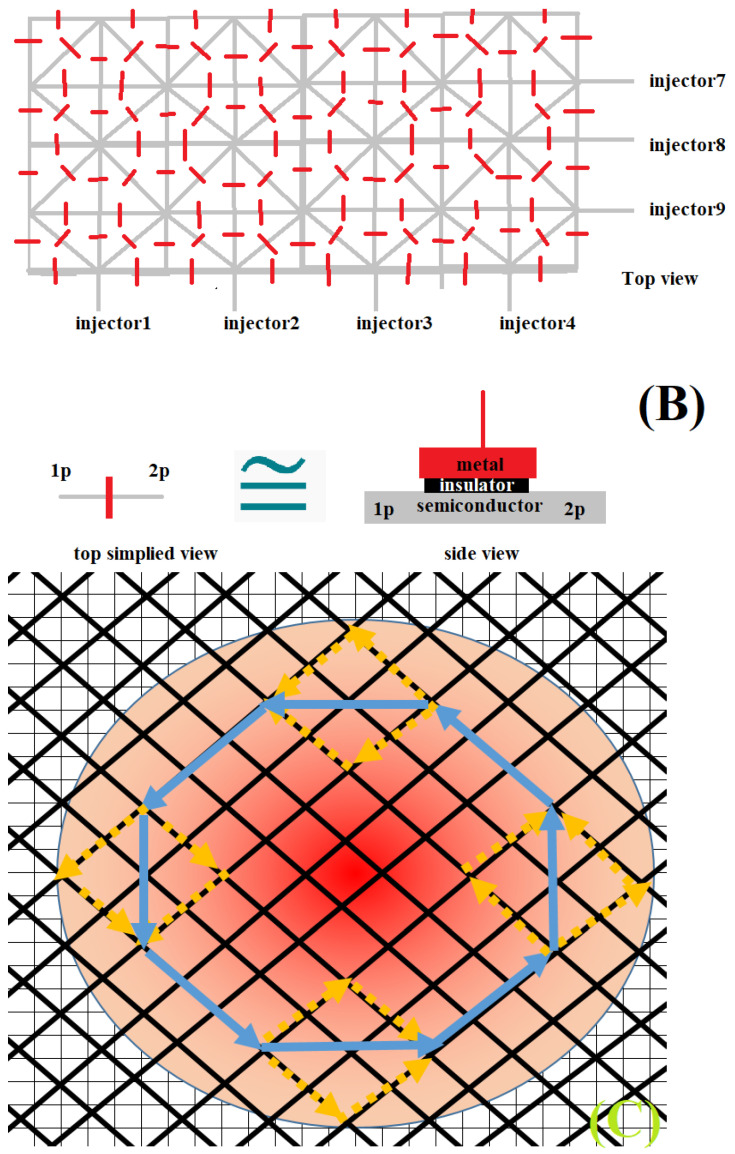
Arbitrary curved quasi-one-dimensional nanowire in two dimensions can be approximated by a finite number of straight nanowires (**A**) that can mimic a varied class of tunable metrics that is implemented with a two-dimensional chain of nanowires (**B**) with specified single-electron injectors. In such a way, an electrostatically programmable space-metric for single-electrons in discretized space accounting for bounded or unbounded states can emerge and be technologically controlled on a massive scale as specified in (**C**). Self-interference effects for electron/hole propagating from one geometrical point to another point may occur as indicated in (**C**) as it is a confirmation of quantum particle propagation along infinite number of possible trajectories between two points in space. Visualization of (**A**,**C**) was enhanced by Marcin Piontek.

Using the concept of a reconfigurable q-graph of quantum dots [16], we can simulate the behaviour of a quantum particle in curved space. This will be the subject of future work [28,37,38]. Another avenue for future work is the investigation of holonomic quantum computation [39,40] with qubits constructed from curvy semiconductor nanowires as described in this work. Curved nanowires could be expressed by more refined models, as given by [41]. From the nanotechnological perspective, it seems very challenging but possible to create the electrostatically programmable two-dimensional metric depicted in Figure 15. The main concept behind this discretized metric is simple. Single electrons are injected into a chain of semiconductor nanowires, forming a symmetric periodic lattice. Connectivity between certain areas of the nano-lattice is controlled by applying a voltage to the metallic gate placed at the top of each semiconductor nanowire and separated by an insulator. We can force wave-packet propagation along a limited number of trajectories of a certain shape by blocking certain directions and enabling other directions. Quite obviously, the presented concept is not a full solution for a programmable metric, but an approximated solution performed with the implementation of a voltage-controlled metric governing the movement of single electrons or conglomerates of single electrons. The Schrödinger wave-packets have low energies; as for high energies, quasi-one-dimensional approximation might be less valid. We can create bound states and quasi-two-dimensional atoms (mimicking real atoms or molecules) by means of electrons moving on the class of closed trajectories around gates with opposite charge (in the core encircled by the trajectory of moving electrons/holes). One expects richness in the dynamical behaviors that can occur in such systems what indicated the subject of further study. The presented lattice can be treated as a discretized version of the Schrödinger equation in two dimensions.

The most important conclusion of this work is that by bending a semiconductor or superconducting nanowire we can generate effective barriers in a semiconductor or superconductor, which can be penetrated by electrons or Cooper pairs via the tunneling process. Therefore, one does not need an external electric or magnetic field to create an effective barrier, which simplifies nanostructures and thus contributes to the field of programmable quantum matter. Such geometric tunability is formally expressed by the derived geometric Aharonov–Bohm effect in the case of the presence or lack of a vector potential field. Therefore, the presented methodology and results have important implications for the design of future experiments, with semiconductor nanowires implementing single- or non-single electron devices (as in case of Wannier qubits), as well as with superconducting nanowires implementing new types of Josephson junctions.

## Data Availability

The original contributions presented in the study are included in the article, further inquiries can be directed to the corresponding author.

## References

[1] Giounanlis P., Blokhina E., Pomorski K., Leipold D.R., Staszewski R.B. (2019). Modeling of Semiconductor Electrostatic Qubits Realized Through Coupled Quantum Dots. IEEE Access.

[2] Pomorski K., Giounanlis P., Blokhina E., Leipold D., Peczkowski P., Staszewski R.B. From two types of electrostatic position-dependent semiconductor qubits to quantum universal gates and hybrid semiconductor-superconducting quantum computer. Proceedings of the Superconductivity and Particle Accelerators.

[3] Ridene S. (2017). Novel T-shaped GaSb/InAsN quantum wire for mid-infrared laser applications. Phys. Lett. A.

[4] Ridene S. (2018). Mid-infrared emission in In_x_GaAs_1-x_/GaAs T-shaped quantum wire lasers and its indium composition dependence. Infrared Phys. Technol..

[5] Likharev K.K. (1999). Single-Electron Devices and Their Applications. Proc. IEEE.

[6] Fujisawa T., Hayashi T., Cheong H.D., Jeong Y.H., Hirayama Y. (2004). Rotation and phase-shift operations for a charge qubit in a double quantum dot. Phys. E Low-Dimens. Syst. Nanostruct..

[7] Petersson K.D., Petta J.R., Lu H., Gossard A.C. (2010). Quantum coherence in a one-electron semiconductor charge qubit. Phys. Rev. Lett..

[8] Leipold D. (2018). Controlled Rabi Oscillations as Foundation for Entangled Quantum Aperture Logic. Proceedings of the Seminar at UC Berkley Quantum Labs.

[9] MacQuarrie E.R., Neyens S.F., Dodson J.P., Corrigan J., Thorgrimsson B., Holman N., Palma M., Edge L.F., Friesen M., Coppersmith S.N. (2020). Progress toward a capacitively mediated CNOT between two charge qubits in Si/SiGe. npj Quantum Inf..

[10] Weichselbaum A., Ulloa S.E. (2004). Charge qubits and limitations of electrostatic quantum gates. Phys. Rev. A.

[11] Lee N., Tsuchiya R., Shinkai G., Kanno Y., Mine T., Takahama T., Mizokuchi R., Kodera T., Hisamoto D., Mizuno H. (2020). Enhancing electrostatic coupling in silicon quantum dot array by dual gate oxide thickness for large-scale integration. Appl. Phys. Lett..

[12] Pauka S.J., Das K., Kalra R., Moini A., Yang Y., Trainer M., Bousquet A., Cantaloube C., Dick N., Gardner G.C. (2021). A cryogenic CMOS chip for generating control signals for multiple qubits. Nat. Electron..

[13] Pomorski K. (2020). Seminars on Quantum Technologies at YouTube Channel: Quantum Hardware Systems. https://www.youtube.com/watch?v=Bhj_ZF36APw.

[14] Pomorski K., Giounanlis P., Blokhina E., Leipold D., Staszewski R.B. (2019). Analytic view on Coupled Single-Electron Lines. Semicond. Sci. Technol..

[15] Bashir I., Asker M., Cetintepe C., Leipold D., Esmailiyan A., Wang H., Siriburanon T., Giounanlis P., Blokhina E., Pomorski K. A Mixed-Signal Control Core for a Fully Integrated Semiconductor Quantum Computer System-on-Chip. Proceedings of the ESSCIRC 2019—IEEE 45th European Solid State Circuits Conference (ESSCIRC).

[16] Pomorski K.D., Peczkowski P., Staszewski R.B. (2020). Analytical solutions for N interacting electron system confined in graph of coupled electrostatic semiconductor and superconducting quantum dots in tight-binding model. Cryogenics.

[17] Xu H.Q. (2002). Method of calculations for electron transport in multiterminal quantum systems based on real-space lattice models. Phys. Rev. B.

[18] Liang Z.X., Hu B.B., Wu B. (2009). Interaction Effects on Wannier Functions of a Bose-Einstein Condensate in an Optical Lattice and Implications for Bose-Hubbard Model. arXiv.

[19] Marzari N., Yates R.J., Souza I., Vanderbilt D. (2012). Maximally localized Wannier functions: Theory and applications. Rev. Mod. Phys..

[20] Spalek J. (2015). Wstep do Fizyki Materii Skondensowanej.

[21] Aharonov Y., Bohm D. (1959). Significance of Electromagnetic Potentials in the Quantum Theory. Phys. Rev..

[22] https://quantumtech.blog/2022/10/20/quantum-computing-modalities-a-qubit-primer-revisited/.

[23] Pomorski K. (2023). Analytical Solutions for N-Electron Interacting System Confined in Graph of Coupled Electrostatic Semiconductor and Superconducting Quantum Dots in Tight-Binding Model with Focus on Quantum Information Processing. Nanomaterials and Nanocomposites, Nanostructure Surfaces, and Their Applications.

[24] Pomorski K. (2020). Analytical View on Non-Invasive Measurement of Moving Charge by Position Dependent Semiconductor Qubit. Proceedings of the Future Technologies Conference 2020.

[25] Pomorski K. (2021). Fundamental description of Wannier qubits of any topology in semiconductor by analytical and numerical computations. arXiv.

[26] Szafran B. (2020). Paired electron motion in interacting chains of quantum dots. Phys. Rev. B.

[27] Pomorski K. (2020). Analytical view on tunnable electrostatic Quantum Swap Gate in tight-binding model. arXiv.

[28] da Costa R.C.T. (1981). Quantum mechanics of a constrained particle. Phys. Rev. A.

[29] Pomorski K. (2020). Equivalence between classical epidemic model and non-dissipative and dissipative quantum tight-binding model. arXiv.

[30] Pomorski K. (2021). Analytic view on N body interaction in electrostatic quantum gates and decoherence effects in tight-binding model. Int. J. Quantum Inf..

[31] Jinbin L., Yue Y., Dudarev A.M. (2006). Interaction broadening of Wannier functions and Mott transitions in atomic BEC. New J. Phys..

[32] Moodie J.C., Long M.W. (2020). An exact power series representation of the Baker–Campbell–Hausdorff formula. J. Phys. A Math. Theor..

[33] Casas F., Murua A., Nadinic M. (2012). Efficient computation of the Zassenhaus formula. Comput. Phys. Commun..

[34] Wang J., Sciarrino F., Laing A., Thompson M.G. (2020). Integrated photonic quantum technologies. Nat. Photonics.

[35] Pomorski K., Staszewski R.B. (2020). Towards quantum internet and non-local communication in position-based qubits. AIP Conf. Proc..

[36] Jaynes E.T., Cummings F.W. (1963). Comparison of quantum and semiclassical radiation theories with application to the beam maser. Proc. IEEE.

[37] Exirifarda Q., Karimia E. (2021). Schrödinger equation in a general curved space-time geometry. arXiv.

[38] Du L., Wang Y.L., Liang G.H., Kang G.Z., Zong H.S. (2016). Schrödinger Equation of a Particle on a Rotating Curved Surface. Chin. Phys. Lett..

[39] Zhang C., Chen T., Wang X., Xue Z. (2021). Microwave driven geometric quantum computation on semiconductor charge qubits. Adv. Quantum Technol..

[40] Duan L.M., Cirac J.I., Zoller P. (2001). Geometric Manipulation of Trapped Ions for Quantum Computation. arXiv.

[41] Stepien L.T. (2020). Bogomolny equations for the BPS Skyrme models with impurity. New J. Phys..

